# Supplementary material for: Decoding non-coding SNPs: systems genomics modelling dissects the heterogeneity of IBD
Source: Mol Syst Biol. 2025 Nov 26;22(2):259–80. doi: 10.1038/s44320-025-00169-3 (PMC12864814; doi:10.1038/s44320-025-00169-3)
Supplement: Supplementary file 1 — Appendix [file 44320_2025_169_MOESM1_ESM.pdf]

Appendix for Decoding Non-coding SNPs: Systems Genomics Modelling Dissects the Heterogeneity of IBD

Table of Contents

Appendix Table S1: Patient demographics 3

Appendix Table S2: Transcriptomic validation of the network propagation models 4

Appendix Figure S1. Number of features per propagation steps and disease comparing various data sources. 5

Appendix Figure S2. Determining the number of patient clusters in Crohn's disease and ulcerative colitis using k means clustering. 6

Appendix Figure S3. Validation in Crohn's disease patients from Krzak et al dataset .. 7

Appendix Figure S4. Regulatory analysis of transcription factors in Crohn's disease patients from Kong et al dataset 8

Appendix Figure S5. Cell type-specific overrepresentation in ulcerative colitis patients from the Smillie et al dataset 9

Appendix Figure S6. A) Combined SNP-propagated regulatory network for Crohn's disease and ulcerative colitis.B) Comparing Jaccard distance between ulcerative colitis and Crohn's disease patients in the signalling and regulatory networks 10

Appendix Figure S7. Hierarchical clustering of the SNP-affected proteins, signalling network and gene regulatory network in ulcerative colitis and Crohn's disease......... 11

Appendix Figure S8. Comparing various network resources for network propagation 12

Appendix Figure S9. Patient clusters are independent of the used network in ulcerative colitis but have network-specific outcomes in Crohn's disease 14

Appendix Figure S10. Similarity between degree-matched and non-degree-matched controls at signalling and regulatory layers using various network resources. A) Crohn's disease B) Ulcerative colitis... 16

Appendix Figure S11. Using degree-matched algorithm compared to non-degree-matched algorithm results in similar clustering in ulcerative colitis but different outcomes in Crohn's disease. 17

Appendix Table S1: Patient demographics

	Sex		Age at diagnosis	
	Male	Female	Mean	STD
Ulcerative colitis	505	397	33.78	14.31
Crohn's disease	704	991	27.22	11.82

Appendix Table S2: Transcriptomic validation of the network propagation models

Disease	Comparison	Citation	GSE ID	SNP-affected proteins *	SNP-propagated signalling network (>100 patients) *	SNP-propagated gene regulation network (>100 patients) *
Crohn's disease	Ileal mucosal biopsies of non-inflamed CD vs healthy control	(Vancamelbeke et al, 2017)	GSE75214	1/149<br>0.8302189	2/149<br>0.7786362	32/266<br>0.000349404
	Ileal mucosal biopsies of inflamed CD vs healthy control	(Verstockt et al, 2019)	GSE102133	2/638<br>0.8302189	5/638<br>0.7786362	116/1149<br>5.64E-07
	Rectal biopsies of moderate to severe CD vs healthy control	(Pavlidis et al, 2021)	GSE207022	2/279<br>0.8302189	3/279<br>0.7786362	59/468<br>5.64E-07
Ulcerative colitis	Colonic mucosa, untreated UC vs healthy control	(Arijs et al, 2018)	GSE73661	21/1530<br>0.0459	30/1528<br>0.042142682	498/2936<br>1.26575E-07
	Colonic biopsies near ulcers in UC vs healthy control	(Van der Goten et al, 2014)	GSE48958	21/1561<br>0.0459	32/1509<br>0.032672974	103/689<br>0.269585878
	Colonic mucosa, inactive UC vs healthy control	(Vancamelbeke et al, 2017)	GSE75214	3/306<br>0.654	5/306<br>0.534229924	81/581<br>0.498756592
	Colonic mucosa, inflamed UC vs healthy control			21/1561<br>0.0459	31/1561<br>0.042142682	524/299<br>1.55665E-09

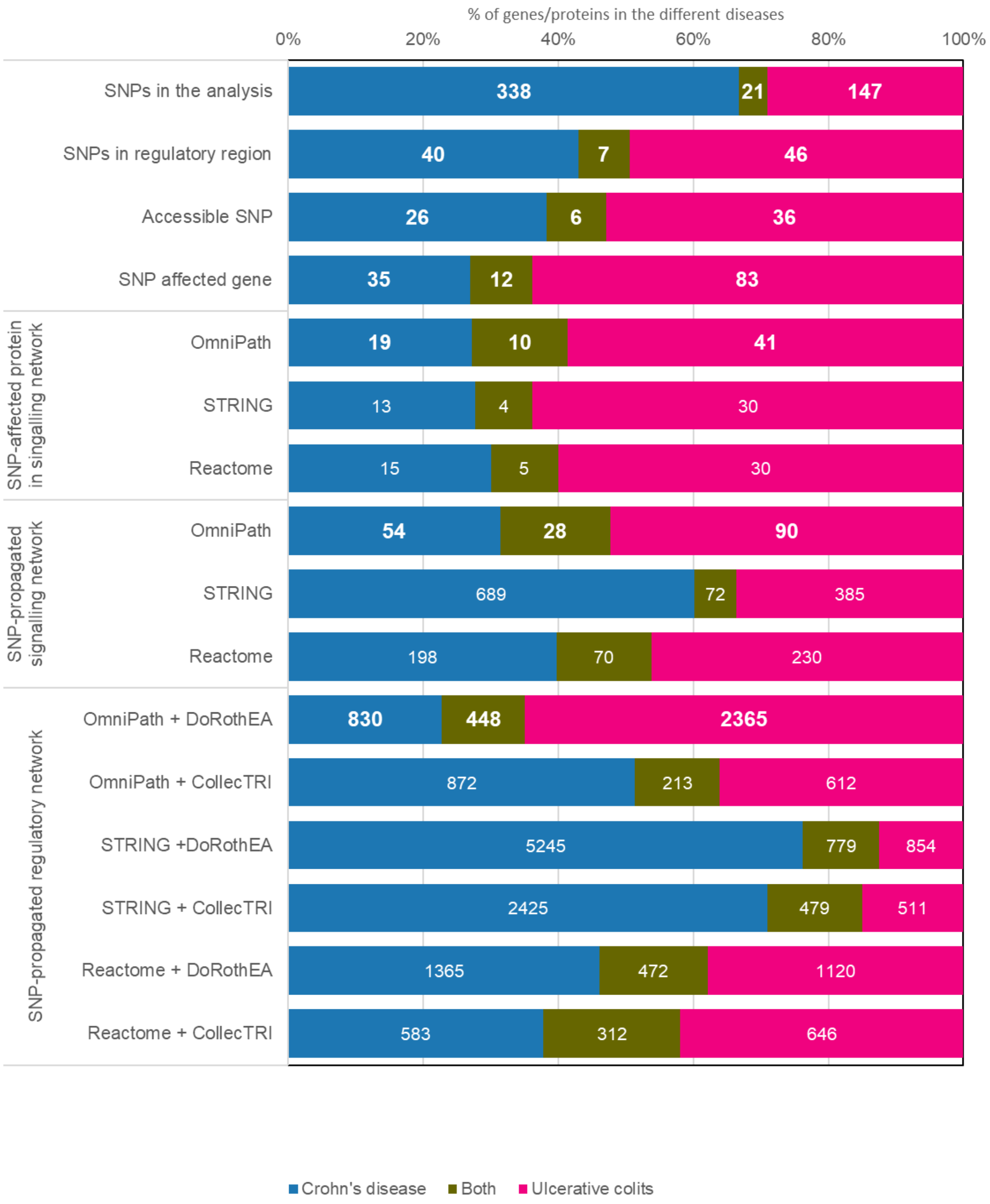

Appendix Figure S1. Number of features per propagation steps and disease comparing various data sources.

The number of SNPs in the cohort of Crohn's disease and ulcerative colitis patients, along with the corresponding number of SNP-affected genes/proteins and SNP-propagated genes in the downstream signalling and regulatory networks.

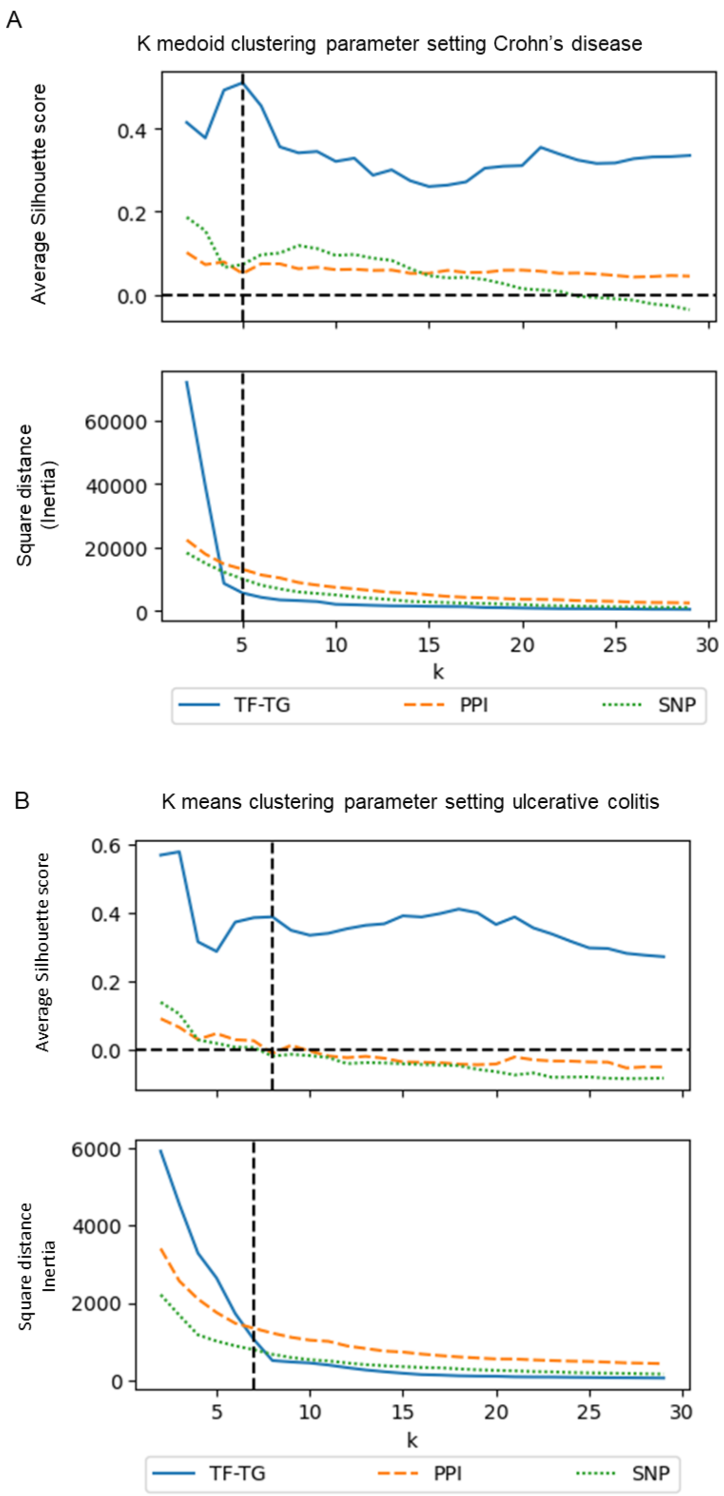

Appendix Figure S2. Determining the number of patient clusters in Crohn's disease and ulcerative colitis using k means clustering.

A) Crohn's disease B) Ulcerative colitis. Each figure contains the SNP-affected genes, the PPI network and the principal component of the regulatory networks input for clustering. The regulatory networks have the highest average silhouette score and the steepest decline in inertia.

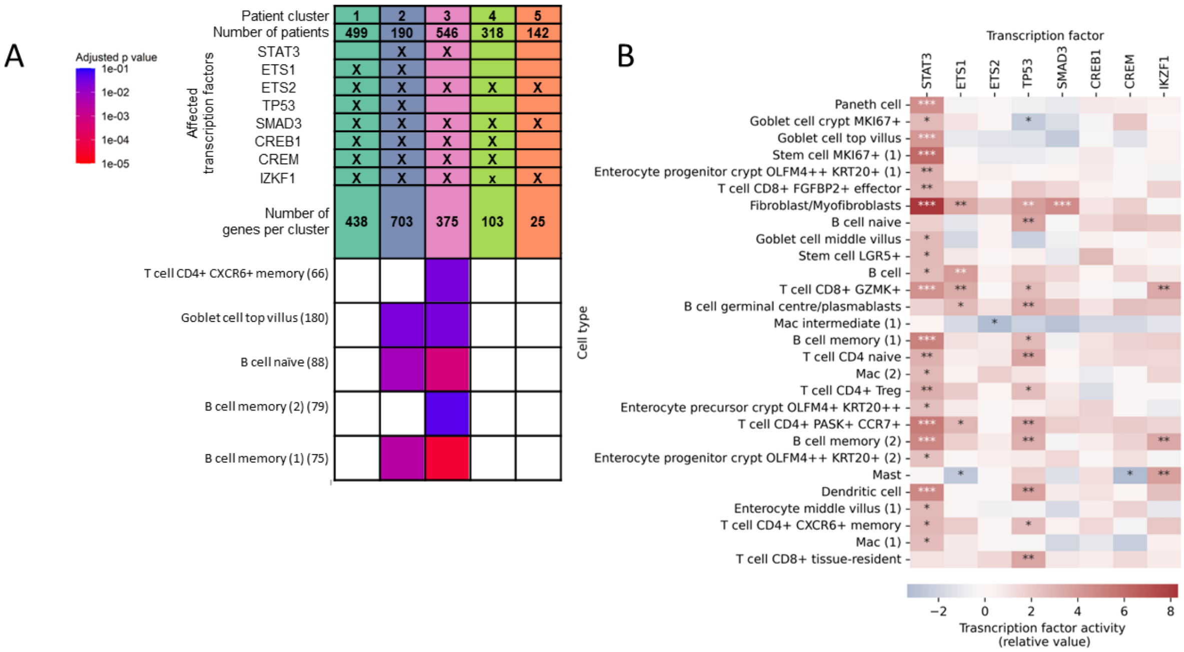

Appendix Figure S3. Validation in Crohn’s disease patients from Krzak et al dataset

A) Differentially expressed genes are overrepresented in the various patient clusters. Figure legend is directly comparable and same as Figure 4. Only a few cell types are overrepresented. B) Transcription factor activity is cell type specific. Benjamini Hochberg adjusted p value * <0.05 **<0.01, ***<0.001, fitted slope of univariate linear model. Most SNP-propagated transcription factors have cell type-specific activities.

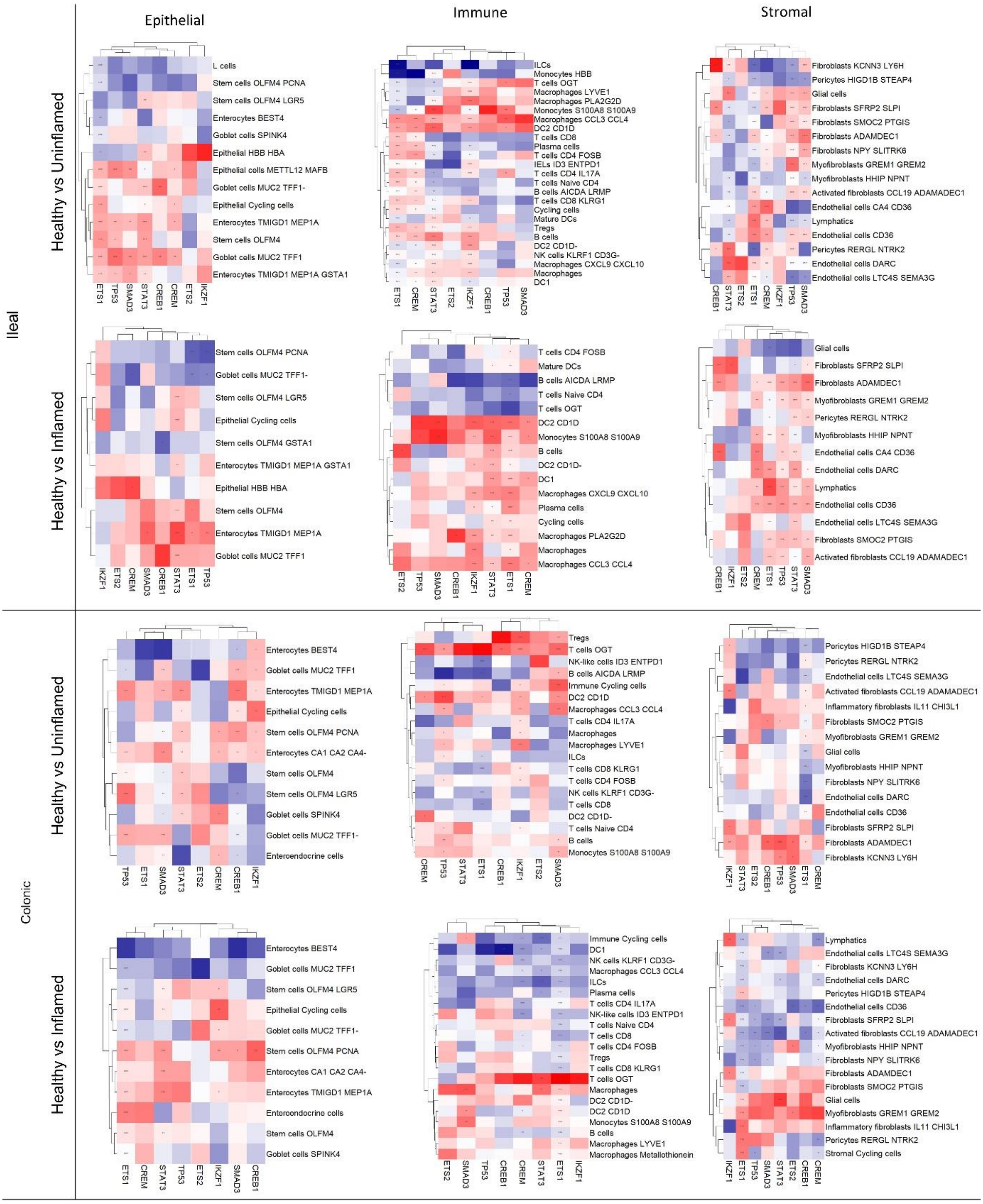

Appendix Figure S4. Regulatory analysis of transcription factors in Crohn's disease patients from Kong et al dataset

Each subfigure represents a subset of cells in the Kong et al dataset. Red means active transcription factor in CD comparing the CD non-inflamed and healthy conditions meanwhile blue means active in healthy condition. TFs. Benjamini Hochberg adjusted p-value * <0.05 **<0.01, ***<0.001, fitted slope of univariate linear model.

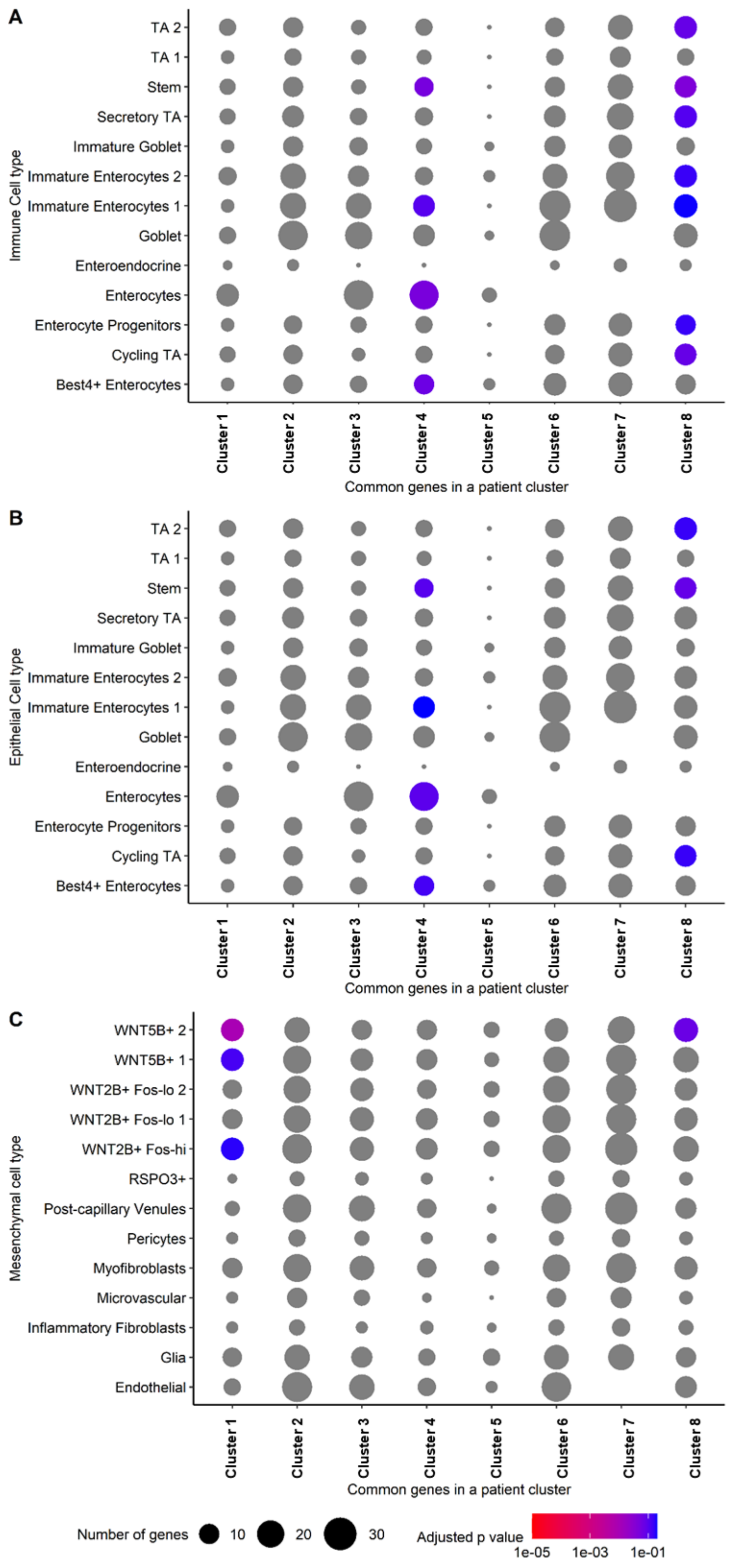

Appendix Figure S5. Cell type-specific overrepresentation in ulcerative colitis patients from the Smillie et al dataset

A) Immune cells, B) Epithelial cells C) Mesenchymal cells. Differentially expressed genes are overrepresented in the various patient clusters. Clusters 1,4 and 8 corresponded with UC cell type-specific signatures obtained from the Smillie et al single-cell dataset.

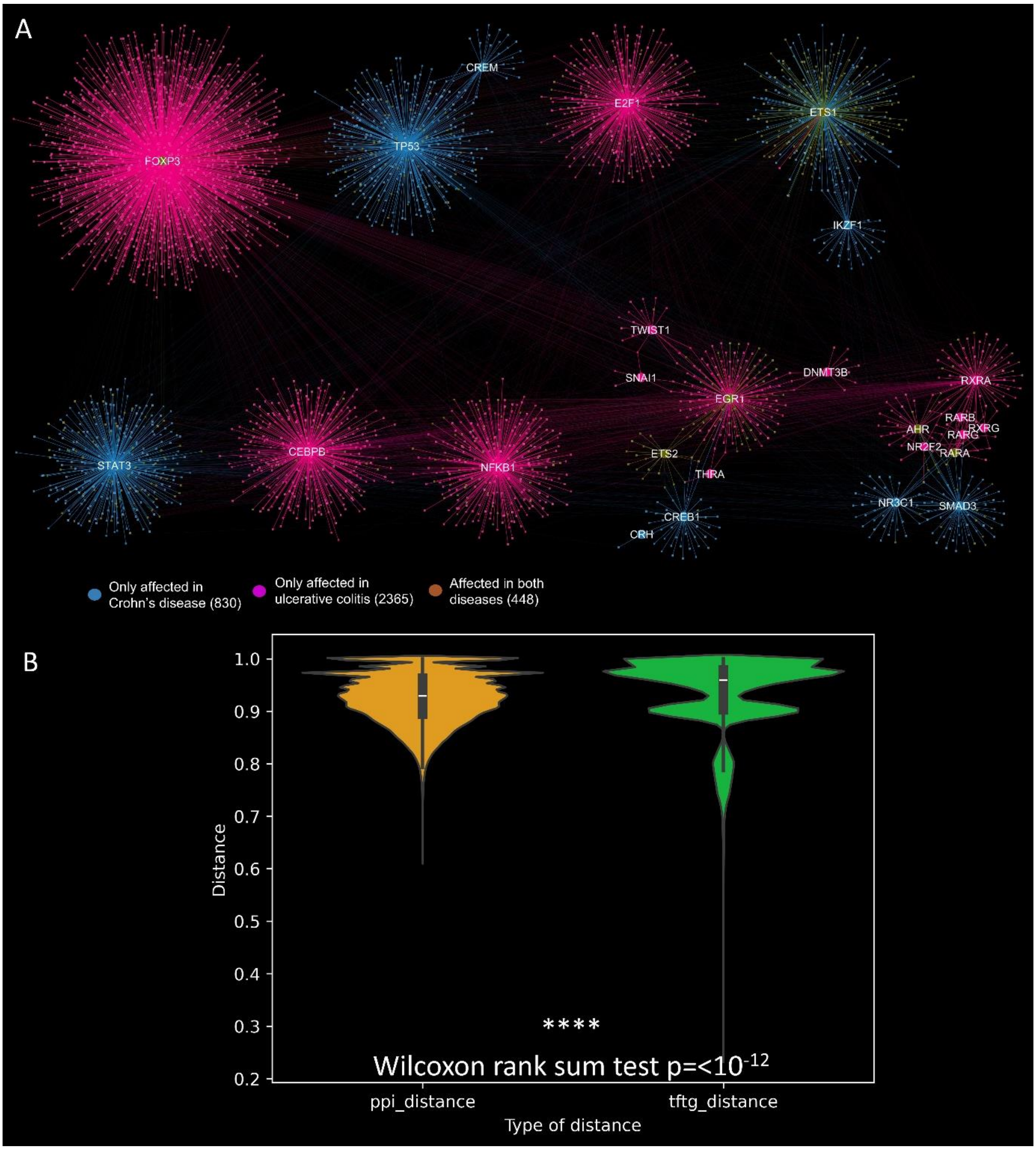

Appendix Figure S6.

A) Combined SNP-propagated regulatory network for Crohn's disease and ulcerative colitis. The network is modularised using the Girvan-Newmann algorithm in Cytoscape. Most modules are disease-specific. However, two modules around ETS1 and a module containing ETS2, contain transcription factors and target genes that are present in both ulcerative colitis and Crohn's disease patients.

B) Comparing Jaccard distance between ulcerative colitis and Crohn's disease patients in the signalling and regulatory networks. The distance in the regulatory network is significantly larger.

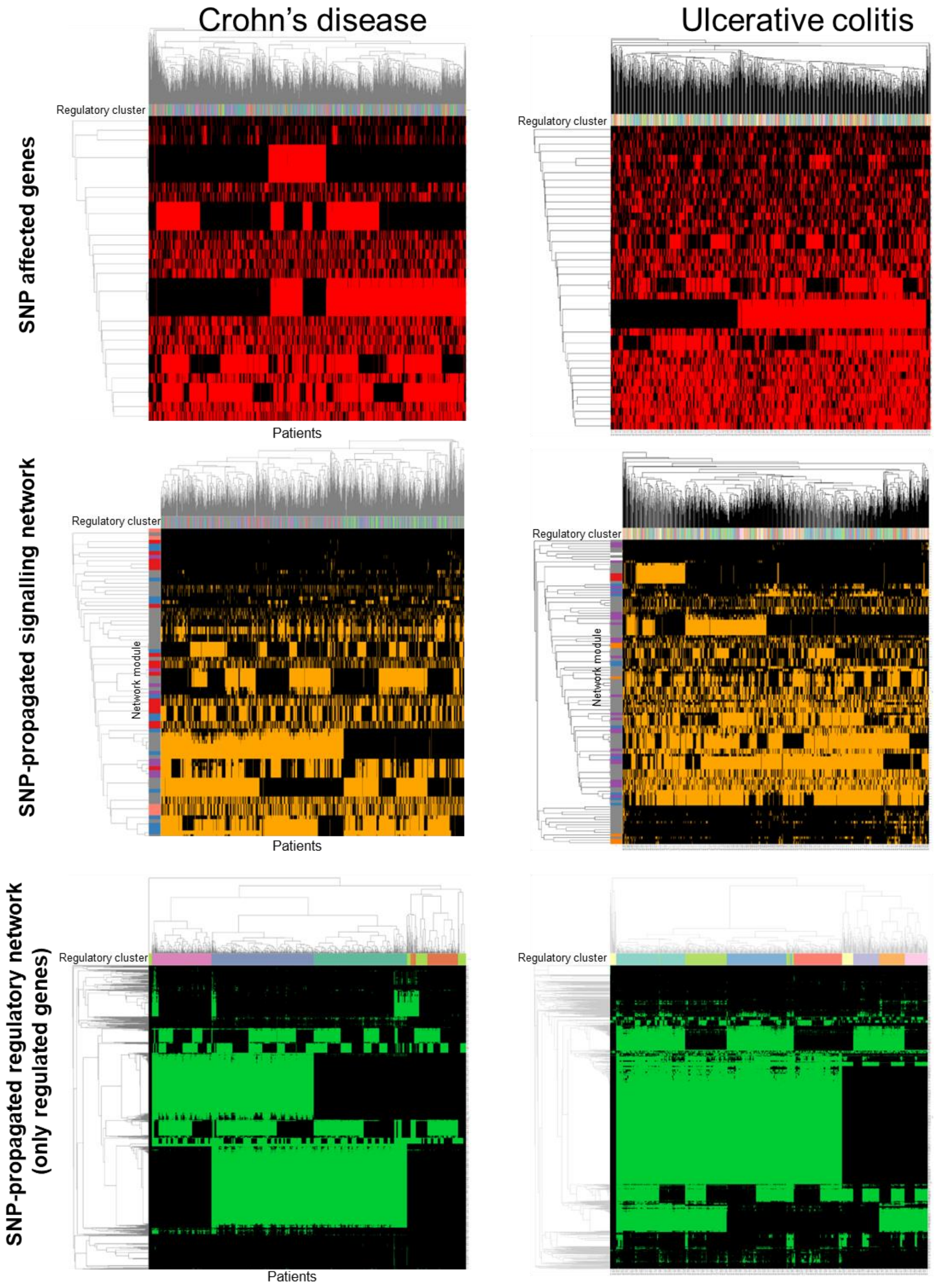

Appendix Figure S7. Hierarchical clustering of the SNP-affected proteins, signalling network and gene regulatory network in ulcerative colitis and Crohn's disease.

The regulatory network gives clear clustering based on the perturbed hubs.

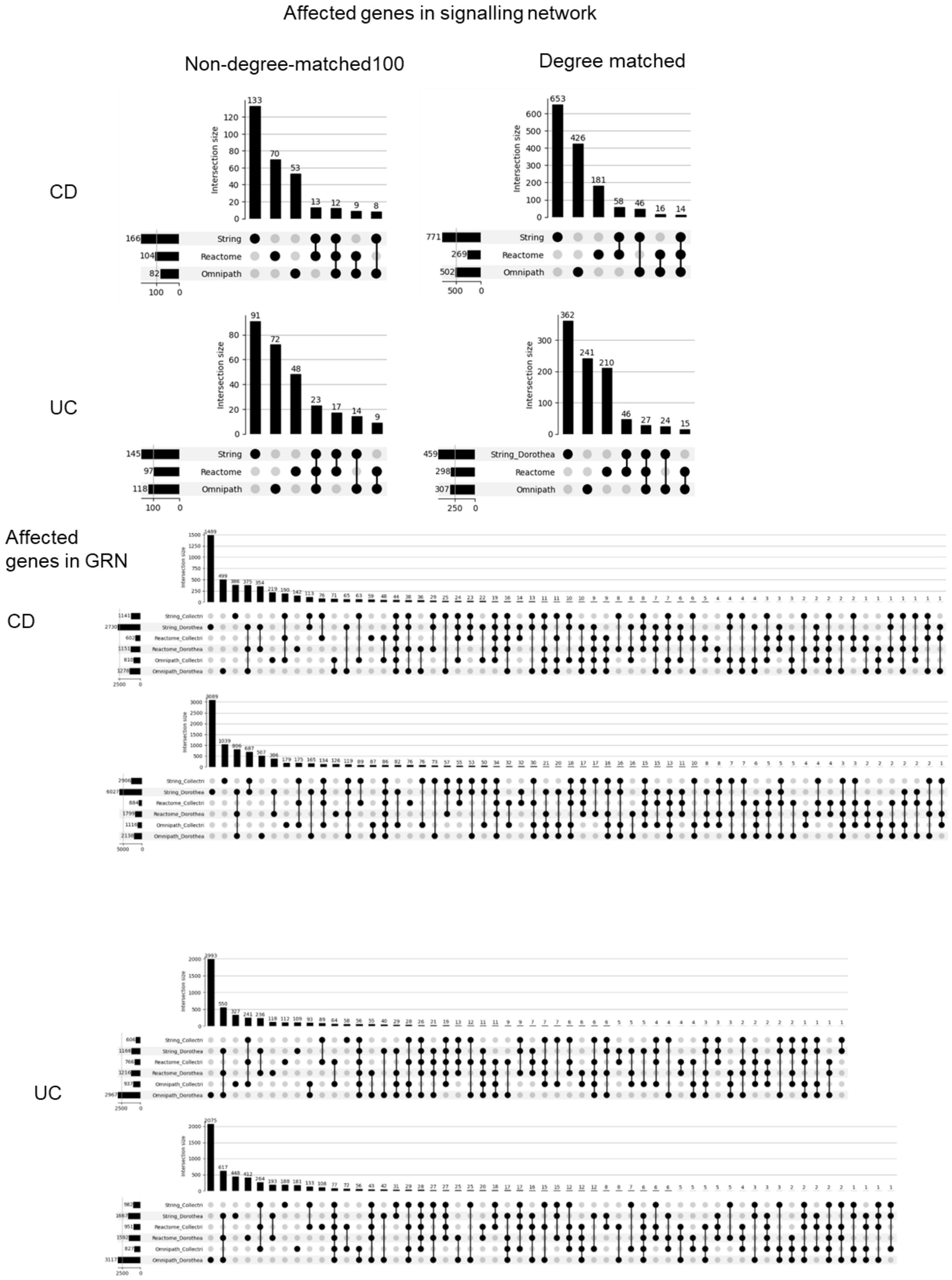

Appendix Figure S8. Comparing various network resources for network propagation

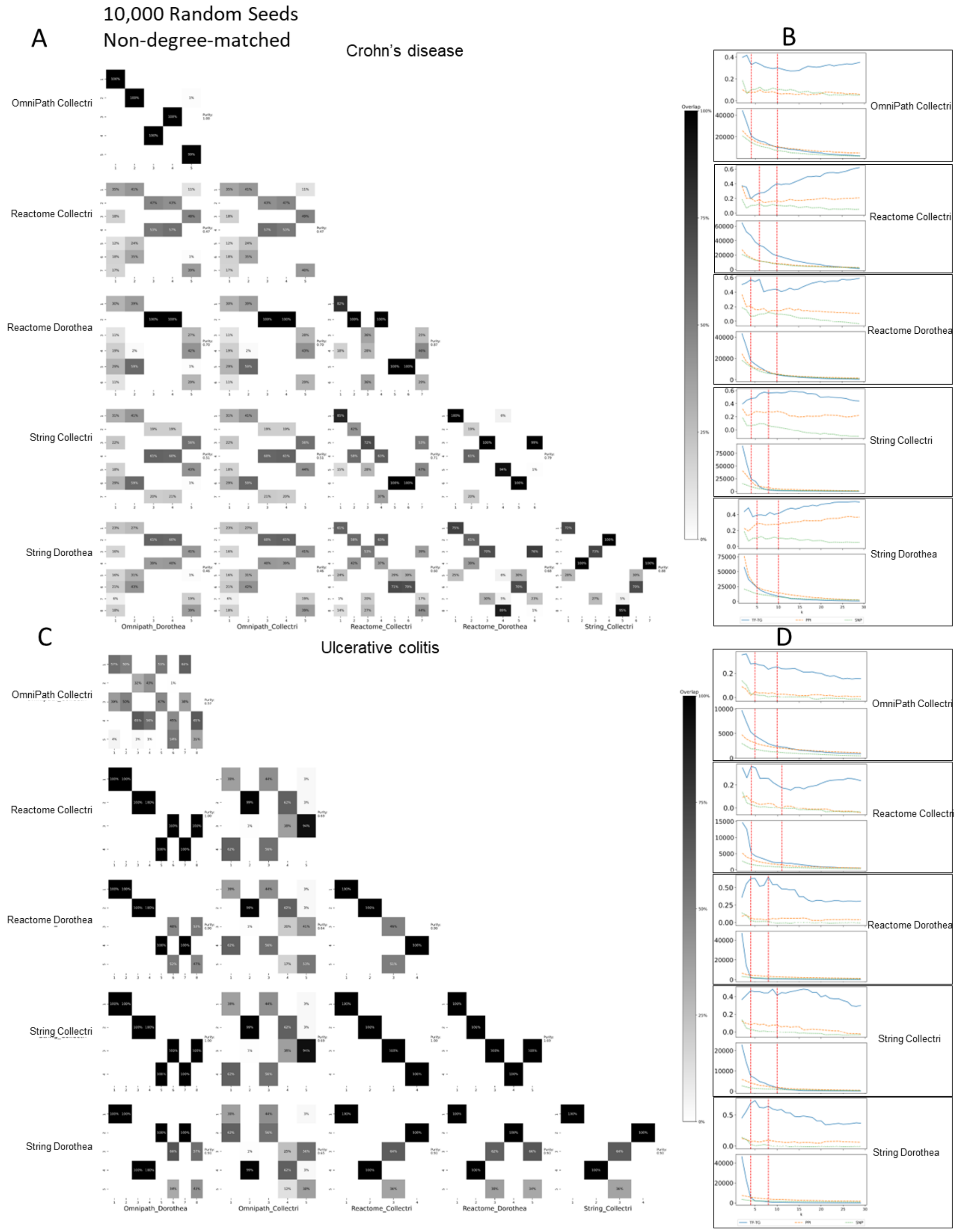
A
10,000 Random Seeds
Non-degree-matched
Crohn's disease
B
OmniPath Collectri
Reactome Collectri
Reactome Dorothea
String Collectri
String Dorothea
Omnipath_Dorothea
Omnipath_Collectri
Reactome_Collectri
Reactome_Dorothea
String_Collectri
C
Ulcerative colitis
D
OmniPath Collectri
Reactome Collectri
Reactome Dorothea
String Collectri
String Dorothea
Omnipath_Dorothea
Omnipath_Collectri
Reactome_Collectri
Reactome_Dorothea
String_Collectri

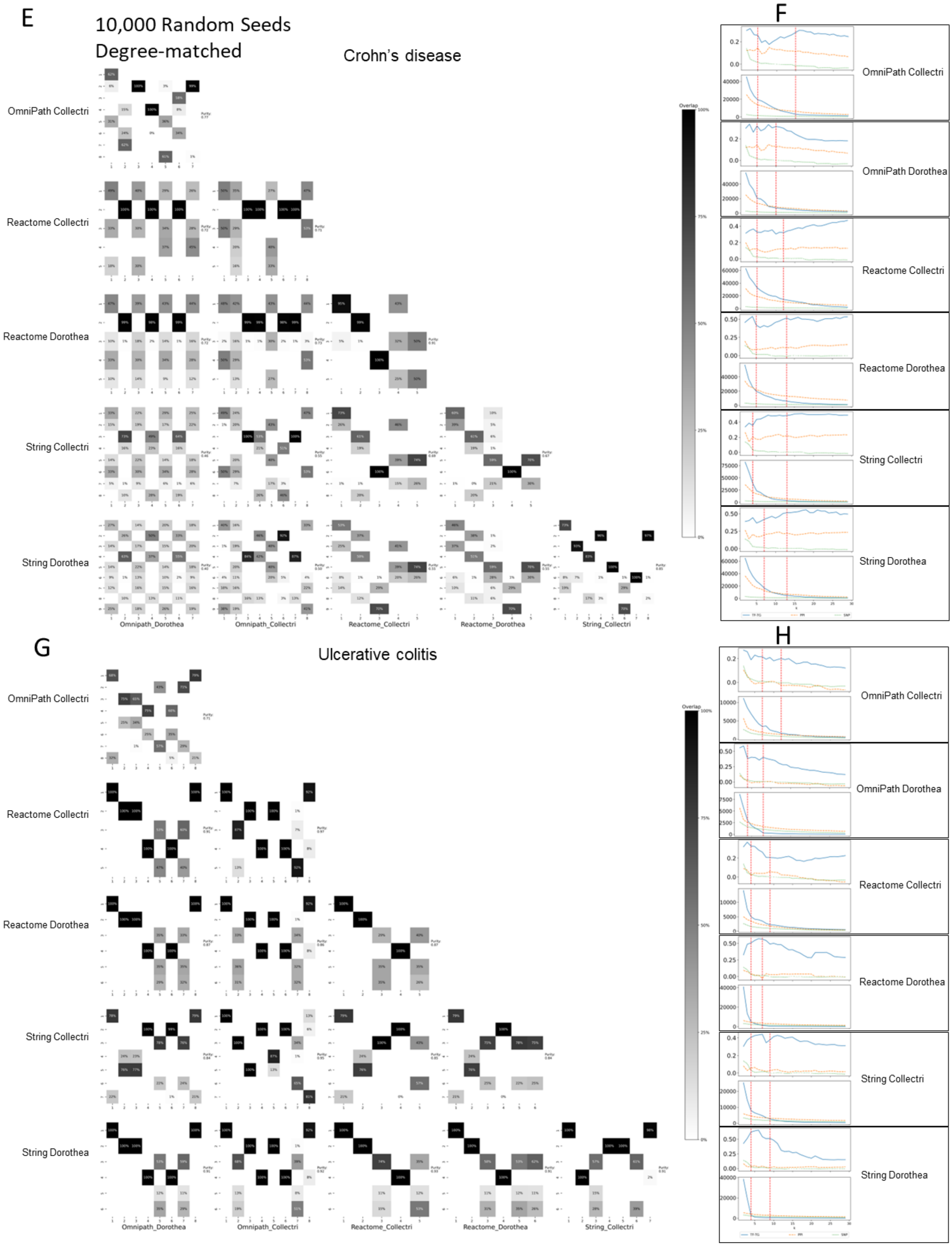

Appendix Figure S9. Patient clusters are independent of the used network in ulcerative colitis but have network-specific outcomes in Crohn's disease

A, B Crohn's disease non-degree matched; B,C Ulcerative colitis non-degree matched; E, F Crohn's disease degree-matched; F, G ulcerative colitis degree-matched; A,C,E,G Similarity of clustering outcomes using 3 different signalling networks (OmniPath, Reactome and

STRING) and 2 different regulatory networks (DorothEA and CollecTRI). The matrix values indicate the % of patients from the clusters in the x axis that are in the clusters in the y axis. Each matrix has a purity score which tells how similar the two clustering are. B, D, F, H Determining the number of patient clusters using silhouette score (inter-cluster distance) and inertia (inner-cluster distance). In each network pair, the regulatory network has the most different clusters. If multiple peaks were on the figure the first was depicted.

A) Crohn's Disease

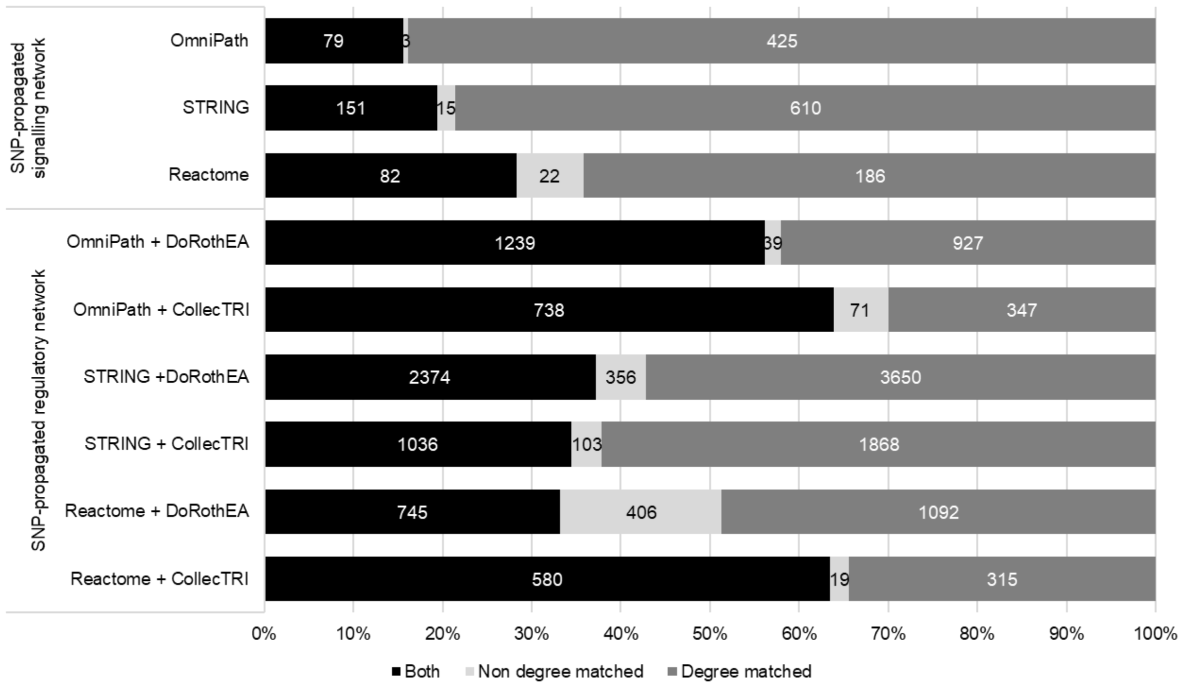

B) Ulcerative colits

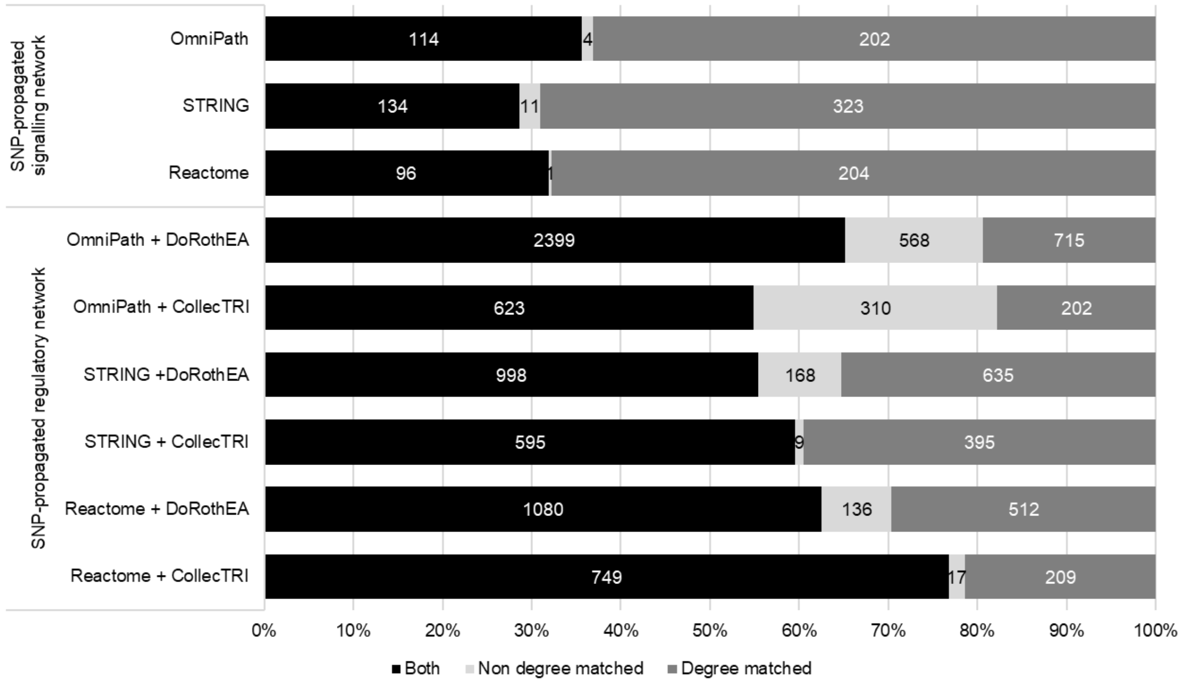

Appendix Figure S10. Similarity between degree-matched and non-degree-matched controls at signalling and regulatory layers using various network resources. A) Crohn's disease B) Ulcerative colitis.

Note that not many non-degree-matched SNP-propagated proteins are in the signalling networks.

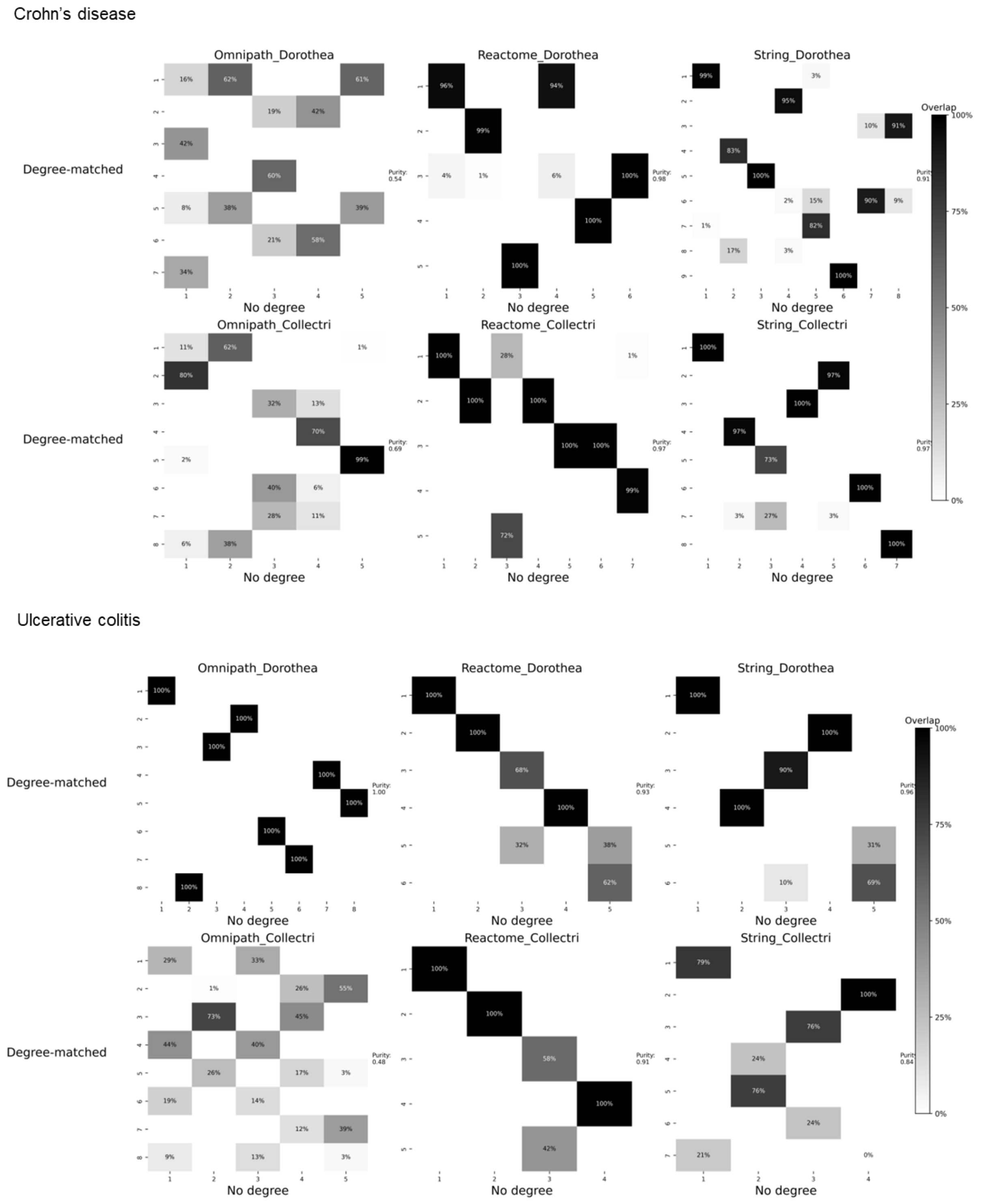

Appendix Figure S11. Using degree-matched algorithm compared to non-degree-matched algorithm results in similar clustering in ulcerative colitis but different outcomes in Crohn's disease.

A) Crohn's disease B) ulcerative colitis. The matrix values indicate the % of patients from the clusters in the x axis that are in the clusters in the y axis. Note that when using the OmniPath and DoRothEA networks in UC, the clustering is independent of whether degree-matched or independent randomisation is used.
