# Supplementary material for: Decoding non-coding SNPs: systems genomics modelling dissects the heterogeneity of IBD
Source: Mol Syst Biol. 2025 Nov 26;22(2):259–80. doi: 10.1038/s44320-025-00169-3 (PMC12864814; doi:10.1038/s44320-025-00169-3)
Supplement: Supplementary file 9 — Source data Fig. 1 [file 44320_2025_169_MOESM9_ESM.zip › Figure1.pptx]

## Slide 1
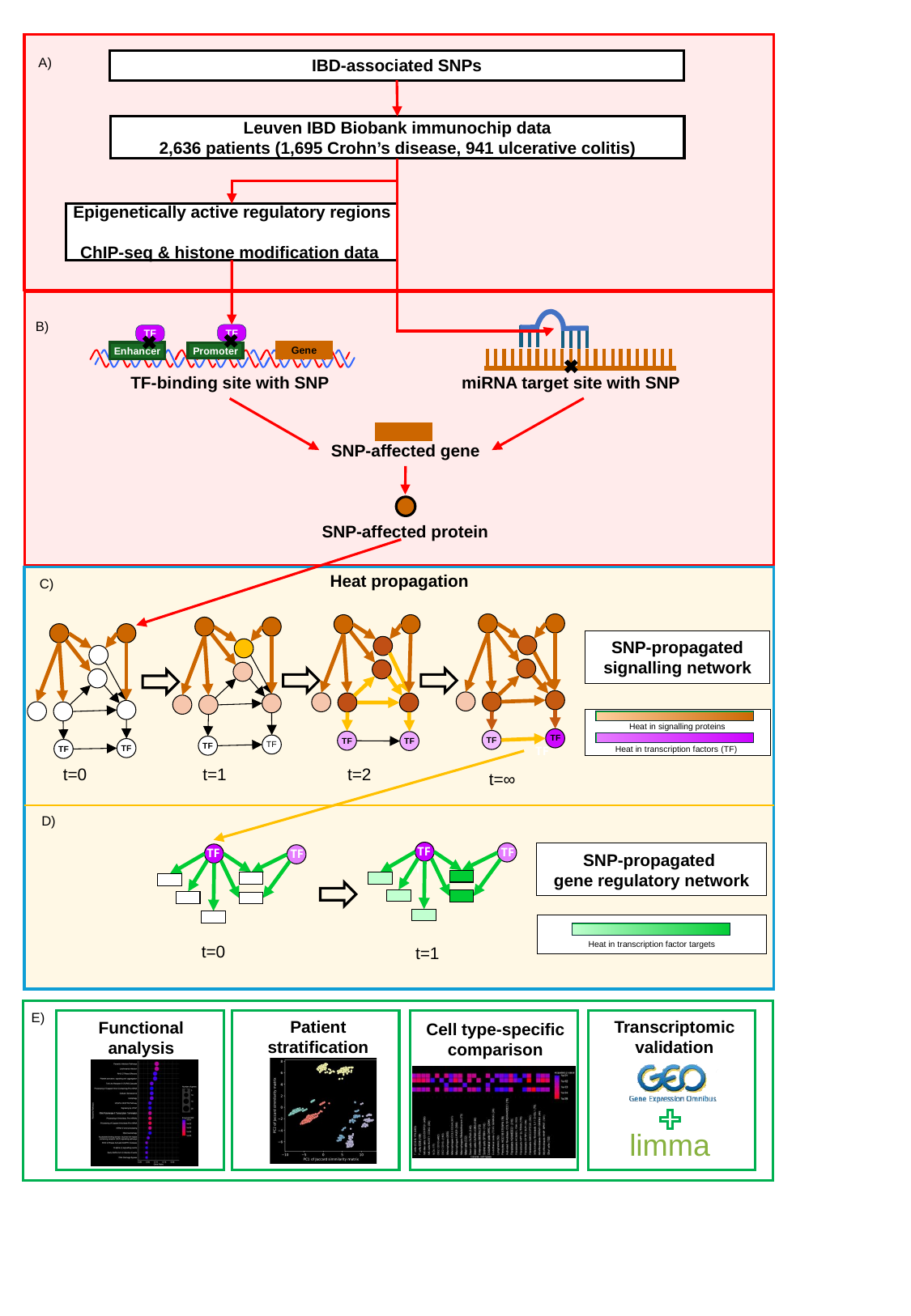

A)
IBD-associated SNPs
Leuven IBD Biobank immunochip data
2,636 patients (1,695 Crohn’s disease, 941 ulcerative colitis)
Epigenetically active regulatory regions
ChIP-seq & histone modification data
B)
TF
TF
Enhancer
Gene
Promoter
miRNA target site with SNP
TF-binding site with SNP
SNP-affected gene
SNP-affected protein
Heat propagation
C)
TF
TF
SNP-propagated signalling network
Heat in signalling proteins
Heat in transcription factors (TF)
TF
TF
TF
TF
TF
TF
TF
t=0
t=1
t=2
t=∞
D)
TF
TF
TF
TF
SNP-propagated
gene regulatory network
TF
Heat in transcription factor targets
t=0
t=1
E)
Transcriptomic validation
Patient stratification
Functional analysis
Cell type-specific comparison
limma
