# Supplementary material for: Decoding non-coding SNPs: systems genomics modelling dissects the heterogeneity of IBD
Source: Mol Syst Biol. 2025 Nov 26;22(2):259–80. doi: 10.1038/s44320-025-00169-3 (PMC12864814; doi:10.1038/s44320-025-00169-3)
Supplement: Supplementary file 10 — Source data Fig. 2 [file 44320_2025_169_MOESM10_ESM.zip › Figure2c/Figure2c.nb.html]

R Notebook Figure 2c


Code 

- Show All Code
- Hide All Code
- Download Rmd

# R Notebook Figure 2c

Please set work directory to the notebook folder.

1. Remove anything left in the datafiles


```
rm(list=ls())
```


If necesearry please install the following packages:2 if
(!requireNamespace(“BiocManager”, quietly = TRUE))
install.packages(“BiocManager”) BiocManager::install(“clusterProfiler”)
BiocManager::install(“ReactomePA”) BiocManager::install(“rrvgo”)
BiocManager::install(“enrichplot”) BiocManager::install(“msigdbr”)
BiocManager::install(“org.Hs.eg.db”)
BiocManager::install(“AnnotationDbi”) install.packages(“ggplot2”)
install.packages(“glue”)

```
Installing MULEA

<!-- rnb-text-end -->


<!-- rnb-chunk-begin -->


<!-- rnb-output-begin eyJkYXRhIjoiXG48IS0tIHJuYi1zb3VyY2UtYmVnaW4gZXlKa1lYUmhJam9pWUdCZ2NseHVTVzV6ZEdGc2JHbHVaeUIwYUdVZ1FtbHZZMDFoYm1GblpYSWdjR0ZqYTJGblpTQnBaaUJ1WldWa1pXUmNiaU5KYm5OMFlXeHNhVzVuSUhSb1pTQm1aM05sWVNCd1lXTnJZV2RsSUhkcGRHZ2dkR2hsSUVKcGIyTk5ZVzVoWjJWeVhHNUNhVzlqVFdGdVlXZGxjam82YVc1emRHRnNiQ2hjSW1abmMyVmhYQ0lwWEc1cGJuTjBZV3hzTG5CaFkydGhaMlZ6S0Z3aWJYVnNaV0ZjSWlsY2JtQmdZQ0o5IC0tPlxuXG5gYGByXG5JbnN0YWxsaW5nIHRoZSBCaW9jTWFuYWdlciBwYWNrYWdlIGlmIG5lZWRlZFxuI0luc3RhbGxpbmcgdGhlIGZnc2VhIHBhY2thZ2Ugd2l0aCB0aGUgQmlvY01hbmFnZXJcbkJpb2NNYW5hZ2VyOjppbnN0YWxsKFwiZmdzZWFcIilcbmluc3RhbGwucGFja2FnZXMoXCJtdWxlYVwiKVxuYGBgXG5cbjwhLS0gcm5iLXNvdXJjZS1lbmQgLS0+XG4ifQ== -->


<!-- rnb-source-begin eyJkYXRhIjoiYGBgclxuSW5zdGFsbGluZyB0aGUgQmlvY01hbmFnZXIgcGFja2FnZSBpZiBuZWVkZWRcbiNJbnN0YWxsaW5nIHRoZSBmZ3NlYSBwYWNrYWdlIHdpdGggdGhlIEJpb2NNYW5hZ2VyXG5CaW9jTWFuYWdlcjo6aW5zdGFsbChcImZnc2VhXCIpXG5pbnN0YWxsLnBhY2thZ2VzKFwibXVsZWFcIilcbmBgYCJ9 -->

```r
Installing the BiocManager package if needed
#Installing the fgsea package with the BiocManager
BiocManager::install("fgsea")
install.packages("mulea")
```


2. Reading in necesearry packages


```
library(clusterProfiler)
library(ReactomePA)
library(rrvgo)
library(enrichplot)
library(ggplot2)
library(msigdbr)
organism ="org.Hs.eg.db"
library(organism, character.only = TRUE)
library(glue)
library(mulea)
library(tidyverse)
library(AnnotationDbi)
library(scales)
```


3. Reading in data files


```
outcome_cd <- read.csv("cd_only_ppi10rnd.txt", sep="\t", row.names = 1)
head(outcome_cd)
selected_outcome_cd <- outcome_cd[outcome_cd$Z_Count>0,]
```


Reactome gene annotation file


```
reactome_ontology <- read_gmt("ReactomePathways.gmt")
```


```
reactome_ontology <- reactome_ontology %>% 
rename(ontology_id = "ontology_id",
    ontology_name = "ontology_name",
    list_of_values = "list_of_values")
head(reactome_ontology)
```


Filtering onytology for minimum 5 and maximmum 500 elments -
excluding really large and really small patheways. We can change theese
settings later.


```
reactome_ontology_filtered <- filter_ontology(gmt = reactome_ontology,
                                        min_nr_of_elements = 5,
                                        max_nr_of_elements = 500)
```


OmniPath newtwork file


```
op <- read.csv("OmniPath_27_10_2021_directed.ncol", sep=" ", header = FALSE, row.names = NULL)
head(op)
```


```
gene_ontology_filtered <- filter_ontology(gmt = gene_ontology,
                                        min_nr_of_elements = 5,
                                        max_nr_of_elements = 500)
```


```
bg <- unique(c(op$V1,op$V2))
```


# Translating genes in the background and outcome data to gene symbols

# Translate background genes


```
bg_symbols <- mapIds(org.Hs.eg.db, keys = bg, column = "SYMBOL", keytype = "UNIPROT", multiVals = "first")
```


5. Enrichmnet and creating Figure 2c


```
commonly_afffected_genes_cd <- selected_outcome_cd[selected_outcome_cd$Z_Count>100,]
```


```
dim(commonly_afffected_genes_cd)
```


```
commonly_afffected_cd_genes_symbols <- mapIds(org.Hs.eg.db, keys = row.names(commonly_afffected_genes_cd), 
column = "SYMBOL", keytype = "UNIPROT", multiVals = "first")
```


```
ora_model <- ora(gmt = reactome_ontology_filtered, 
                 # Test set variable
                 element_names = commonly_afffected_cd_genes_symbols, 
                 # Background set variable
                 background_element_names = bg_symbols, 
                 # p-value adjustment method
                 p_value_adjustment_method = "eFDR", 
                 # Number of permutations
                 number_of_permutations = 10000,
                 # Number of processor threads to use
                 nthreads = 2, 
                 # Setting a random seed for reproducibility
                 random_seed = 42) 

reactome_results_cd_ppi <- run_test(ora_model)
```


```
reactome_results_cd_ppi$GeneRatio <- reactome_results_cd_ppi$nr_common_with_tested_elements / length(commonly_afffected_cd_genes_symbols)
sig_results_cd_ppi <- reactome_results_cd_ppi %>%
    # Rows where the eFDR < 0.05
    filter(eFDR < 0.1) %>%
    # Arrange the rows by the gene ratio for plotting
    arrange(desc(GeneRatio))
sig_results_cd_ppi$ontology_id <- factor(sig_results_cd_ppi$ontology_id,
levels = sig_results_cd_ppi[order(sig_results_cd_ppi$GeneRatio, decreasing = FALSE), "ontology_id"])
sig_results_cd_ppi
```


```
write.csv(reactome_results_cd_ppi, glue("Reactome_CD_PPI_100_cutoff_ora_results.csv"), row.names = FALSE)
```


Label formatting


```
sig_results_cd_ppi$formed_id <- gsub("_", " ", sig_results_cd_ppi$ontology_id)
sig_results_cd_ppi$formed_id <- factor(sig_results_cd_ppi$formed_id,
                                    levels = sig_results_cd_ppi[order(sig_results_cd_ppi$GeneRatio,
                                    decreasing = FALSE), "formed_id"])
```


Adding a small amount for the fDR calcuation based on the MULEA
calcautions.


```
sig_results_cd_ppi$eFDR = sig_results_cd_ppi$eFDR +10^-12
```


```
dotplot_reactome_cd_ppi <- ggplot(sig_results_cd_ppi[1:20,], aes(x=GeneRatio, y=formed_id)) +
    geom_point(aes(size=nr_common_with_tested_elements, colour=eFDR)) +
    scale_y_discrete(labels = label_wrap(50)) + 
    scale_color_continuous(low="red", high="blue", 
                           limits=c(10^-5, 0.1),
                           trans = "log10", name="Emprical FDR")
                

dotplot_reactome_cd_ppi <- dotplot_reactome_cd_ppi +
    scale_size(limits = c(2,20), range = c(2,15), name = "Number of genes") + 
    xlim(0, 0.23) +
    xlab("Gene Ratio") +
    ylab("Reactome pathways") +
    theme_light() +
    theme(rect=element_rect(fill="white")) +
    theme(panel.background = element_rect(fill = "white")) +
    theme(plot.background = element_rect(fill = "white")) +
    theme(plot.background = element_rect(colour = "white"))+
    theme(axis.title.x = element_text(colour = "black", size = 10)) +
    theme(axis.title.y = element_text(colour = "black", size = 10)) +
    theme(axis.text = element_text(color= "black", size = 10)) +
    theme(axis.line = element_line(color = "black")) +
    theme(legend.background = element_rect(fill ="white")) +
    theme(legend.text = element_text(color="black", size = 10)) +
    theme(legend.title = element_text(color= "black", size = 10))
```


```
dotplot_reactome_cd_ppi
```


```
png("Fig3c.png",width=8, height=8, units="in", res=600)
dotplot_reactome_cd_ppi
dev.off()
```

LS0tDQp0aXRsZTogIlIgTm90ZWJvb2sgRmlndXJlIDJjIg0Kb3V0cHV0OiBodG1sX25vdGVib29rDQotLS0NCg0KUGxlYXNlIHNldCB3b3JrIGRpcmVjdG9yeSB0byB0aGUgbm90ZWJvb2sgZm9sZGVyLg0KDQoxLiBSZW1vdmUgYW55dGhpbmcgbGVmdCBpbiB0aGUgZGF0YWZpbGVzDQpgYGB7cn0NCnJtKGxpc3Q9bHMoKSkNCmBgYA0KSWYgbmVjZXNlYXJyeSBwbGVhc2UgaW5zdGFsbCB0aGUgZm9sbG93aW5nIHBhY2thZ2VzOjINCmlmICghcmVxdWlyZU5hbWVzcGFjZSgiQmlvY01hbmFnZXIiLCBxdWlldGx5ID0gVFJVRSkpDQogICAgaW5zdGFsbC5wYWNrYWdlcygiQmlvY01hbmFnZXIiKQ0KQmlvY01hbmFnZXI6Omluc3RhbGwoImNsdXN0ZXJQcm9maWxlciIpDQpCaW9jTWFuYWdlcjo6aW5zdGFsbCgiUmVhY3RvbWVQQSIpIA0KQmlvY01hbmFnZXI6Omluc3RhbGwoInJydmdvIikNCkJpb2NNYW5hZ2VyOjppbnN0YWxsKCJlbnJpY2hwbG90IikNCkJpb2NNYW5hZ2VyOjppbnN0YWxsKCJtc2lnZGJyIikNCkJpb2NNYW5hZ2VyOjppbnN0YWxsKCJvcmcuSHMuZWcuZGIiKQ0KQmlvY01hbmFnZXI6Omluc3RhbGwoIkFubm90YXRpb25EYmkiKQ0KaW5zdGFsbC5wYWNrYWdlcygiZ2dwbG90MiIpDQppbnN0YWxsLnBhY2thZ2VzKCJnbHVlIikNCmBgYA0KSW5zdGFsbGluZyBNVUxFQQ0KYGBge3J9DQpJbnN0YWxsaW5nIHRoZSBCaW9jTWFuYWdlciBwYWNrYWdlIGlmIG5lZWRlZA0KI0luc3RhbGxpbmcgdGhlIGZnc2VhIHBhY2thZ2Ugd2l0aCB0aGUgQmlvY01hbmFnZXINCkJpb2NNYW5hZ2VyOjppbnN0YWxsKCJmZ3NlYSIpDQppbnN0YWxsLnBhY2thZ2VzKCJtdWxlYSIpDQpgYGANCjIuIFJlYWRpbmcgaW4gbmVjZXNlYXJyeSBwYWNrYWdlcw0KYGBge3J9DQpsaWJyYXJ5KGNsdXN0ZXJQcm9maWxlcikNCmxpYnJhcnkoUmVhY3RvbWVQQSkNCmxpYnJhcnkocnJ2Z28pDQpsaWJyYXJ5KGVucmljaHBsb3QpDQpsaWJyYXJ5KGdncGxvdDIpDQpsaWJyYXJ5KG1zaWdkYnIpDQpvcmdhbmlzbSA9Im9yZy5Icy5lZy5kYiINCmxpYnJhcnkob3JnYW5pc20sIGNoYXJhY3Rlci5vbmx5ID0gVFJVRSkNCmxpYnJhcnkoZ2x1ZSkNCmxpYnJhcnkobXVsZWEpDQpsaWJyYXJ5KHRpZHl2ZXJzZSkNCmxpYnJhcnkoQW5ub3RhdGlvbkRiaSkNCmxpYnJhcnkoc2NhbGVzKQ0KYGBgDQozLiBSZWFkaW5nIGluIGRhdGEgZmlsZXMNCmBgYHtyfQ0Kb3V0Y29tZV9jZCA8LSByZWFkLmNzdigiY2Rfb25seV9wcGkxMHJuZC50eHQiLCBzZXA9Ilx0Iiwgcm93Lm5hbWVzID0gMSkNCmhlYWQob3V0Y29tZV9jZCkNCnNlbGVjdGVkX291dGNvbWVfY2QgPC0gb3V0Y29tZV9jZFtvdXRjb21lX2NkJFpfQ291bnQ+MCxdIA0KYGBgDQpSZWFjdG9tZSBnZW5lIGFubm90YXRpb24gZmlsZQ0KYGBge3J9DQpyZWFjdG9tZV9vbnRvbG9neSA8LSByZWFkX2dtdCgiUmVhY3RvbWVQYXRod2F5cy5nbXQiKQ0KYGBgDQpgYGB7cn0NCnJlYWN0b21lX29udG9sb2d5IDwtIHJlYWN0b21lX29udG9sb2d5ICU+JSANCnJlbmFtZShvbnRvbG9neV9pZCA9ICJvbnRvbG9neV9pZCIsDQogICAgb250b2xvZ3lfbmFtZSA9ICJvbnRvbG9neV9uYW1lIiwNCiAgICBsaXN0X29mX3ZhbHVlcyA9ICJsaXN0X29mX3ZhbHVlcyIpDQpoZWFkKHJlYWN0b21lX29udG9sb2d5KSAgICAgIA0KYGBgDQpGaWx0ZXJpbmcgb255dG9sb2d5IGZvciBtaW5pbXVtIDUgYW5kIG1heGltbXVtIDUwMCBlbG1lbnRzIC0gZXhjbHVkaW5nIHJlYWxseSBsYXJnZSBhbmQgcmVhbGx5IHNtYWxsIHBhdGhld2F5cy4gV2UgY2FuIGNoYW5nZSB0aGVlc2Ugc2V0dGluZ3MgbGF0ZXIuDQpgYGB7cn0NCnJlYWN0b21lX29udG9sb2d5X2ZpbHRlcmVkIDwtIGZpbHRlcl9vbnRvbG9neShnbXQgPSByZWFjdG9tZV9vbnRvbG9neSwNCiAgICAgICAgICAgICAgICAgICAgICAgICAgICAgICAgICAgICAgICBtaW5fbnJfb2ZfZWxlbWVudHMgPSA1LA0KICAgICAgICAgICAgICAgICAgICAgICAgICAgICAgICAgICAgICAgIG1heF9ucl9vZl9lbGVtZW50cyA9IDUwMCkNCmBgYA0KT21uaVBhdGggbmV3dHdvcmsgZmlsZQ0KYGBge3J9DQpvcCA8LSByZWFkLmNzdigiT21uaVBhdGhfMjdfMTBfMjAyMV9kaXJlY3RlZC5uY29sIiwgc2VwPSIgIiwgaGVhZGVyID0gRkFMU0UsIHJvdy5uYW1lcyA9IE5VTEwpDQpoZWFkKG9wKQ0KYGBgDQpgYGB7cn0NCmdlbmVfb250b2xvZ3lfZmlsdGVyZWQgPC0gZmlsdGVyX29udG9sb2d5KGdtdCA9IGdlbmVfb250b2xvZ3ksDQogICAgICAgICAgICAgICAgICAgICAgICAgICAgICAgICAgICAgICAgbWluX25yX29mX2VsZW1lbnRzID0gNSwNCiAgICAgICAgICAgICAgICAgICAgICAgICAgICAgICAgICAgICAgICBtYXhfbnJfb2ZfZWxlbWVudHMgPSA1MDApDQpgYGANCmBgYHtyfQ0KYmcgPC0gdW5pcXVlKGMob3AkVjEsb3AkVjIpKQ0KYGBgDQoNCiMgVHJhbnNsYXRpbmcgZ2VuZXMgaW4gdGhlIGJhY2tncm91bmQgYW5kIG91dGNvbWUgZGF0YSB0byBnZW5lIHN5bWJvbHMNCg0KIyBUcmFuc2xhdGUgYmFja2dyb3VuZCBnZW5lcw0KYGBge3J9DQpiZ19zeW1ib2xzIDwtIG1hcElkcyhvcmcuSHMuZWcuZGIsIGtleXMgPSBiZywgY29sdW1uID0gIlNZTUJPTCIsIGtleXR5cGUgPSAiVU5JUFJPVCIsIG11bHRpVmFscyA9ICJmaXJzdCIpDQpgYGANCg0KNS4gRW5yaWNobW5ldCBhbmQgY3JlYXRpbmcgRmlndXJlIDJjDQpgYGB7cn0NCmNvbW1vbmx5X2FmZmZlY3RlZF9nZW5lc19jZCA8LSBzZWxlY3RlZF9vdXRjb21lX2NkW3NlbGVjdGVkX291dGNvbWVfY2QkWl9Db3VudD4xMDAsXSANCmBgYA0KYGBge3J9DQpkaW0oY29tbW9ubHlfYWZmZmVjdGVkX2dlbmVzX2NkKQ0KYGBgDQpgYGB7cn0NCmNvbW1vbmx5X2FmZmZlY3RlZF9jZF9nZW5lc19zeW1ib2xzIDwtIG1hcElkcyhvcmcuSHMuZWcuZGIsIGtleXMgPSByb3cubmFtZXMoY29tbW9ubHlfYWZmZmVjdGVkX2dlbmVzX2NkKSwgDQpjb2x1bW4gPSAiU1lNQk9MIiwga2V5dHlwZSA9ICJVTklQUk9UIiwgbXVsdGlWYWxzID0gImZpcnN0IikNCmBgYA0KDQpgYGB7cn0NCm9yYV9tb2RlbCA8LSBvcmEoZ210ID0gcmVhY3RvbWVfb250b2xvZ3lfZmlsdGVyZWQsIA0KICAgICAgICAgICAgICAgICAjIFRlc3Qgc2V0IHZhcmlhYmxlDQogICAgICAgICAgICAgICAgIGVsZW1lbnRfbmFtZXMgPSBjb21tb25seV9hZmZmZWN0ZWRfY2RfZ2VuZXNfc3ltYm9scywgDQogICAgICAgICAgICAgICAgICMgQmFja2dyb3VuZCBzZXQgdmFyaWFibGUNCiAgICAgICAgICAgICAgICAgYmFja2dyb3VuZF9lbGVtZW50X25hbWVzID0gYmdfc3ltYm9scywgDQogICAgICAgICAgICAgICAgICMgcC12YWx1ZSBhZGp1c3RtZW50IG1ldGhvZA0KICAgICAgICAgICAgICAgICBwX3ZhbHVlX2FkanVzdG1lbnRfbWV0aG9kID0gImVGRFIiLCANCiAgICAgICAgICAgICAgICAgIyBOdW1iZXIgb2YgcGVybXV0YXRpb25zDQogICAgICAgICAgICAgICAgIG51bWJlcl9vZl9wZXJtdXRhdGlvbnMgPSAxMDAwMCwNCiAgICAgICAgICAgICAgICAgIyBOdW1iZXIgb2YgcHJvY2Vzc29yIHRocmVhZHMgdG8gdXNlDQogICAgICAgICAgICAgICAgIG50aHJlYWRzID0gMiwgDQogICAgICAgICAgICAgICAgICMgU2V0dGluZyBhIHJhbmRvbSBzZWVkIGZvciByZXByb2R1Y2liaWxpdHkNCiAgICAgICAgICAgICAgICAgcmFuZG9tX3NlZWQgPSA0MikgDQoNCnJlYWN0b21lX3Jlc3VsdHNfY2RfcHBpIDwtIHJ1bl90ZXN0KG9yYV9tb2RlbCkNCmBgYA0KYGBge3J9DQpyZWFjdG9tZV9yZXN1bHRzX2NkX3BwaSRHZW5lUmF0aW8gPC0gcmVhY3RvbWVfcmVzdWx0c19jZF9wcGkkbnJfY29tbW9uX3dpdGhfdGVzdGVkX2VsZW1lbnRzIC8gbGVuZ3RoKGNvbW1vbmx5X2FmZmZlY3RlZF9jZF9nZW5lc19zeW1ib2xzKQ0Kc2lnX3Jlc3VsdHNfY2RfcHBpIDwtIHJlYWN0b21lX3Jlc3VsdHNfY2RfcHBpICU+JQ0KICAgICMgUm93cyB3aGVyZSB0aGUgZUZEUiA8IDAuMDUNCiAgICBmaWx0ZXIoZUZEUiA8IDAuMSkgJT4lDQogICAgIyBBcnJhbmdlIHRoZSByb3dzIGJ5IHRoZSBnZW5lIHJhdGlvIGZvciBwbG90dGluZw0KICAgIGFycmFuZ2UoZGVzYyhHZW5lUmF0aW8pKQ0Kc2lnX3Jlc3VsdHNfY2RfcHBpJG9udG9sb2d5X2lkIDwtIGZhY3RvcihzaWdfcmVzdWx0c19jZF9wcGkkb250b2xvZ3lfaWQsDQpsZXZlbHMgPSBzaWdfcmVzdWx0c19jZF9wcGlbb3JkZXIoc2lnX3Jlc3VsdHNfY2RfcHBpJEdlbmVSYXRpbywgZGVjcmVhc2luZyA9IEZBTFNFKSwgIm9udG9sb2d5X2lkIl0pDQpzaWdfcmVzdWx0c19jZF9wcGkNCmBgYA0KYGBge3J9DQp3cml0ZS5jc3YocmVhY3RvbWVfcmVzdWx0c19jZF9wcGksIGdsdWUoIlJlYWN0b21lX0NEX1BQSV8xMDBfY3V0b2ZmX29yYV9yZXN1bHRzLmNzdiIpLCByb3cubmFtZXMgPSBGQUxTRSkNCmBgYA0KTGFiZWwgZm9ybWF0dGluZw0KYGBge3J9DQpzaWdfcmVzdWx0c19jZF9wcGkkZm9ybWVkX2lkIDwtIGdzdWIoIl8iLCAiICIsIHNpZ19yZXN1bHRzX2NkX3BwaSRvbnRvbG9neV9pZCkNCnNpZ19yZXN1bHRzX2NkX3BwaSRmb3JtZWRfaWQgPC0gZmFjdG9yKHNpZ19yZXN1bHRzX2NkX3BwaSRmb3JtZWRfaWQsDQogICAgICAgICAgICAgICAgICAgICAgICAgICAgICAgICAgICBsZXZlbHMgPSBzaWdfcmVzdWx0c19jZF9wcGlbb3JkZXIoc2lnX3Jlc3VsdHNfY2RfcHBpJEdlbmVSYXRpbywNCiAgICAgICAgICAgICAgICAgICAgICAgICAgICAgICAgICAgIGRlY3JlYXNpbmcgPSBGQUxTRSksICJmb3JtZWRfaWQiXSkNCg0KYGBgDQpBZGRpbmcgYSBzbWFsbCBhbW91bnQgZm9yIHRoZSBmRFIgY2FsY3VhdGlvbiBiYXNlZCBvbiB0aGUgTVVMRUEgY2FsY2F1dGlvbnMuDQpgYGB7cn0NCnNpZ19yZXN1bHRzX2NkX3BwaSRlRkRSID0gc2lnX3Jlc3VsdHNfY2RfcHBpJGVGRFIgKzEwXi0xMg0KYGBgDQpgYGB7cn0NCmRvdHBsb3RfcmVhY3RvbWVfY2RfcHBpIDwtIGdncGxvdChzaWdfcmVzdWx0c19jZF9wcGlbMToyMCxdLCBhZXMoeD1HZW5lUmF0aW8sIHk9Zm9ybWVkX2lkKSkgKw0KICAgIGdlb21fcG9pbnQoYWVzKHNpemU9bnJfY29tbW9uX3dpdGhfdGVzdGVkX2VsZW1lbnRzLCBjb2xvdXI9ZUZEUikpICsNCiAgICBzY2FsZV95X2Rpc2NyZXRlKGxhYmVscyA9IGxhYmVsX3dyYXAoNTApKSArIA0KICAgIHNjYWxlX2NvbG9yX2NvbnRpbnVvdXMobG93PSJyZWQiLCBoaWdoPSJibHVlIiwgDQogICAgICAgICAgICAgICAgICAgICAgICAgICBsaW1pdHM9YygxMF4tNSwgMC4xKSwNCiAgICAgICAgICAgICAgICAgICAgICAgICAgIHRyYW5zID0gImxvZzEwIiwgbmFtZT0iRW1wcmljYWwgRkRSIikNCiAgICAgICAgICAgICAgICANCg0KZG90cGxvdF9yZWFjdG9tZV9jZF9wcGkgPC0gZG90cGxvdF9yZWFjdG9tZV9jZF9wcGkgKw0KICAgIHNjYWxlX3NpemUobGltaXRzID0gYygyLDIwKSwgcmFuZ2UgPSBjKDIsMTUpLCBuYW1lID0gIk51bWJlciBvZiBnZW5lcyIpICsgDQogICAgeGxpbSgwLCAwLjIzKSArDQogICAgeGxhYigiR2VuZSBSYXRpbyIpICsNCiAgICB5bGFiKCJSZWFjdG9tZSBwYXRod2F5cyIpICsNCiAgICB0aGVtZV9saWdodCgpICsNCiAgICB0aGVtZShyZWN0PWVsZW1lbnRfcmVjdChmaWxsPSJ3aGl0ZSIpKSArDQogICAgdGhlbWUocGFuZWwuYmFja2dyb3VuZCA9IGVsZW1lbnRfcmVjdChmaWxsID0gIndoaXRlIikpICsNCiAgICB0aGVtZShwbG90LmJhY2tncm91bmQgPSBlbGVtZW50X3JlY3QoZmlsbCA9ICJ3aGl0ZSIpKSArDQogICAgdGhlbWUocGxvdC5iYWNrZ3JvdW5kID0gZWxlbWVudF9yZWN0KGNvbG91ciA9ICJ3aGl0ZSIpKSsNCiAgICB0aGVtZShheGlzLnRpdGxlLnggPSBlbGVtZW50X3RleHQoY29sb3VyID0gImJsYWNrIiwgc2l6ZSA9IDEwKSkgKw0KICAgIHRoZW1lKGF4aXMudGl0bGUueSA9IGVsZW1lbnRfdGV4dChjb2xvdXIgPSAiYmxhY2siLCBzaXplID0gMTApKSArDQogICAgdGhlbWUoYXhpcy50ZXh0ID0gZWxlbWVudF90ZXh0KGNvbG9yPSAiYmxhY2siLCBzaXplID0gMTApKSArDQogICAgdGhlbWUoYXhpcy5saW5lID0gZWxlbWVudF9saW5lKGNvbG9yID0gImJsYWNrIikpICsNCiAgICB0aGVtZShsZWdlbmQuYmFja2dyb3VuZCA9IGVsZW1lbnRfcmVjdChmaWxsID0id2hpdGUiKSkgKw0KICAgIHRoZW1lKGxlZ2VuZC50ZXh0ID0gZWxlbWVudF90ZXh0KGNvbG9yPSJibGFjayIsIHNpemUgPSAxMCkpICsNCiAgICB0aGVtZShsZWdlbmQudGl0bGUgPSBlbGVtZW50X3RleHQoY29sb3I9ICJibGFjayIsIHNpemUgPSAxMCkpIA0KYGBgDQpgYGB7cn0NCmRvdHBsb3RfcmVhY3RvbWVfY2RfcHBpDQpgYGANCmBgYHtyfQ0KcG5nKCJGaWczYy5wbmciLHdpZHRoPTgsIGhlaWdodD04LCB1bml0cz0iaW4iLCByZXM9NjAwKQ0KZG90cGxvdF9yZWFjdG9tZV9jZF9wcGkNCmRldi5vZmYoKQ0KYGBgDQo=
