# Supplementary material for: Decoding non-coding SNPs: systems genomics modelling dissects the heterogeneity of IBD
Source: Mol Syst Biol. 2025 Nov 26;22(2):259–80. doi: 10.1038/s44320-025-00169-3 (PMC12864814; doi:10.1038/s44320-025-00169-3)
Supplement: Supplementary file 10 — Source data Fig. 2 [file 44320_2025_169_MOESM10_ESM.zip › Figure2b/Figure2b.nb.html]

R Notebook Figure 2b


Code 

- Show All Code
- Hide All Code
- Download Rmd

# R Notebook Figure 2b

1. Remove anything left in the datafiles


```
rm(list=ls())
```


If necesearry please install the following packages:


```
if (!requireNamespace("BiocManager", quietly = TRUE))
    install.packages("BiocManager")
BiocManager::install("clusterProfiler")
BiocManager::install("ReactomePA") 
BiocManager::install("rrvgo")
BiocManager::install("enrichplot")
BiocManager::install("msigdbr")
BiocManager::install("org.Hs.eg.db")
BiocManager::install("AnnotationDbi")
install.packages("ggplot2")
install.packages("glue")
```


Installing MULEA


```
Installing the BiocManager package if needed
#Installing the fgsea package with the BiocManager
BiocManager::install("fgsea")
install.packages("mulea")
```


2. Reading in necesearry packages


```
library(clusterProfiler)
library(ReactomePA)
library(rrvgo)
library(enrichplot)
library(ggplot2)
library(msigdbr)
organism ="org.Hs.eg.db"
library(organism, character.only = TRUE)
library(glue)
library(mulea)
library(tidyverse)
library(AnnotationDbi)
library(scales)
```


3. Data input and prepearation for enrtichment analysis THe working
   directory to be ecpected to be the folder of the Rmd file

PPI network propagation output


```
outcome_cd <- read.csv("cd_only_ppi10rnd.txt", sep="\t", row.names = 1)
head(outcome_cd)
selected_outcome_cd <- outcome_cd[outcome_cd$Z_Count>0,]
```


4. Creating Figure 2/b


```
histogramplot_cd <- ggplot(selected_outcome_cd, aes(x=Z_Count)) + 
    geom_histogram(colour="white", fill="orange", binwidth = 50)+
    ylab("Number of individual proteins") + xlab("Number of patients") +
    geom_vline(aes(xintercept=100),
            color="black", linetype="dashed", size=1) + 
    scale_x_continuous(breaks=seq(0,1400,200)) + theme_light()
    theme(panel.background = element_rect(fill = "black")) +
    theme(plot.background = element_rect(fill = "white", colour = "white")) +
    theme(axis.title.x = element_text(colour = "black", size = 10)) +
    theme(axis.title.y = element_text(colour = "black", size = 10)) +
    theme(axis.text = element_text(color= "black", size = 10)) +
    theme(axis.line = element_line(color = "black")) +
    theme(panel.grid.minor = element_blank())
histogramplot_cd
```


```
png(file=glue("Figure_2b.png"),width=8, height=4, units="in", res=600)
histogramplot_cd
dev.off()
```


LS0tDQp0aXRsZTogIlIgTm90ZWJvb2sgRmlndXJlIDJiIg0Kb3V0cHV0OiBodG1sX25vdGVib29rDQotLS0NCjEuIFJlbW92ZSBhbnl0aGluZyBsZWZ0IGluIHRoZSBkYXRhZmlsZXMNCmBgYHtyfQ0Kcm0obGlzdD1scygpKQ0KYGBgDQpJZiBuZWNlc2VhcnJ5IHBsZWFzZSBpbnN0YWxsIHRoZSBmb2xsb3dpbmcgcGFja2FnZXM6DQpgYGB7cn0NCmlmICghcmVxdWlyZU5hbWVzcGFjZSgiQmlvY01hbmFnZXIiLCBxdWlldGx5ID0gVFJVRSkpDQogICAgaW5zdGFsbC5wYWNrYWdlcygiQmlvY01hbmFnZXIiKQ0KQmlvY01hbmFnZXI6Omluc3RhbGwoImNsdXN0ZXJQcm9maWxlciIpDQpCaW9jTWFuYWdlcjo6aW5zdGFsbCgiUmVhY3RvbWVQQSIpIA0KQmlvY01hbmFnZXI6Omluc3RhbGwoInJydmdvIikNCkJpb2NNYW5hZ2VyOjppbnN0YWxsKCJlbnJpY2hwbG90IikNCkJpb2NNYW5hZ2VyOjppbnN0YWxsKCJtc2lnZGJyIikNCkJpb2NNYW5hZ2VyOjppbnN0YWxsKCJvcmcuSHMuZWcuZGIiKQ0KQmlvY01hbmFnZXI6Omluc3RhbGwoIkFubm90YXRpb25EYmkiKQ0KaW5zdGFsbC5wYWNrYWdlcygiZ2dwbG90MiIpDQppbnN0YWxsLnBhY2thZ2VzKCJnbHVlIikNCmBgYA0KSW5zdGFsbGluZyBNVUxFQQ0KYGBge3J9DQpJbnN0YWxsaW5nIHRoZSBCaW9jTWFuYWdlciBwYWNrYWdlIGlmIG5lZWRlZA0KI0luc3RhbGxpbmcgdGhlIGZnc2VhIHBhY2thZ2Ugd2l0aCB0aGUgQmlvY01hbmFnZXINCkJpb2NNYW5hZ2VyOjppbnN0YWxsKCJmZ3NlYSIpDQppbnN0YWxsLnBhY2thZ2VzKCJtdWxlYSIpDQpgYGANCjIuIFJlYWRpbmcgaW4gbmVjZXNlYXJyeSBwYWNrYWdlcw0KYGBge3J9DQpsaWJyYXJ5KGNsdXN0ZXJQcm9maWxlcikNCmxpYnJhcnkoUmVhY3RvbWVQQSkNCmxpYnJhcnkocnJ2Z28pDQpsaWJyYXJ5KGVucmljaHBsb3QpDQpsaWJyYXJ5KGdncGxvdDIpDQpsaWJyYXJ5KG1zaWdkYnIpDQpvcmdhbmlzbSA9Im9yZy5Icy5lZy5kYiINCmxpYnJhcnkob3JnYW5pc20sIGNoYXJhY3Rlci5vbmx5ID0gVFJVRSkNCmxpYnJhcnkoZ2x1ZSkNCmxpYnJhcnkobXVsZWEpDQpsaWJyYXJ5KHRpZHl2ZXJzZSkNCmxpYnJhcnkoQW5ub3RhdGlvbkRiaSkNCmxpYnJhcnkoc2NhbGVzKQ0KYGBgDQozLiBEYXRhIGlucHV0IGFuZCBwcmVwZWFyYXRpb24gZm9yIGVucnRpY2htZW50IGFuYWx5c2lzDQpUSGUgd29ya2luZyBkaXJlY3RvcnkgdG8gYmUgZWNwZWN0ZWQgdG8gYmUgdGhlIGZvbGRlciBvZiB0aGUgUm1kIGZpbGUNCg0KUFBJIG5ldHdvcmsgcHJvcGFnYXRpb24gb3V0cHV0DQpgYGB7cn0NCm91dGNvbWVfY2QgPC0gcmVhZC5jc3YoImNkX29ubHlfcHBpMTBybmQudHh0Iiwgc2VwPSJcdCIsIHJvdy5uYW1lcyA9IDEpDQpoZWFkKG91dGNvbWVfY2QpDQpzZWxlY3RlZF9vdXRjb21lX2NkIDwtIG91dGNvbWVfY2Rbb3V0Y29tZV9jZCRaX0NvdW50PjAsXSANCmBgYA0KNC4gQ3JlYXRpbmcgRmlndXJlIDIvYg0KYGBge3J9DQpoaXN0b2dyYW1wbG90X2NkIDwtIGdncGxvdChzZWxlY3RlZF9vdXRjb21lX2NkLCBhZXMoeD1aX0NvdW50KSkgKyANCiAgICBnZW9tX2hpc3RvZ3JhbShjb2xvdXI9IndoaXRlIiwgZmlsbD0ib3JhbmdlIiwgYmlud2lkdGggPSA1MCkrDQogICAgeWxhYigiTnVtYmVyIG9mIGluZGl2aWR1YWwgcHJvdGVpbnMiKSArIHhsYWIoIk51bWJlciBvZiBwYXRpZW50cyIpICsNCiAgICBnZW9tX3ZsaW5lKGFlcyh4aW50ZXJjZXB0PTEwMCksDQogICAgICAgICAgICBjb2xvcj0iYmxhY2siLCBsaW5ldHlwZT0iZGFzaGVkIiwgc2l6ZT0xKSArIA0KICAgIHNjYWxlX3hfY29udGludW91cyhicmVha3M9c2VxKDAsMTQwMCwyMDApKSArIHRoZW1lX2xpZ2h0KCkNCiAgICB0aGVtZShwYW5lbC5iYWNrZ3JvdW5kID0gZWxlbWVudF9yZWN0KGZpbGwgPSAiYmxhY2siKSkgKw0KICAgIHRoZW1lKHBsb3QuYmFja2dyb3VuZCA9IGVsZW1lbnRfcmVjdChmaWxsID0gIndoaXRlIiwgY29sb3VyID0gIndoaXRlIikpICsNCiAgICB0aGVtZShheGlzLnRpdGxlLnggPSBlbGVtZW50X3RleHQoY29sb3VyID0gImJsYWNrIiwgc2l6ZSA9IDEwKSkgKw0KICAgIHRoZW1lKGF4aXMudGl0bGUueSA9IGVsZW1lbnRfdGV4dChjb2xvdXIgPSAiYmxhY2siLCBzaXplID0gMTApKSArDQogICAgdGhlbWUoYXhpcy50ZXh0ID0gZWxlbWVudF90ZXh0KGNvbG9yPSAiYmxhY2siLCBzaXplID0gMTApKSArDQogICAgdGhlbWUoYXhpcy5saW5lID0gZWxlbWVudF9saW5lKGNvbG9yID0gImJsYWNrIikpICsNCiAgICB0aGVtZShwYW5lbC5ncmlkLm1pbm9yID0gZWxlbWVudF9ibGFuaygpKQ0KaGlzdG9ncmFtcGxvdF9jZA0KYGBgDQpgYGB7cn0NCnBuZyhmaWxlPWdsdWUoIkZpZ3VyZV8yYi5wbmciKSx3aWR0aD04LCBoZWlnaHQ9NCwgdW5pdHM9ImluIiwgcmVzPTYwMCkNCmhpc3RvZ3JhbXBsb3RfY2QNCmRldi5vZmYoKQ0KYGBgDQo=
