# Supplementary figures and images for: Decoding non-coding SNPs: systems genomics modelling dissects the heterogeneity of IBD
Source: Mol Syst Biol. 2025 Nov 26;22(2):259–80. doi: 10.1038/s44320-025-00169-3 (PMC12864814; doi:10.1038/s44320-025-00169-3)

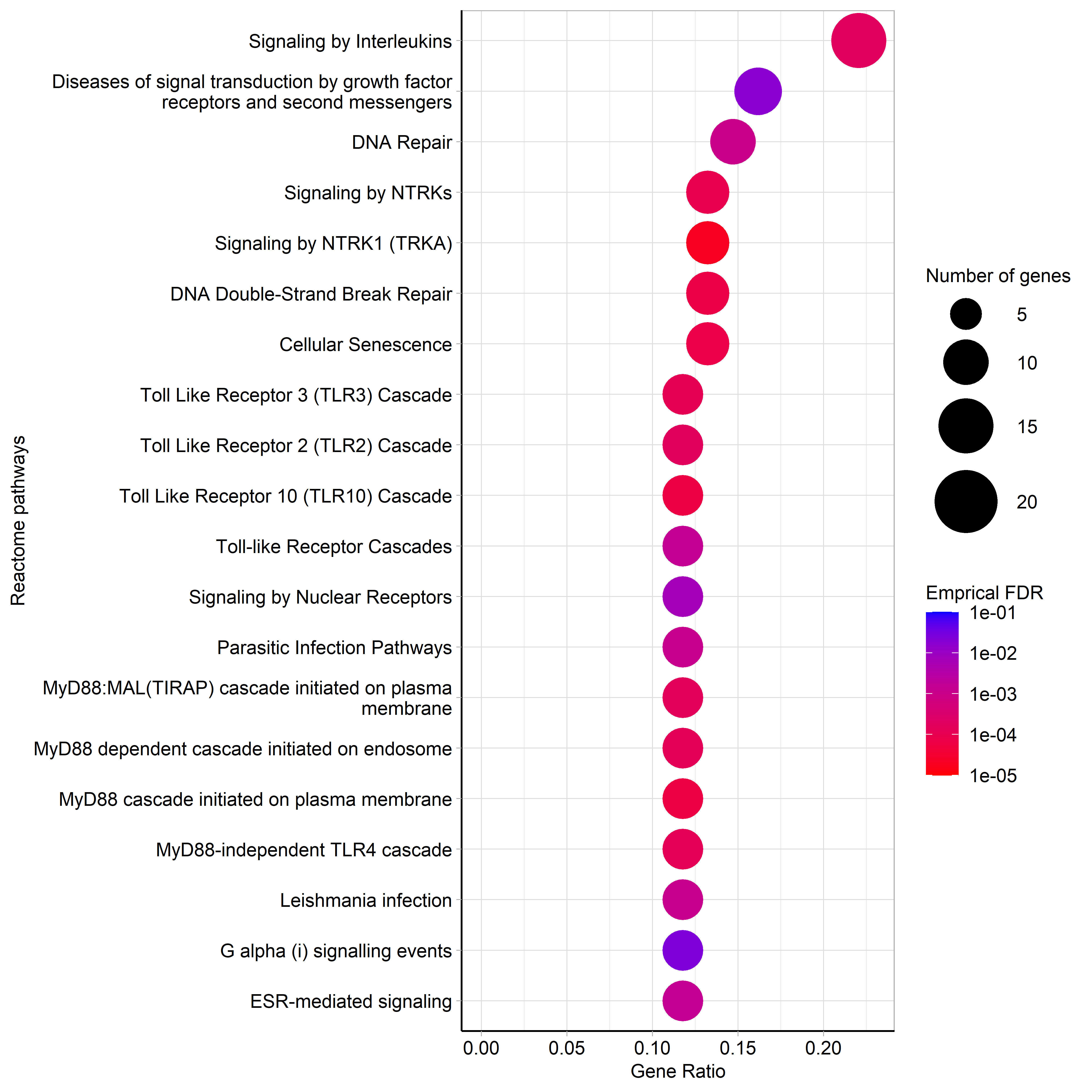

Supplement: Supplementary file 10 — Source data Fig. 2 [file 44320_2025_169_MOESM10_ESM.zip › Figure2c/Fig3c.png]

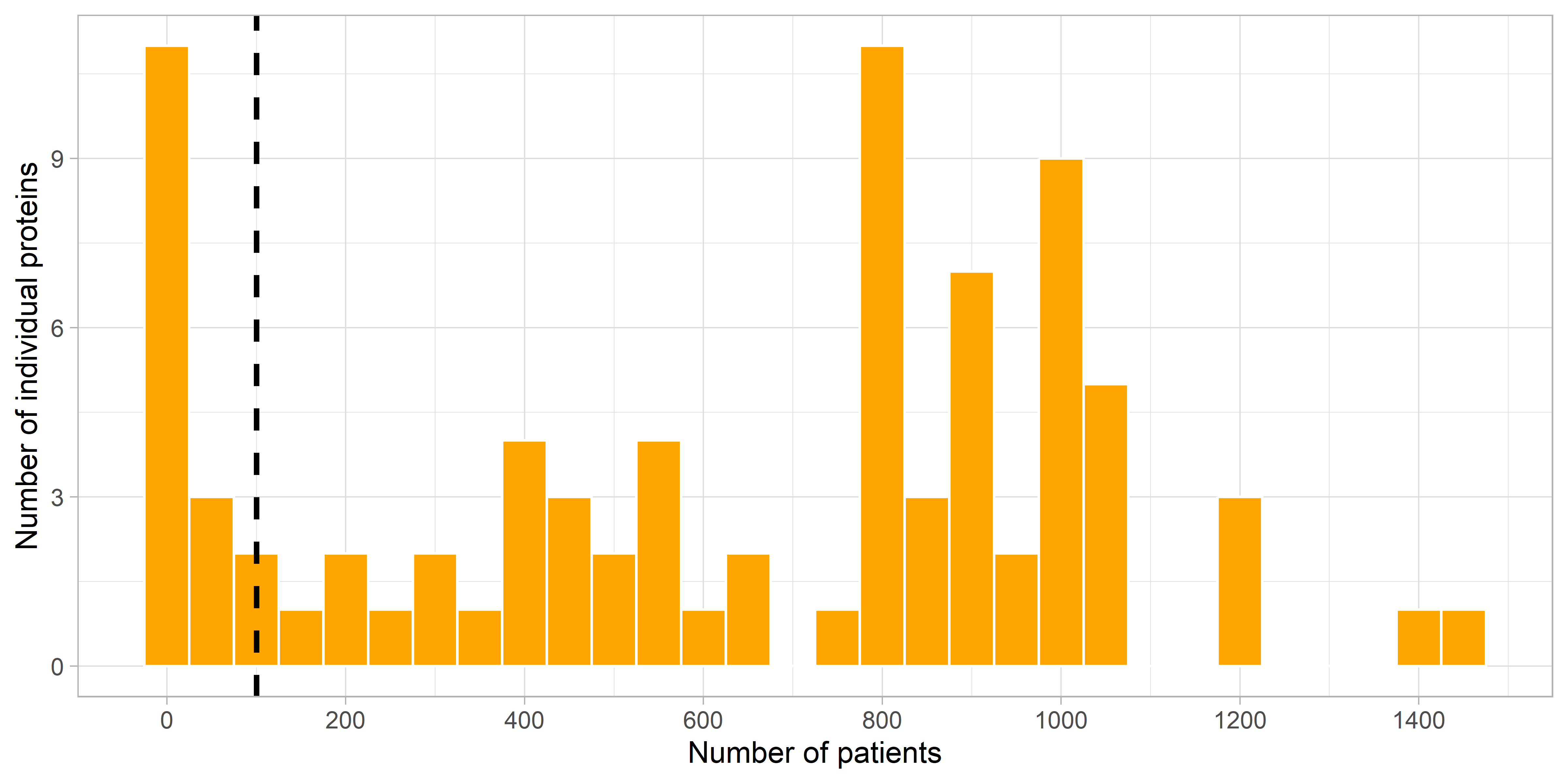

Supplement: Supplementary file 10 — Source data Fig. 2 [file 44320_2025_169_MOESM10_ESM.zip › Figure2b/Figure_2b.png]

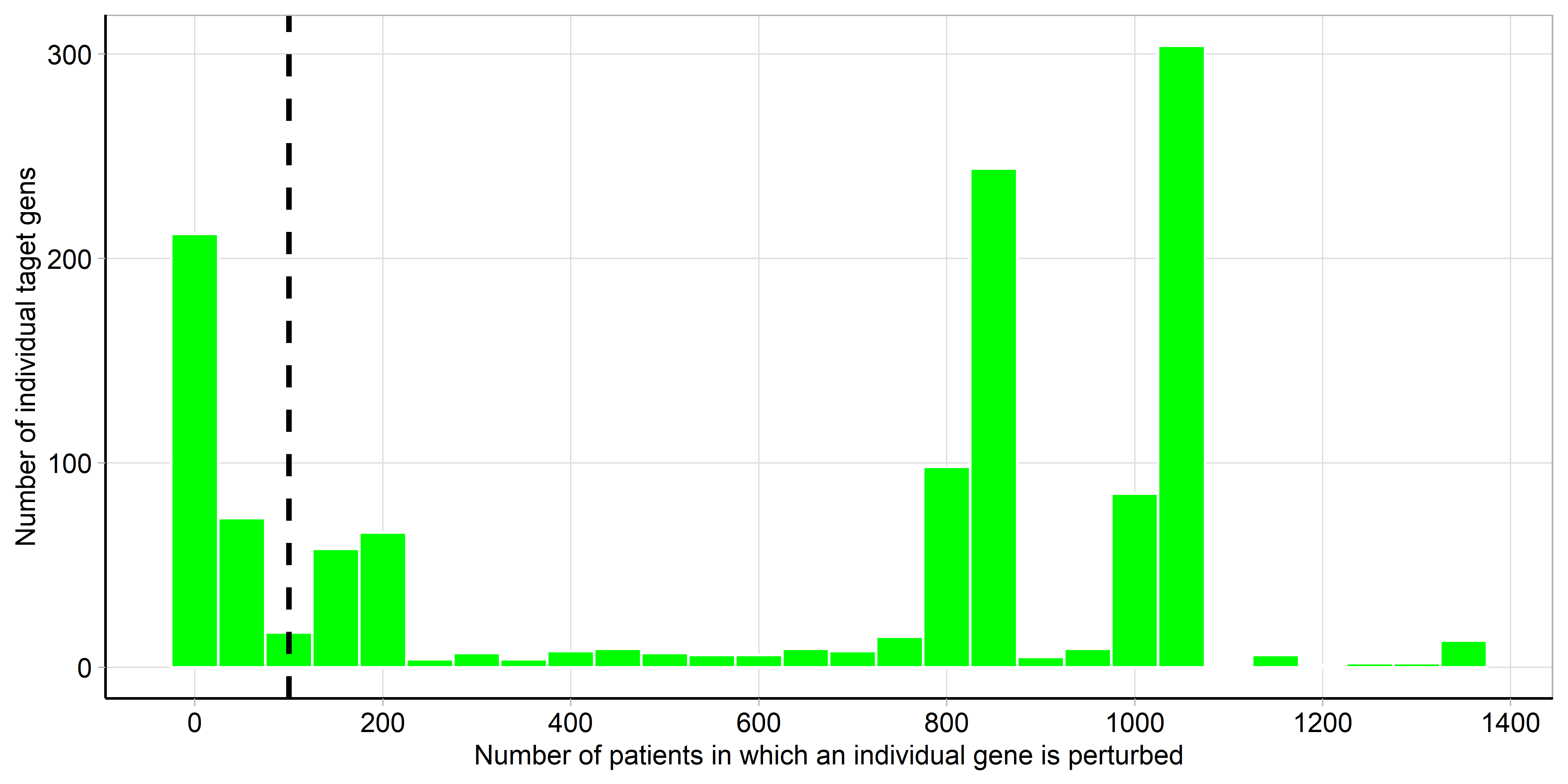

Supplement: Supplementary file 11 — Source data Fig. 3 [file 44320_2025_169_MOESM11_ESM.zip › Figure3_b/Figure3b.png]

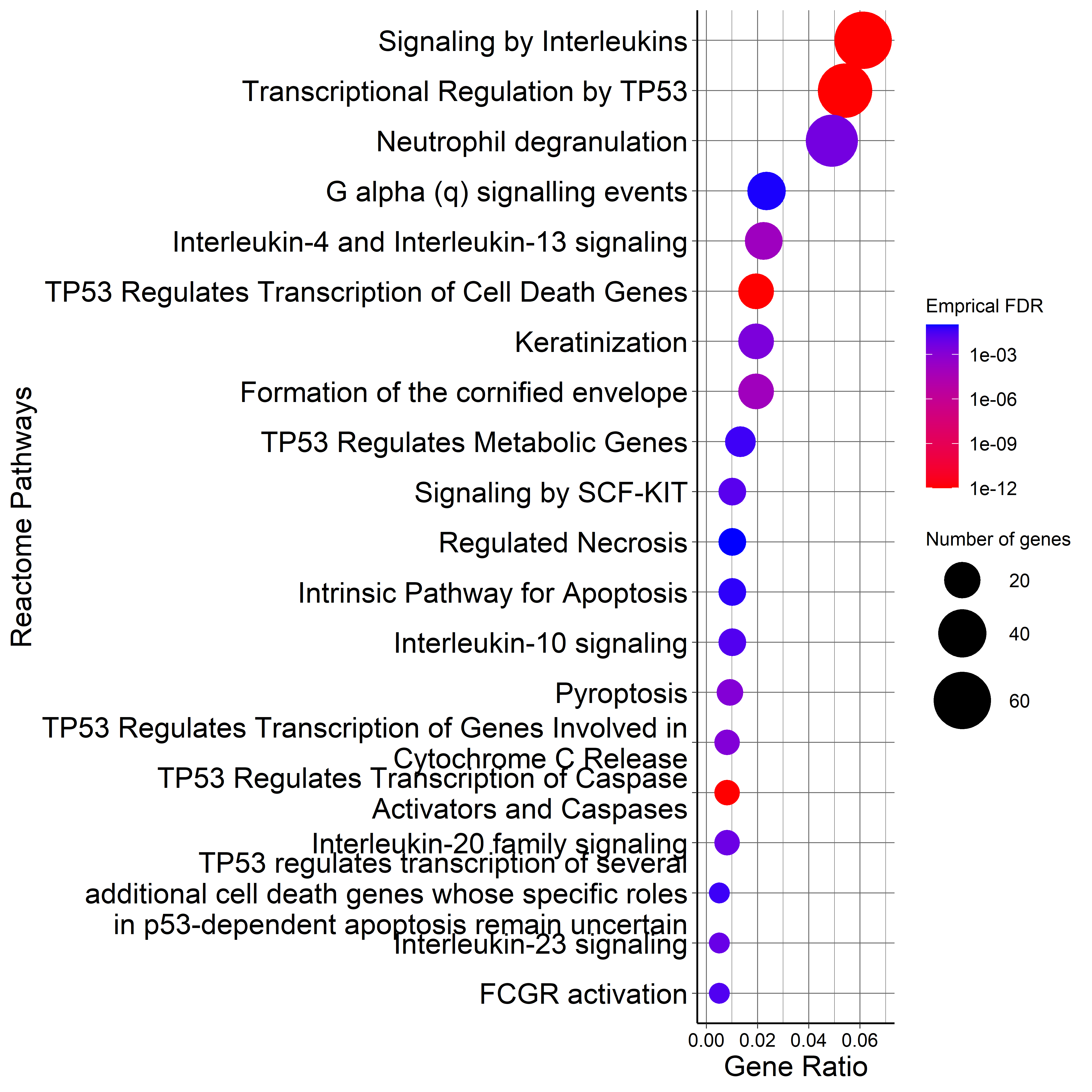

Supplement: Supplementary file 11 — Source data Fig. 3 [file 44320_2025_169_MOESM11_ESM.zip › Figure3_c/Figure3c]

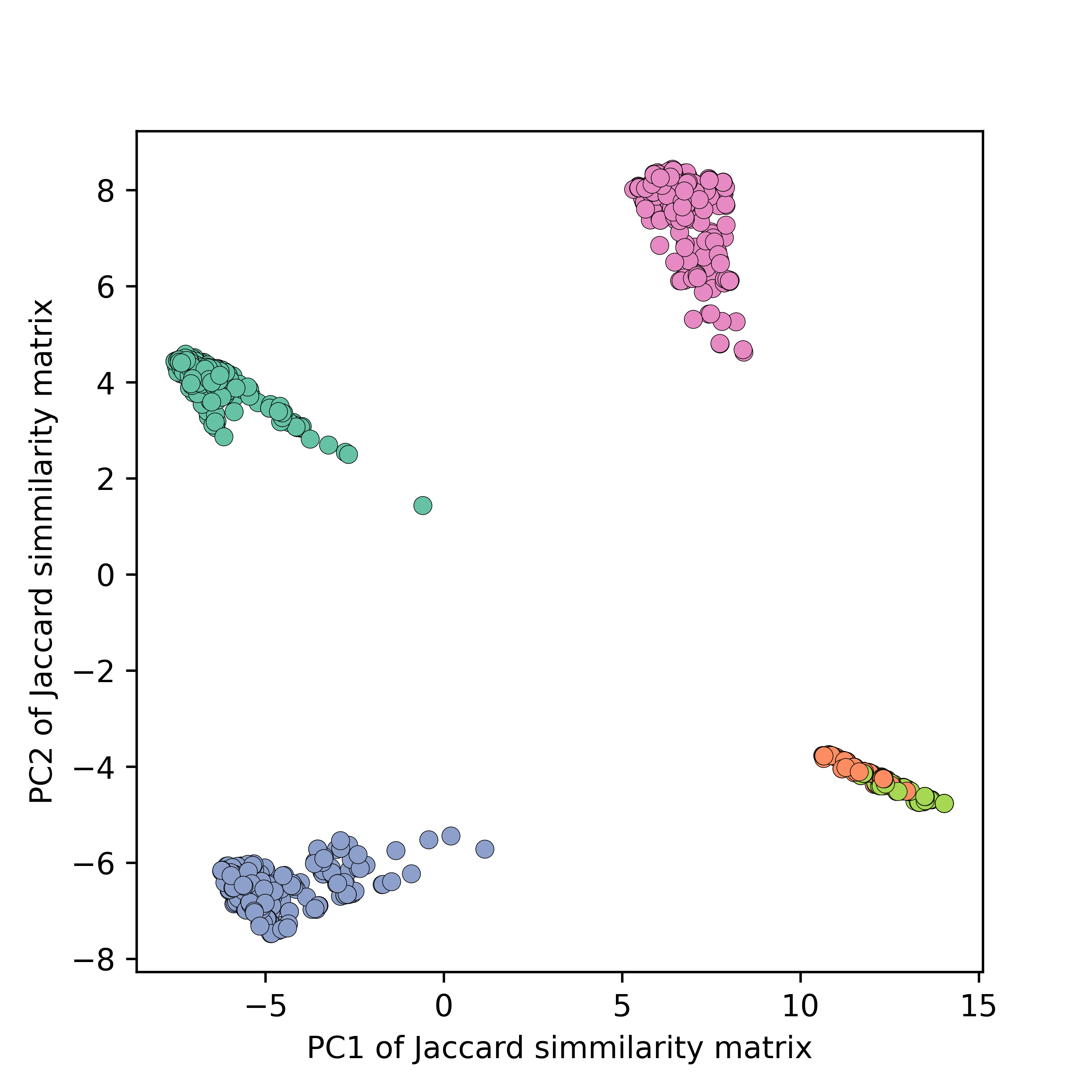

Supplement: Supplementary file 11 — Source data Fig. 3 [file 44320_2025_169_MOESM11_ESM.zip › Figure3_d/Figure_3d.png]

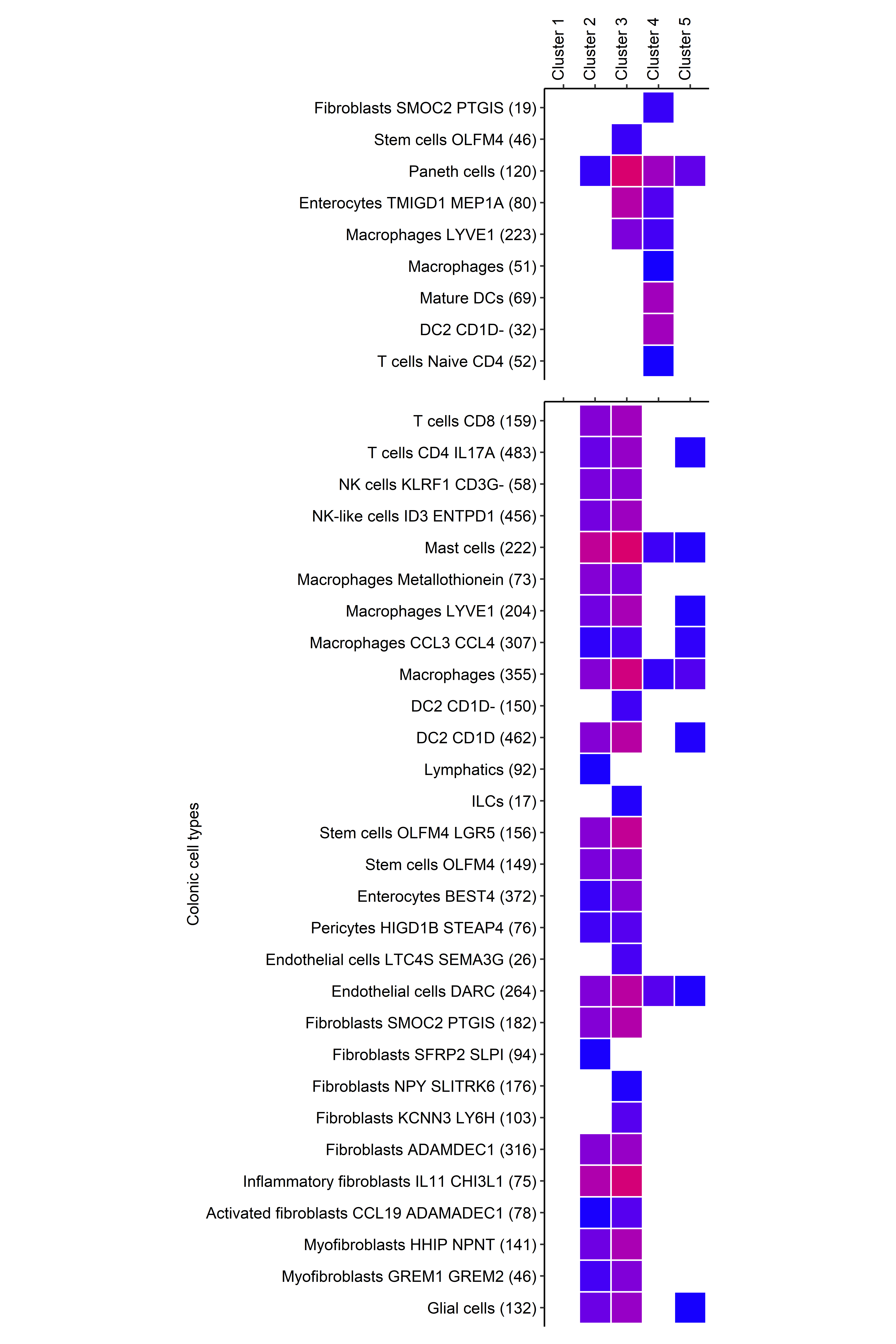

Supplement: Supplementary file 12 — Source data Fig. 4 [file 44320_2025_169_MOESM12_ESM.zip › SC_colon_Kong_et_al_CD_5_clusters_tileplot_ileum_colonwithout_legendv8.png]

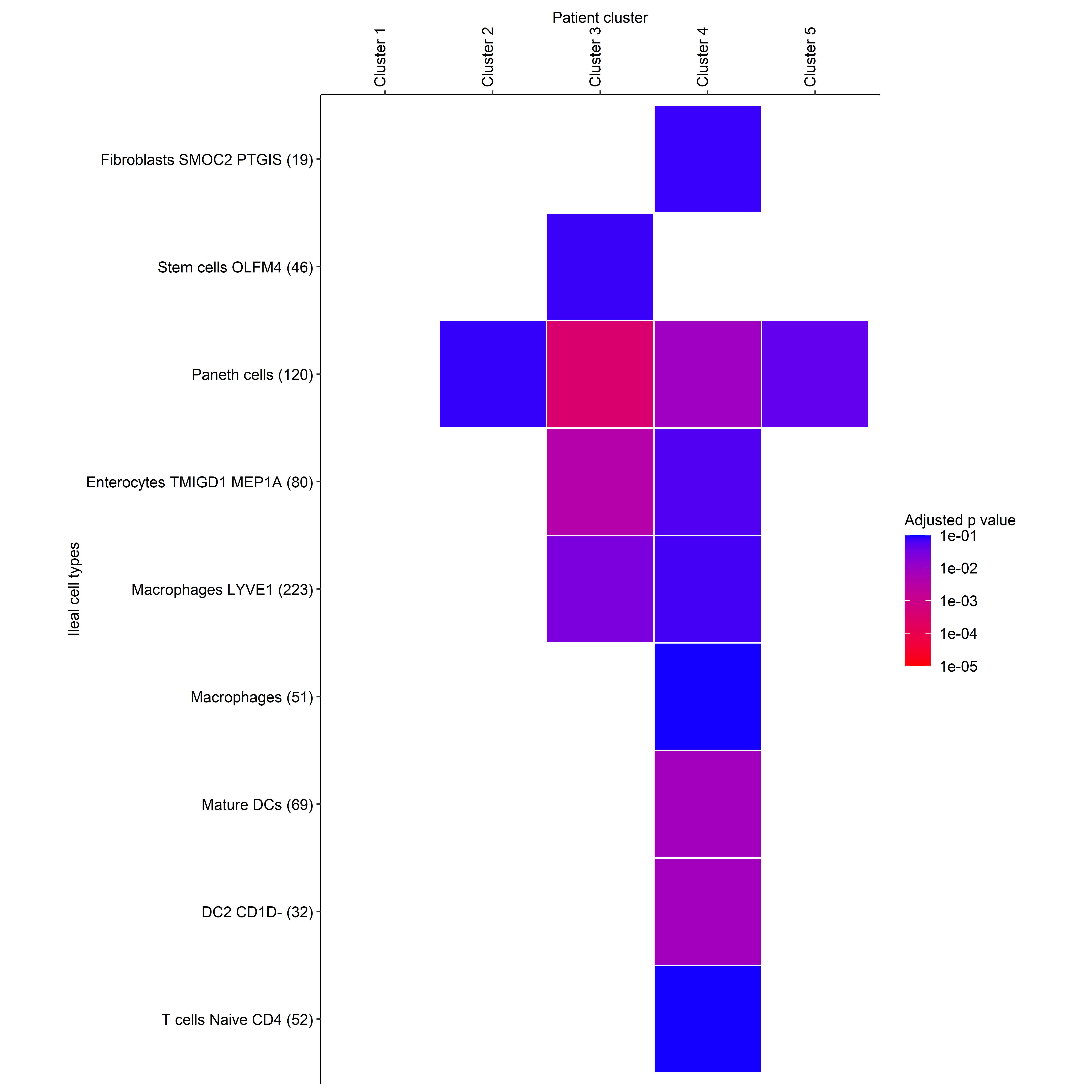

Supplement: Supplementary file 12 — Source data Fig. 4 [file 44320_2025_169_MOESM12_ESM.zip › SC_ileum_Kong_et_al_CD_5_clusters_tilev4_new.png]

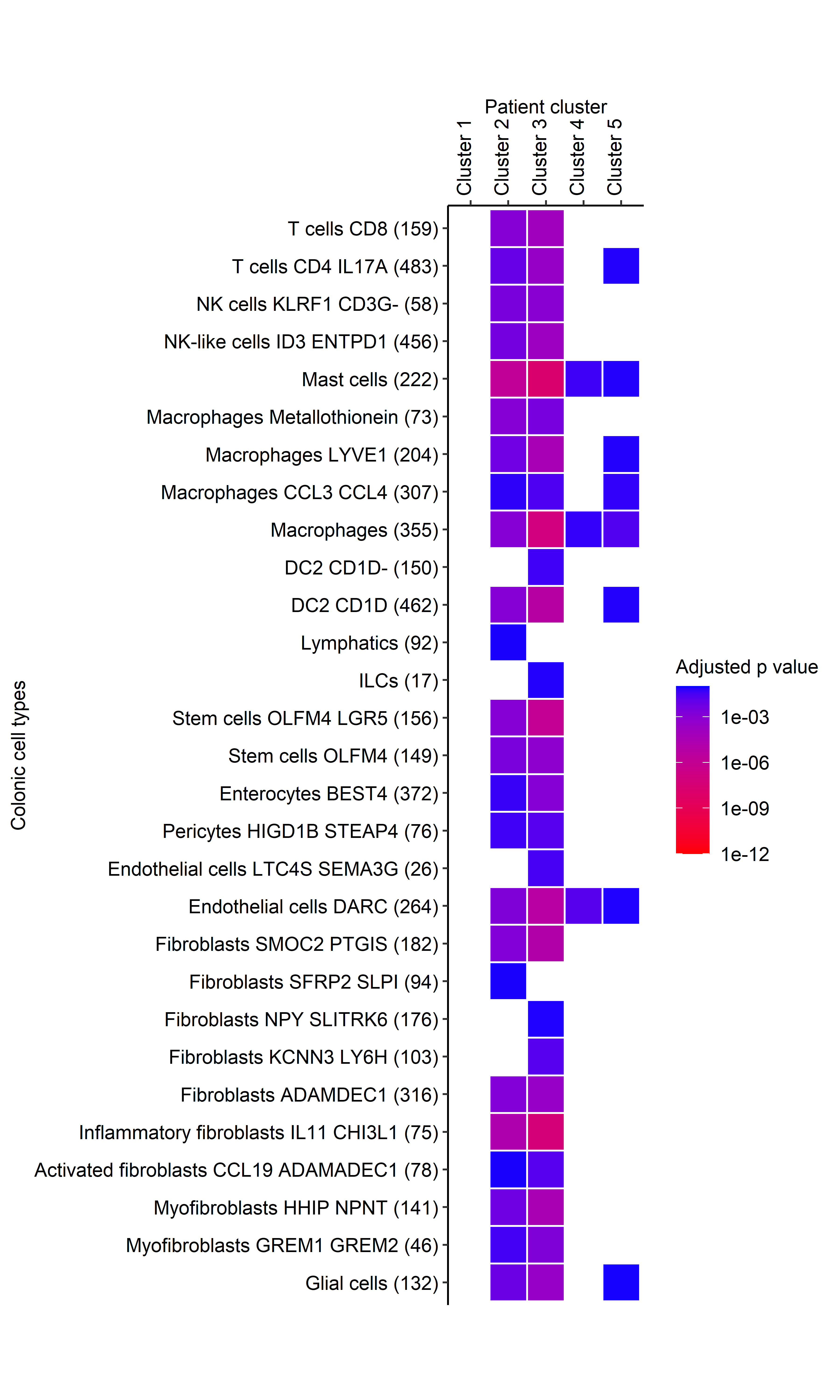

Supplement: Supplementary file 12 — Source data Fig. 4 [file 44320_2025_169_MOESM12_ESM.zip › SC_colon_Kong_et_al_CD_5_clusters_tile1_infl_vs_health_10k_v2.png]

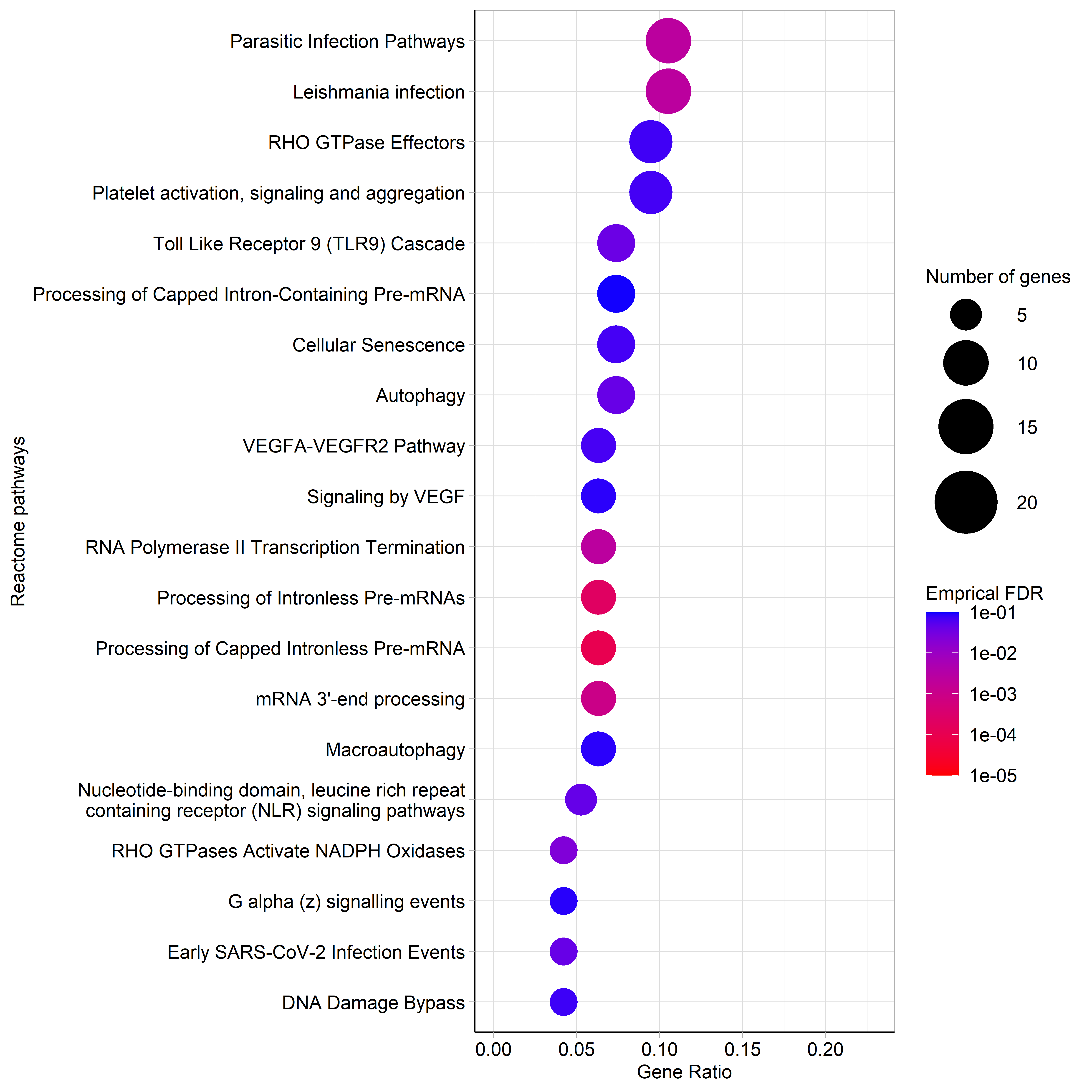

Supplement: Supplementary file 13 — Source data Fig. 5 [file 44320_2025_169_MOESM13_ESM.zip › Figure5_c/Figure5c.png]

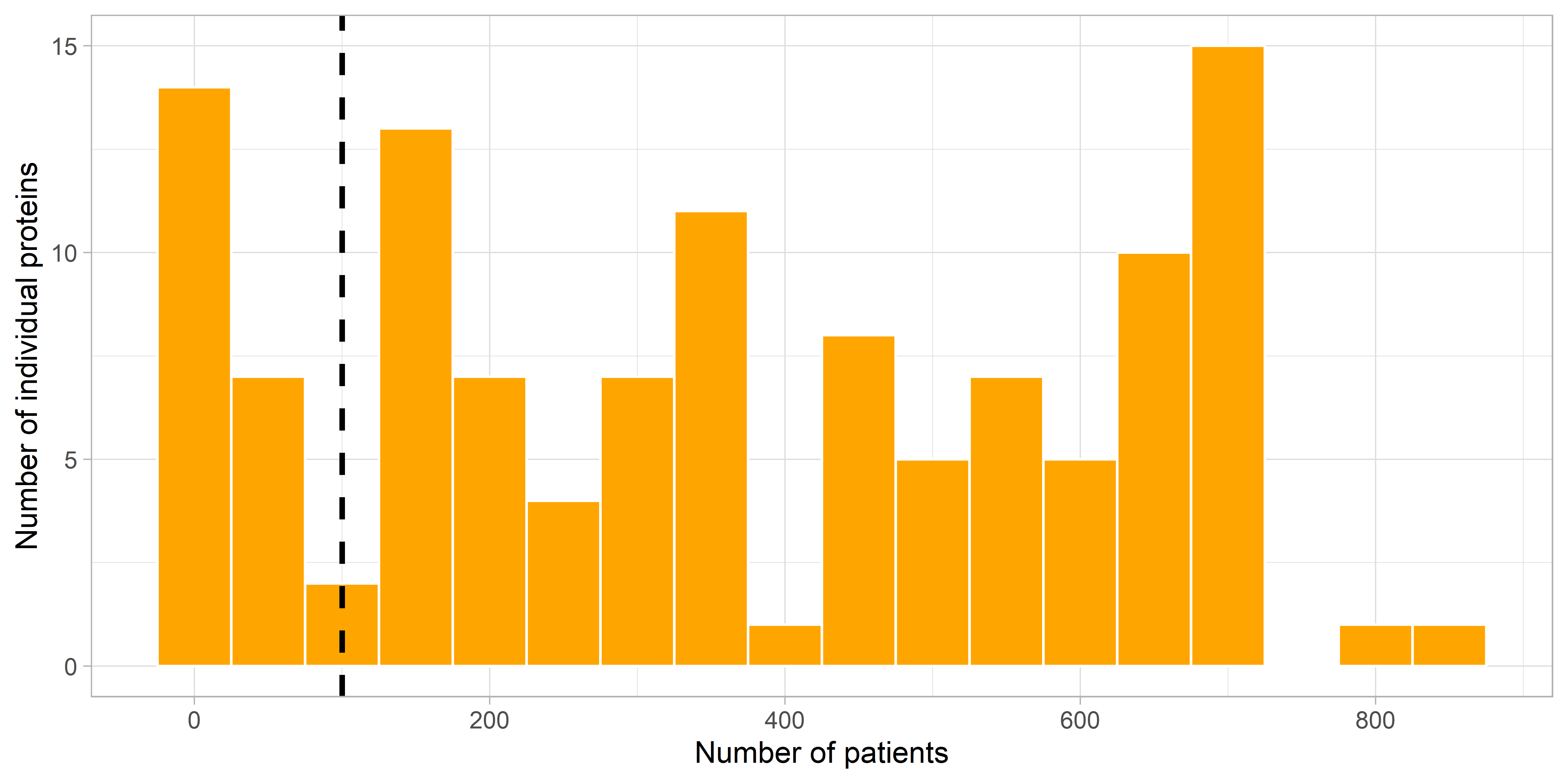

Supplement: Supplementary file 13 — Source data Fig. 5 [file 44320_2025_169_MOESM13_ESM.zip › Figure5_b/Figure5b.png]

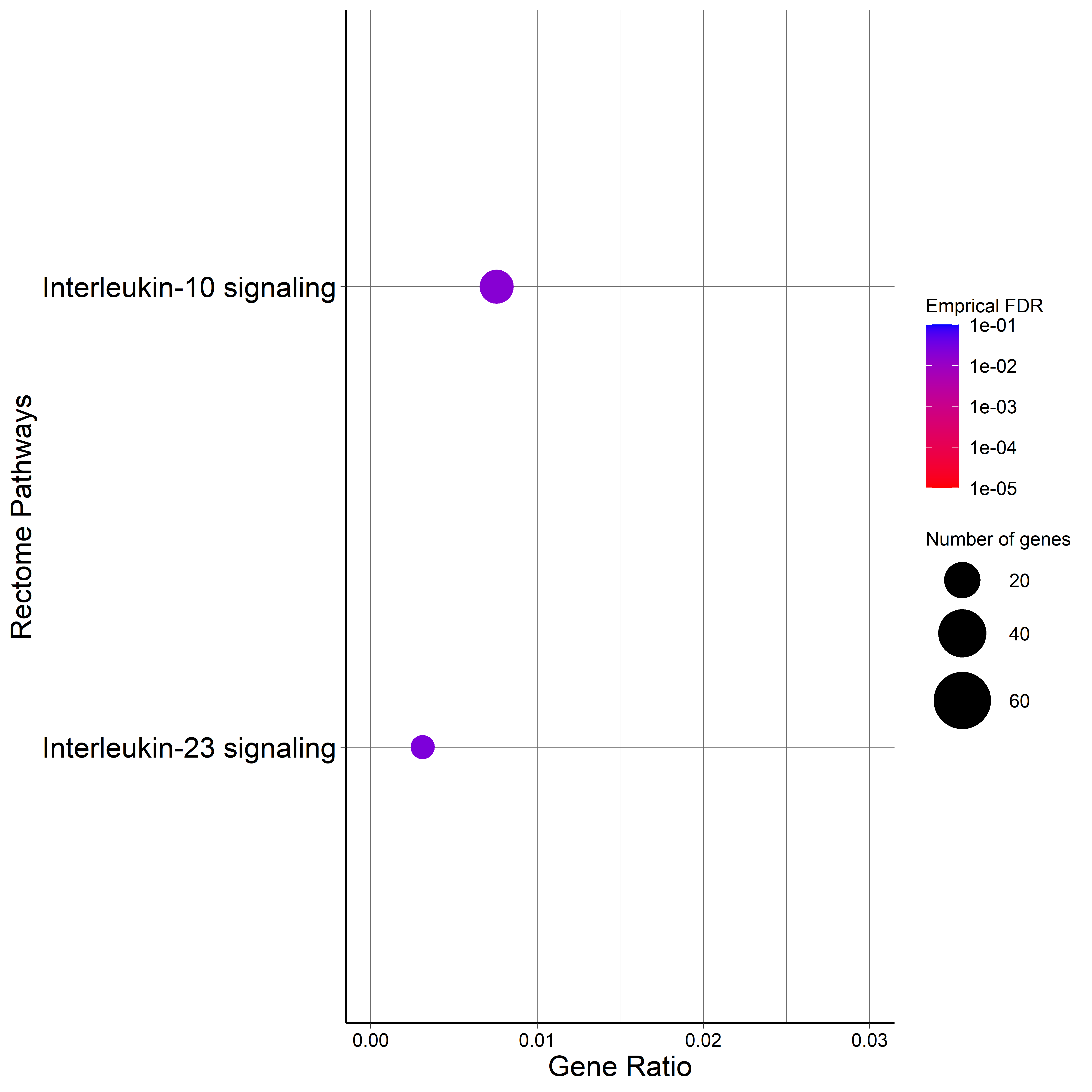

Supplement: Supplementary file 14 — Source data Fig. 6 [file 44320_2025_169_MOESM14_ESM.zip › Figure6_c/Reactome_mulea_UC_TFTGtop100_v4.png]

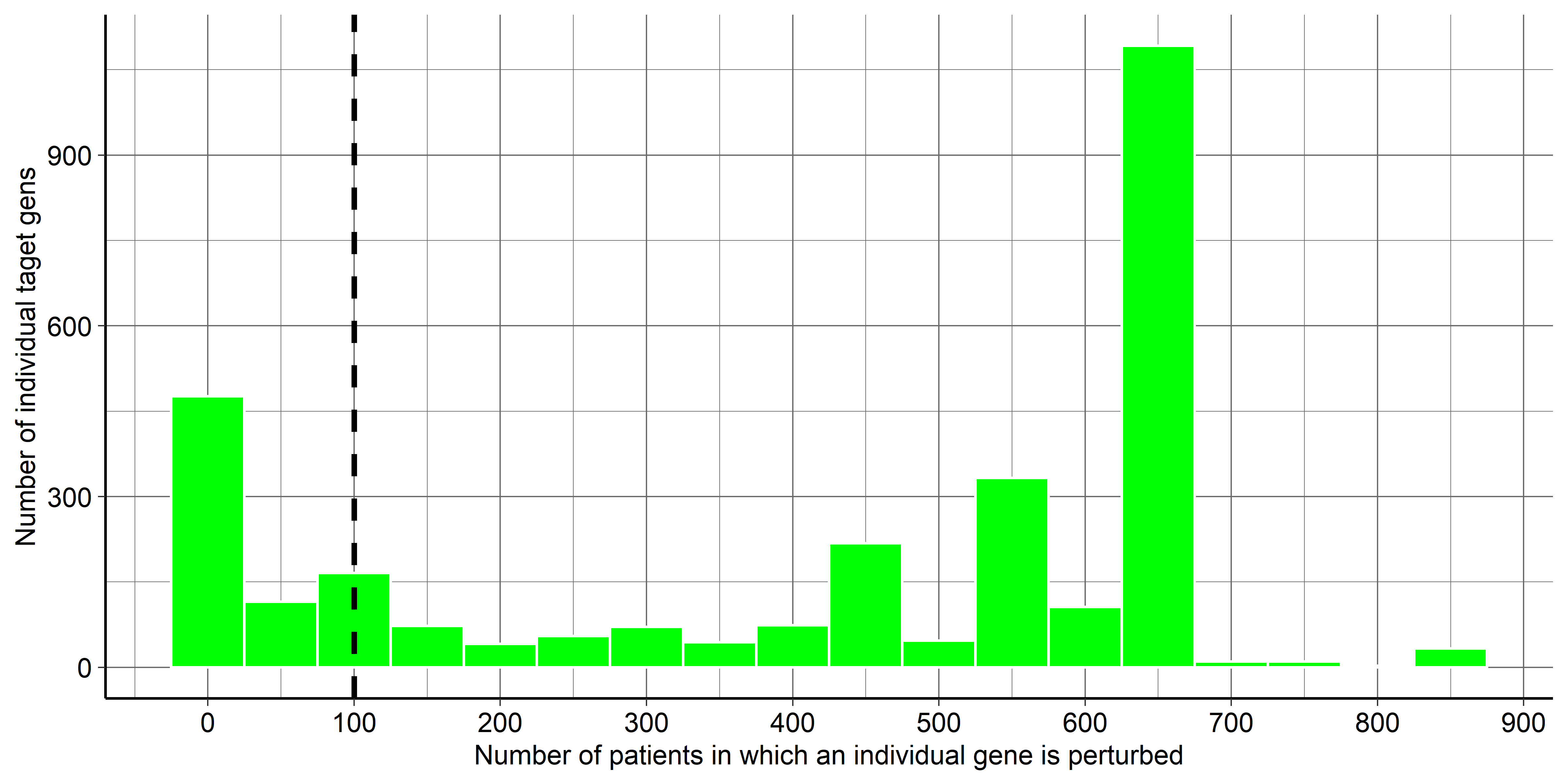

Supplement: Supplementary file 14 — Source data Fig. 6 [file 44320_2025_169_MOESM14_ESM.zip › Figure6_b/Figure6_b.png]

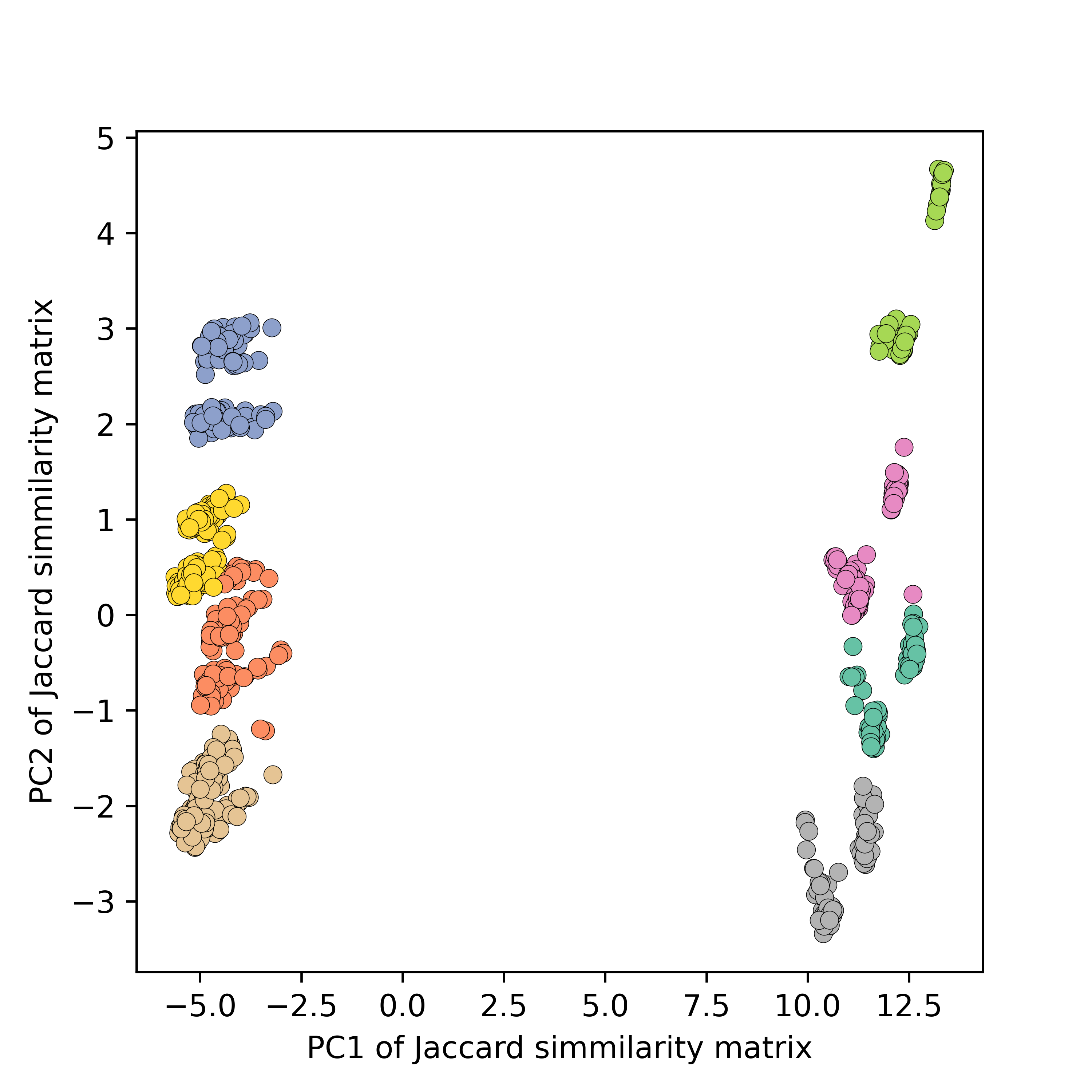

Supplement: Supplementary file 14 — Source data Fig. 6 [file 44320_2025_169_MOESM14_ESM.zip › Figure6_d/Figure_6D.png]
