# Supplementary material for: Decoding non-coding SNPs: systems genomics modelling dissects the heterogeneity of IBD
Source: Mol Syst Biol. 2025 Nov 26;22(2):259–80. doi: 10.1038/s44320-025-00169-3 (PMC12864814; doi:10.1038/s44320-025-00169-3)
Supplement: Supplementary file 13 — Source data Fig. 5 [file 44320_2025_169_MOESM13_ESM.zip › Figure5_c/Figure5c.nb.html]

R Notebook Figure 5c


Code 

- Show All Code
- Hide All Code
- Download Rmd

# R Notebook Figure 5c

Please use the same folder as work directory as the data files. 1.
Remove anything left in the datafiles


```
rm(list=ls())
```


```
If necesearry please install the following packages:2
if (!requireNamespace("BiocManager", quietly = TRUE))
    install.packages("BiocManager")
BiocManager::install("clusterProfiler")
BiocManager::install("ReactomePA") 
BiocManager::install("rrvgo")
BiocManager::install("enrichplot")
BiocManager::install("msigdbr")
BiocManager::install("org.Hs.eg.db")
BiocManager::install("AnnotationDbi")
install.packages("ggplot2")
install.packages("glue")
BiocManager::install("fgsea")
install.packages("mulea")
```


2. Reading in necesearry packages


```
library(clusterProfiler)
library(ReactomePA)
library(rrvgo)
library(enrichplot)
library(ggplot2)
library(msigdbr)
organism ="org.Hs.eg.db"
library(organism, character.only = TRUE)
library(glue)
library(mulea)
library(tidyverse)
library(AnnotationDbi)
library(scales)
```


3. Data input and prepearation for enrtichment analysis THe working
   directory to be ecpected to be the folder of the Rmd file

PPI network propagation output


```
outcome_uc <- read.csv("uc_only_ppi_10k.txt", sep="\t", row.names = 1)
head(outcome_uc)
```


Reactome gene annotation file


```
reactome_ontology <- read_gmt("ReactomePathways.gmt")
```


```
reactome_ontology <- reactome_ontology %>% 
rename(ontology_id = "ontology_id",
    ontology_name = "ontology_name",
    list_of_values = "list_of_values")
head(reactome_ontology)
```


Filtering onytology for minimum 5 and maximmum 400 elments -
excluding really large and really small patheways. We can change theese
settings later.


```
reactome_ontology_filtered <- filter_ontology(gmt = reactome_ontology,
                                        min_nr_of_elements = 5,
                                        max_nr_of_elements = 500)
```


OmniPath newtwork file


```
op <- read.csv("OmniPath_27_10_2021_directed.ncol", sep=" ", header = FALSE, row.names = NULL)
head(op)
```


```
head(outcome_uc)
```


```
filtered_outcome_uc <- outcome_uc[outcome_uc$Z_Count>0,]
head(filtered_outcome_uc)
```


```
bg <- unique(c(op$V1,op$V2))
```


```
# Translating genes in the background and outcome data to gene symbols

# Translate background genes
bg_symbols <- mapIds(org.Hs.eg.db, keys = bg, column = "SYMBOL", keytype = "UNIPROT", multiVals = "first")
```


4. Enrichmnet and creating Figure 5c


```
commonly_afffected_genes_uc <- filtered_outcome_uc[filtered_outcome_uc$Z_Count>100,]
```


```
dim(commonly_afffected_genes_uc)
```


```
[1] 95  4
```


```
commonly_afffected_uc_genes_symbols <- mapIds(org.Hs.eg.db, keys = row.names(commonly_afffected_genes_uc), 
column = "SYMBOL", keytype = "UNIPROT", multiVals = "first")
```


```
ora_model <- ora(gmt = reactome_ontology_filtered, 
                 # Test set variable
                 element_names = commonly_afffected_uc_genes_symbols, 
                 # Background set variable
                 background_element_names = bg_symbols, 
                 # p-value adjustment method
                 p_value_adjustment_method = "eFDR", 
                 # Number of permutations
                 number_of_permutations = 10000,
                 # Number of processor threads to use
                 nthreads = 2, 
                 # Setting a random seed for reproducibility
                 random_seed = 42) 

reactome_results_uc_ppi <- run_test(ora_model)
```


```
reactome_results_uc_ppi$GeneRatio <- reactome_results_uc_ppi$nr_common_with_tested_elements / length(commonly_afffected_uc_genes_symbols)
sig_results_uc_ppi <- reactome_results_uc_ppi %>%
    # Rows where the eFDR < 0.05
    filter(eFDR < 0.1) %>%
    # Arrange the rows by the gene ratio for plotting
    arrange(desc(GeneRatio))
sig_results_uc_ppi$ontology_id <- factor(sig_results_uc_ppi$ontology_id,
levels = sig_results_uc_ppi[order(sig_results_uc_ppi$GeneRatio, decreasing = FALSE), "ontology_id"])
sig_results_uc_ppi
```


```
write.csv(reactome_results_uc_ppi, "Reactome_UC_PPI_100_cutoff_ora_results.csv", row.names = FALSE)
```


Label formatting


```
sig_results_uc_ppi$formed_id <- gsub("_", " ", sig_results_uc_ppi$ontology_id)
sig_results_uc_ppi$formed_id <- factor(sig_results_uc_ppi$formed_id,
                                    levels = sig_results_uc_ppi[order(sig_results_uc_ppi$GeneRatio,
                                    decreasing = FALSE), "formed_id"])
```


Adding a small amount for the fDR calcuation based on the MULEA
calcautions.


```
sig_results_uc_ppi$eFDR = sig_results_uc_ppi$eFDR +10^-12
```


```
dotplot_reactome_uc_ppi <- ggplot(sig_results_uc_ppi[1:20,], aes(x=GeneRatio, y=formed_id)) +
    geom_point(aes(size=nr_common_with_tested_elements, colour=eFDR)) +
    scale_y_discrete(labels = label_wrap(50)) + 
    scale_color_continuous(low="red", high="blue", 
                           limits=c(10^-5, 0.1),
                           trans = "log10", name="Emprical FDR")
                

dotplot_reactome_uc_ppi <- dotplot_reactome_uc_ppi +
    scale_size(limits = c(2,20), range = c(2,15), name = "Number of genes") + 
    xlim(0, 0.23) +
    xlab("Gene Ratio") +
    ylab("Reactome pathways") +
    theme_light() +
    theme(rect=element_rect(fill="white")) +
    theme(panel.background = element_rect(fill = "white")) +
    theme(plot.background = element_rect(fill = "white")) +
    theme(plot.background = element_rect(colour = "white"))+
    theme(axis.title.x = element_text(colour = "black", size = 10)) +
    theme(axis.title.y = element_text(colour = "black", size = 10)) +
    theme(axis.text = element_text(color= "black", size = 10)) +
    theme(axis.line = element_line(color = "black")) +
    theme(legend.background = element_rect(fill ="white")) +
    theme(legend.text = element_text(color="black", size = 10)) +
    theme(legend.title = element_text(color= "black", size = 10))
```


```
dotplot_reactome_uc_ppi
```


```
png(file=glue("Figure5c.png"),width=8, height=8, units="in", res=600)
dotplot_reactome_uc_ppi
dev.off()
```


```
null device 
          1
```


LS0tDQp0aXRsZTogIlIgTm90ZWJvb2sgRmlndXJlIDVjIg0Kb3V0cHV0OiBodG1sX25vdGVib29rDQotLS0NClBsZWFzZSB1c2UgdGhlIHNhbWUgZm9sZGVyIGFzIHdvcmsgZGlyZWN0b3J5IGFzIHRoZSBkYXRhIGZpbGVzLg0KMS4gUmVtb3ZlIGFueXRoaW5nIGxlZnQgaW4gdGhlIGRhdGFmaWxlcw0KYGBge3J9DQpybShsaXN0PWxzKCkpDQpgYGANCmBgYHtyfQ0KSWYgbmVjZXNlYXJyeSBwbGVhc2UgaW5zdGFsbCB0aGUgZm9sbG93aW5nIHBhY2thZ2VzOjINCmlmICghcmVxdWlyZU5hbWVzcGFjZSgiQmlvY01hbmFnZXIiLCBxdWlldGx5ID0gVFJVRSkpDQogICAgaW5zdGFsbC5wYWNrYWdlcygiQmlvY01hbmFnZXIiKQ0KQmlvY01hbmFnZXI6Omluc3RhbGwoImNsdXN0ZXJQcm9maWxlciIpDQpCaW9jTWFuYWdlcjo6aW5zdGFsbCgiUmVhY3RvbWVQQSIpIA0KQmlvY01hbmFnZXI6Omluc3RhbGwoInJydmdvIikNCkJpb2NNYW5hZ2VyOjppbnN0YWxsKCJlbnJpY2hwbG90IikNCkJpb2NNYW5hZ2VyOjppbnN0YWxsKCJtc2lnZGJyIikNCkJpb2NNYW5hZ2VyOjppbnN0YWxsKCJvcmcuSHMuZWcuZGIiKQ0KQmlvY01hbmFnZXI6Omluc3RhbGwoIkFubm90YXRpb25EYmkiKQ0KaW5zdGFsbC5wYWNrYWdlcygiZ2dwbG90MiIpDQppbnN0YWxsLnBhY2thZ2VzKCJnbHVlIikNCkJpb2NNYW5hZ2VyOjppbnN0YWxsKCJmZ3NlYSIpDQppbnN0YWxsLnBhY2thZ2VzKCJtdWxlYSIpDQpgYGANCg0KMi4gUmVhZGluZyBpbiBuZWNlc2VhcnJ5IHBhY2thZ2VzDQpgYGB7cn0NCmxpYnJhcnkoY2x1c3RlclByb2ZpbGVyKQ0KbGlicmFyeShSZWFjdG9tZVBBKQ0KbGlicmFyeShycnZnbykNCmxpYnJhcnkoZW5yaWNocGxvdCkNCmxpYnJhcnkoZ2dwbG90MikNCmxpYnJhcnkobXNpZ2RicikNCm9yZ2FuaXNtID0ib3JnLkhzLmVnLmRiIg0KbGlicmFyeShvcmdhbmlzbSwgY2hhcmFjdGVyLm9ubHkgPSBUUlVFKQ0KbGlicmFyeShnbHVlKQ0KbGlicmFyeShtdWxlYSkNCmxpYnJhcnkodGlkeXZlcnNlKQ0KbGlicmFyeShBbm5vdGF0aW9uRGJpKQ0KbGlicmFyeShzY2FsZXMpDQpgYGANCjMuIERhdGEgaW5wdXQgYW5kIHByZXBlYXJhdGlvbiBmb3IgZW5ydGljaG1lbnQgYW5hbHlzaXMNClRIZSB3b3JraW5nIGRpcmVjdG9yeSB0byBiZSBlY3BlY3RlZCB0byBiZSB0aGUgZm9sZGVyIG9mIHRoZSBSbWQgZmlsZQ0KDQpQUEkgbmV0d29yayBwcm9wYWdhdGlvbiBvdXRwdXQNCmBgYHtyfQ0Kb3V0Y29tZV91YyA8LSByZWFkLmNzdigidWNfb25seV9wcGlfMTBrLnR4dCIsIHNlcD0iXHQiLCByb3cubmFtZXMgPSAxKQ0KaGVhZChvdXRjb21lX3VjKQ0KYGBgDQpSZWFjdG9tZSBnZW5lIGFubm90YXRpb24gZmlsZQ0KYGBge3J9DQpyZWFjdG9tZV9vbnRvbG9neSA8LSByZWFkX2dtdCgiUmVhY3RvbWVQYXRod2F5cy5nbXQiKQ0KYGBgDQpgYGB7cn0NCnJlYWN0b21lX29udG9sb2d5IDwtIHJlYWN0b21lX29udG9sb2d5ICU+JSANCnJlbmFtZShvbnRvbG9neV9pZCA9ICJvbnRvbG9neV9pZCIsDQogICAgb250b2xvZ3lfbmFtZSA9ICJvbnRvbG9neV9uYW1lIiwNCiAgICBsaXN0X29mX3ZhbHVlcyA9ICJsaXN0X29mX3ZhbHVlcyIpDQpoZWFkKHJlYWN0b21lX29udG9sb2d5KSAgICAgIA0KYGBgDQpGaWx0ZXJpbmcgb255dG9sb2d5IGZvciBtaW5pbXVtIDUgYW5kIG1heGltbXVtIDQwMCBlbG1lbnRzIC0gZXhjbHVkaW5nIHJlYWxseSBsYXJnZSBhbmQgcmVhbGx5IHNtYWxsIHBhdGhld2F5cy4gV2UgY2FuIGNoYW5nZSB0aGVlc2Ugc2V0dGluZ3MgbGF0ZXIuDQpgYGB7cn0NCnJlYWN0b21lX29udG9sb2d5X2ZpbHRlcmVkIDwtIGZpbHRlcl9vbnRvbG9neShnbXQgPSByZWFjdG9tZV9vbnRvbG9neSwNCiAgICAgICAgICAgICAgICAgICAgICAgICAgICAgICAgICAgICAgICBtaW5fbnJfb2ZfZWxlbWVudHMgPSA1LA0KICAgICAgICAgICAgICAgICAgICAgICAgICAgICAgICAgICAgICAgIG1heF9ucl9vZl9lbGVtZW50cyA9IDUwMCkNCmBgYA0KT21uaVBhdGggbmV3dHdvcmsgZmlsZQ0KYGBge3J9DQpvcCA8LSByZWFkLmNzdigiT21uaVBhdGhfMjdfMTBfMjAyMV9kaXJlY3RlZC5uY29sIiwgc2VwPSIgIiwgaGVhZGVyID0gRkFMU0UsIHJvdy5uYW1lcyA9IE5VTEwpDQpoZWFkKG9wKQ0KYGBgDQpgYGB7cn0NCmhlYWQob3V0Y29tZV91YykNCmBgYA0KYGBge3J9DQpmaWx0ZXJlZF9vdXRjb21lX3VjIDwtIG91dGNvbWVfdWNbb3V0Y29tZV91YyRaX0NvdW50PjAsXQ0KaGVhZChmaWx0ZXJlZF9vdXRjb21lX3VjKQ0KYGBgDQoNCg0KYGBge3J9DQpiZyA8LSB1bmlxdWUoYyhvcCRWMSxvcCRWMikpDQpgYGANCmBgYHtyfQ0KIyBUcmFuc2xhdGluZyBnZW5lcyBpbiB0aGUgYmFja2dyb3VuZCBhbmQgb3V0Y29tZSBkYXRhIHRvIGdlbmUgc3ltYm9scw0KDQojIFRyYW5zbGF0ZSBiYWNrZ3JvdW5kIGdlbmVzDQpiZ19zeW1ib2xzIDwtIG1hcElkcyhvcmcuSHMuZWcuZGIsIGtleXMgPSBiZywgY29sdW1uID0gIlNZTUJPTCIsIGtleXR5cGUgPSAiVU5JUFJPVCIsIG11bHRpVmFscyA9ICJmaXJzdCIpDQpgYGANCg0KNC4gRW5yaWNobW5ldCBhbmQgY3JlYXRpbmcgRmlndXJlIDVjDQpgYGB7cn0NCmNvbW1vbmx5X2FmZmZlY3RlZF9nZW5lc191YyA8LSBmaWx0ZXJlZF9vdXRjb21lX3VjW2ZpbHRlcmVkX291dGNvbWVfdWMkWl9Db3VudD4xMDAsXSANCmBgYA0KYGBge3J9DQpkaW0oY29tbW9ubHlfYWZmZmVjdGVkX2dlbmVzX3VjKQ0KYGBgDQpgYGB7cn0NCmNvbW1vbmx5X2FmZmZlY3RlZF91Y19nZW5lc19zeW1ib2xzIDwtIG1hcElkcyhvcmcuSHMuZWcuZGIsIGtleXMgPSByb3cubmFtZXMoY29tbW9ubHlfYWZmZmVjdGVkX2dlbmVzX3VjKSwgDQpjb2x1bW4gPSAiU1lNQk9MIiwga2V5dHlwZSA9ICJVTklQUk9UIiwgbXVsdGlWYWxzID0gImZpcnN0IikNCmBgYA0KDQpgYGB7cn0NCm9yYV9tb2RlbCA8LSBvcmEoZ210ID0gcmVhY3RvbWVfb250b2xvZ3lfZmlsdGVyZWQsIA0KICAgICAgICAgICAgICAgICAjIFRlc3Qgc2V0IHZhcmlhYmxlDQogICAgICAgICAgICAgICAgIGVsZW1lbnRfbmFtZXMgPSBjb21tb25seV9hZmZmZWN0ZWRfdWNfZ2VuZXNfc3ltYm9scywgDQogICAgICAgICAgICAgICAgICMgQmFja2dyb3VuZCBzZXQgdmFyaWFibGUNCiAgICAgICAgICAgICAgICAgYmFja2dyb3VuZF9lbGVtZW50X25hbWVzID0gYmdfc3ltYm9scywgDQogICAgICAgICAgICAgICAgICMgcC12YWx1ZSBhZGp1c3RtZW50IG1ldGhvZA0KICAgICAgICAgICAgICAgICBwX3ZhbHVlX2FkanVzdG1lbnRfbWV0aG9kID0gImVGRFIiLCANCiAgICAgICAgICAgICAgICAgIyBOdW1iZXIgb2YgcGVybXV0YXRpb25zDQogICAgICAgICAgICAgICAgIG51bWJlcl9vZl9wZXJtdXRhdGlvbnMgPSAxMDAwMCwNCiAgICAgICAgICAgICAgICAgIyBOdW1iZXIgb2YgcHJvY2Vzc29yIHRocmVhZHMgdG8gdXNlDQogICAgICAgICAgICAgICAgIG50aHJlYWRzID0gMiwgDQogICAgICAgICAgICAgICAgICMgU2V0dGluZyBhIHJhbmRvbSBzZWVkIGZvciByZXByb2R1Y2liaWxpdHkNCiAgICAgICAgICAgICAgICAgcmFuZG9tX3NlZWQgPSA0MikgDQoNCnJlYWN0b21lX3Jlc3VsdHNfdWNfcHBpIDwtIHJ1bl90ZXN0KG9yYV9tb2RlbCkNCmBgYA0KYGBge3J9DQpyZWFjdG9tZV9yZXN1bHRzX3VjX3BwaSRHZW5lUmF0aW8gPC0gcmVhY3RvbWVfcmVzdWx0c191Y19wcGkkbnJfY29tbW9uX3dpdGhfdGVzdGVkX2VsZW1lbnRzIC8gbGVuZ3RoKGNvbW1vbmx5X2FmZmZlY3RlZF91Y19nZW5lc19zeW1ib2xzKQ0Kc2lnX3Jlc3VsdHNfdWNfcHBpIDwtIHJlYWN0b21lX3Jlc3VsdHNfdWNfcHBpICU+JQ0KICAgICMgUm93cyB3aGVyZSB0aGUgZUZEUiA8IDAuMDUNCiAgICBmaWx0ZXIoZUZEUiA8IDAuMSkgJT4lDQogICAgIyBBcnJhbmdlIHRoZSByb3dzIGJ5IHRoZSBnZW5lIHJhdGlvIGZvciBwbG90dGluZw0KICAgIGFycmFuZ2UoZGVzYyhHZW5lUmF0aW8pKQ0Kc2lnX3Jlc3VsdHNfdWNfcHBpJG9udG9sb2d5X2lkIDwtIGZhY3RvcihzaWdfcmVzdWx0c191Y19wcGkkb250b2xvZ3lfaWQsDQpsZXZlbHMgPSBzaWdfcmVzdWx0c191Y19wcGlbb3JkZXIoc2lnX3Jlc3VsdHNfdWNfcHBpJEdlbmVSYXRpbywgZGVjcmVhc2luZyA9IEZBTFNFKSwgIm9udG9sb2d5X2lkIl0pDQpzaWdfcmVzdWx0c191Y19wcGkNCmBgYA0KYGBge3J9DQp3cml0ZS5jc3YocmVhY3RvbWVfcmVzdWx0c191Y19wcGksICJSZWFjdG9tZV9VQ19QUElfMTAwX2N1dG9mZl9vcmFfcmVzdWx0cy5jc3YiLCByb3cubmFtZXMgPSBGQUxTRSkNCmBgYA0KTGFiZWwgZm9ybWF0dGluZw0KYGBge3J9DQpzaWdfcmVzdWx0c191Y19wcGkkZm9ybWVkX2lkIDwtIGdzdWIoIl8iLCAiICIsIHNpZ19yZXN1bHRzX3VjX3BwaSRvbnRvbG9neV9pZCkNCnNpZ19yZXN1bHRzX3VjX3BwaSRmb3JtZWRfaWQgPC0gZmFjdG9yKHNpZ19yZXN1bHRzX3VjX3BwaSRmb3JtZWRfaWQsDQogICAgICAgICAgICAgICAgICAgICAgICAgICAgICAgICAgICBsZXZlbHMgPSBzaWdfcmVzdWx0c191Y19wcGlbb3JkZXIoc2lnX3Jlc3VsdHNfdWNfcHBpJEdlbmVSYXRpbywNCiAgICAgICAgICAgICAgICAgICAgICAgICAgICAgICAgICAgIGRlY3JlYXNpbmcgPSBGQUxTRSksICJmb3JtZWRfaWQiXSkNCg0KYGBgDQpBZGRpbmcgYSBzbWFsbCBhbW91bnQgZm9yIHRoZSBmRFIgY2FsY3VhdGlvbiBiYXNlZCBvbiB0aGUgTVVMRUEgY2FsY2F1dGlvbnMuDQpgYGB7cn0NCnNpZ19yZXN1bHRzX3VjX3BwaSRlRkRSID0gc2lnX3Jlc3VsdHNfdWNfcHBpJGVGRFIgKzEwXi0xMg0KYGBgDQpgYGB7cn0NCmRvdHBsb3RfcmVhY3RvbWVfdWNfcHBpIDwtIGdncGxvdChzaWdfcmVzdWx0c191Y19wcGlbMToyMCxdLCBhZXMoeD1HZW5lUmF0aW8sIHk9Zm9ybWVkX2lkKSkgKw0KICAgIGdlb21fcG9pbnQoYWVzKHNpemU9bnJfY29tbW9uX3dpdGhfdGVzdGVkX2VsZW1lbnRzLCBjb2xvdXI9ZUZEUikpICsNCiAgICBzY2FsZV95X2Rpc2NyZXRlKGxhYmVscyA9IGxhYmVsX3dyYXAoNTApKSArIA0KICAgIHNjYWxlX2NvbG9yX2NvbnRpbnVvdXMobG93PSJyZWQiLCBoaWdoPSJibHVlIiwgDQogICAgICAgICAgICAgICAgICAgICAgICAgICBsaW1pdHM9YygxMF4tNSwgMC4xKSwNCiAgICAgICAgICAgICAgICAgICAgICAgICAgIHRyYW5zID0gImxvZzEwIiwgbmFtZT0iRW1wcmljYWwgRkRSIikNCiAgICAgICAgICAgICAgICANCg0KZG90cGxvdF9yZWFjdG9tZV91Y19wcGkgPC0gZG90cGxvdF9yZWFjdG9tZV91Y19wcGkgKw0KICAgIHNjYWxlX3NpemUobGltaXRzID0gYygyLDIwKSwgcmFuZ2UgPSBjKDIsMTUpLCBuYW1lID0gIk51bWJlciBvZiBnZW5lcyIpICsgDQogICAgeGxpbSgwLCAwLjIzKSArDQogICAgeGxhYigiR2VuZSBSYXRpbyIpICsNCiAgICB5bGFiKCJSZWFjdG9tZSBwYXRod2F5cyIpICsNCiAgICB0aGVtZV9saWdodCgpICsNCiAgICB0aGVtZShyZWN0PWVsZW1lbnRfcmVjdChmaWxsPSJ3aGl0ZSIpKSArDQogICAgdGhlbWUocGFuZWwuYmFja2dyb3VuZCA9IGVsZW1lbnRfcmVjdChmaWxsID0gIndoaXRlIikpICsNCiAgICB0aGVtZShwbG90LmJhY2tncm91bmQgPSBlbGVtZW50X3JlY3QoZmlsbCA9ICJ3aGl0ZSIpKSArDQogICAgdGhlbWUocGxvdC5iYWNrZ3JvdW5kID0gZWxlbWVudF9yZWN0KGNvbG91ciA9ICJ3aGl0ZSIpKSsNCiAgICB0aGVtZShheGlzLnRpdGxlLnggPSBlbGVtZW50X3RleHQoY29sb3VyID0gImJsYWNrIiwgc2l6ZSA9IDEwKSkgKw0KICAgIHRoZW1lKGF4aXMudGl0bGUueSA9IGVsZW1lbnRfdGV4dChjb2xvdXIgPSAiYmxhY2siLCBzaXplID0gMTApKSArDQogICAgdGhlbWUoYXhpcy50ZXh0ID0gZWxlbWVudF90ZXh0KGNvbG9yPSAiYmxhY2siLCBzaXplID0gMTApKSArDQogICAgdGhlbWUoYXhpcy5saW5lID0gZWxlbWVudF9saW5lKGNvbG9yID0gImJsYWNrIikpICsNCiAgICB0aGVtZShsZWdlbmQuYmFja2dyb3VuZCA9IGVsZW1lbnRfcmVjdChmaWxsID0id2hpdGUiKSkgKw0KICAgIHRoZW1lKGxlZ2VuZC50ZXh0ID0gZWxlbWVudF90ZXh0KGNvbG9yPSJibGFjayIsIHNpemUgPSAxMCkpICsNCiAgICB0aGVtZShsZWdlbmQudGl0bGUgPSBlbGVtZW50X3RleHQoY29sb3I9ICJibGFjayIsIHNpemUgPSAxMCkpIA0KYGBgDQpgYGB7cn0NCmRvdHBsb3RfcmVhY3RvbWVfdWNfcHBpDQpgYGANCg0KYGBge3J9DQpwbmcoZmlsZT1nbHVlKCJGaWd1cmU1Yy5wbmciKSx3aWR0aD04LCBoZWlnaHQ9OCwgdW5pdHM9ImluIiwgcmVzPTYwMCkNCmRvdHBsb3RfcmVhY3RvbWVfdWNfcHBpDQpkZXYub2ZmKCkNCmBgYA0K
