# Supplementary material for: Decoding non-coding SNPs: systems genomics modelling dissects the heterogeneity of IBD
Source: Mol Syst Biol. 2025 Nov 26;22(2):259–80. doi: 10.1038/s44320-025-00169-3 (PMC12864814; doi:10.1038/s44320-025-00169-3)
Supplement: Supplementary file 13 — Source data Fig. 5 [file 44320_2025_169_MOESM13_ESM.zip › Figure5_b/Figure5_b.nb.html]

R Notebook Figure 5b


Code 

- Show All Code
- Hide All Code
- Download Rmd

# R Notebook Figure 5b

Please use the same folder as work directory as the data files. 1.
Remove anything left in the datafiles


```
If necesearry please install the following packages:2
if (!requireNamespace("BiocManager", quietly = TRUE))
    install.packages("BiocManager")
BiocManager::install("clusterProfiler")
BiocManager::install("ReactomePA") 
BiocManager::install("rrvgo")
BiocManager::install("enrichplot")
BiocManager::install("msigdbr")
BiocManager::install("org.Hs.eg.db")
BiocManager::install("AnnotationDbi")
install.packages("ggplot2")
install.packages("glue")
BiocManager::install("fgsea")
install.packages("mulea")
```


2. Reading in necesearry packages


```
library(clusterProfiler)
library(ReactomePA)
library(rrvgo)
library(enrichplot)
library(ggplot2)
library(msigdbr)
organism ="org.Hs.eg.db"
library(organism, character.only = TRUE)
library(glue)
library(mulea)
library(tidyverse)
library(AnnotationDbi)
library(scales)
```


3. Data input and prepearation for enrtichment analysis THe working
   directory to be ecpected to be the folder of the Rmd file

PPI network propagation output


```
outcome_uc <- read.csv("uc_only_ppi_10k.txt", sep="\t", row.names = 1)
head(outcome_uc)
```


```
head(outcome_uc)
```


```
filtered_outcome_uc <- outcome_uc[outcome_uc$Z_Count>0,]
head(filtered_outcome_uc)
```


4. Creating Figure 5/b


```
histogramplot_uc <- ggplot(filtered_outcome_uc, aes(x=Z_Count)) + 
    geom_histogram(colour="white", fill="orange", binwidth = 50)+
    ylab("Number of individual proteins") + xlab("Number of patients") +
    geom_vline(aes(xintercept=100),
            color="black", linetype="dashed", size=1) + 
    scale_x_continuous(breaks=seq(0,1400,200)) + theme_light()
    theme(panel.background = element_rect(fill = "black")) +
    theme(plot.background = element_rect(fill = "white", colour = "white")) +
    theme(axis.title.x = element_text(colour = "black", size = 10)) +
    theme(axis.title.y = element_text(colour = "black", size = 10)) +
    theme(axis.text = element_text(color= "black", size = 10)) +
    theme(axis.line = element_line(color = "black")) +
    theme(panel.grid.minor = element_blank())
```


```
List of 7
 $ panel.background:List of 5
  ..$ fill         : chr "black"
  ..$ colour       : NULL
  ..$ linewidth    : NULL
  ..$ linetype     : NULL
  ..$ inherit.blank: logi FALSE
  ..- attr(*, "class")= chr [1:2] "element_rect" "element"
 $ plot.background :List of 5
  ..$ fill         : chr "white"
  ..$ colour       : chr "white"
  ..$ linewidth    : NULL
  ..$ linetype     : NULL
  ..$ inherit.blank: logi FALSE
  ..- attr(*, "class")= chr [1:2] "element_rect" "element"
 $ axis.title.x    :List of 11
  ..$ family       : NULL
  ..$ face         : NULL
  ..$ colour       : chr "black"
  ..$ size         : num 10
  ..$ hjust        : NULL
  ..$ vjust        : NULL
  ..$ angle        : NULL
  ..$ lineheight   : NULL
  ..$ margin       : NULL
  ..$ debug        : NULL
  ..$ inherit.blank: logi FALSE
  ..- attr(*, "class")= chr [1:2] "element_text" "element"
 $ axis.title.y    :List of 11
  ..$ family       : NULL
  ..$ face         : NULL
  ..$ colour       : chr "black"
  ..$ size         : num 10
  ..$ hjust        : NULL
  ..$ vjust        : NULL
  ..$ angle        : NULL
  ..$ lineheight   : NULL
  ..$ margin       : NULL
  ..$ debug        : NULL
  ..$ inherit.blank: logi FALSE
  ..- attr(*, "class")= chr [1:2] "element_text" "element"
 $ axis.text       :List of 11
  ..$ family       : NULL
  ..$ face         : NULL
  ..$ colour       : chr "black"
  ..$ size         : num 10
  ..$ hjust        : NULL
  ..$ vjust        : NULL
  ..$ angle        : NULL
  ..$ lineheight   : NULL
  ..$ margin       : NULL
  ..$ debug        : NULL
  ..$ inherit.blank: logi FALSE
  ..- attr(*, "class")= chr [1:2] "element_text" "element"
 $ axis.line       :List of 6
  ..$ colour       : chr "black"
  ..$ linewidth    : NULL
  ..$ linetype     : NULL
  ..$ lineend      : NULL
  ..$ arrow        : logi FALSE
  ..$ inherit.blank: logi FALSE
  ..- attr(*, "class")= chr [1:2] "element_line" "element"
 $ panel.grid.minor: list()
  ..- attr(*, "class")= chr [1:2] "element_blank" "element"
 - attr(*, "class")= chr [1:2] "theme" "gg"
 - attr(*, "complete")= logi FALSE
 - attr(*, "validate")= logi TRUE
```


```
histogramplot_uc
```


```
png("Figure5b.png",width=8, height=4, units="in", res=600)
histogramplot_uc
dev.off()
```


```
null device 
          1
```


LS0tDQp0aXRsZTogIlIgTm90ZWJvb2sgRmlndXJlIDViIg0Kb3V0cHV0OiBodG1sX25vdGVib29rDQotLS0NClBsZWFzZSB1c2UgdGhlIHNhbWUgZm9sZGVyIGFzIHdvcmsgZGlyZWN0b3J5IGFzIHRoZSBkYXRhIGZpbGVzLg0KMS4gUmVtb3ZlIGFueXRoaW5nIGxlZnQgaW4gdGhlIGRhdGFmaWxlcw0KYGBge3J9DQpybShsaXN0PWxzKCkpDQpgYGANCmBgYHtyfQ0KSWYgbmVjZXNlYXJyeSBwbGVhc2UgaW5zdGFsbCB0aGUgZm9sbG93aW5nIHBhY2thZ2VzOjINCmlmICghcmVxdWlyZU5hbWVzcGFjZSgiQmlvY01hbmFnZXIiLCBxdWlldGx5ID0gVFJVRSkpDQogICAgaW5zdGFsbC5wYWNrYWdlcygiQmlvY01hbmFnZXIiKQ0KQmlvY01hbmFnZXI6Omluc3RhbGwoImNsdXN0ZXJQcm9maWxlciIpDQpCaW9jTWFuYWdlcjo6aW5zdGFsbCgiUmVhY3RvbWVQQSIpIA0KQmlvY01hbmFnZXI6Omluc3RhbGwoInJydmdvIikNCkJpb2NNYW5hZ2VyOjppbnN0YWxsKCJlbnJpY2hwbG90IikNCkJpb2NNYW5hZ2VyOjppbnN0YWxsKCJtc2lnZGJyIikNCkJpb2NNYW5hZ2VyOjppbnN0YWxsKCJvcmcuSHMuZWcuZGIiKQ0KQmlvY01hbmFnZXI6Omluc3RhbGwoIkFubm90YXRpb25EYmkiKQ0KaW5zdGFsbC5wYWNrYWdlcygiZ2dwbG90MiIpDQppbnN0YWxsLnBhY2thZ2VzKCJnbHVlIikNCkJpb2NNYW5hZ2VyOjppbnN0YWxsKCJmZ3NlYSIpDQppbnN0YWxsLnBhY2thZ2VzKCJtdWxlYSIpDQpgYGANCg0KMi4gUmVhZGluZyBpbiBuZWNlc2VhcnJ5IHBhY2thZ2VzDQpgYGB7cn0NCmxpYnJhcnkoY2x1c3RlclByb2ZpbGVyKQ0KbGlicmFyeShSZWFjdG9tZVBBKQ0KbGlicmFyeShycnZnbykNCmxpYnJhcnkoZW5yaWNocGxvdCkNCmxpYnJhcnkoZ2dwbG90MikNCmxpYnJhcnkobXNpZ2RicikNCm9yZ2FuaXNtID0ib3JnLkhzLmVnLmRiIg0KbGlicmFyeShvcmdhbmlzbSwgY2hhcmFjdGVyLm9ubHkgPSBUUlVFKQ0KbGlicmFyeShnbHVlKQ0KbGlicmFyeShtdWxlYSkNCmxpYnJhcnkodGlkeXZlcnNlKQ0KbGlicmFyeShBbm5vdGF0aW9uRGJpKQ0KbGlicmFyeShzY2FsZXMpDQpgYGANCjMuIERhdGEgaW5wdXQgYW5kIHByZXBlYXJhdGlvbiBmb3IgZW5ydGljaG1lbnQgYW5hbHlzaXMNClRIZSB3b3JraW5nIGRpcmVjdG9yeSB0byBiZSBlY3BlY3RlZCB0byBiZSB0aGUgZm9sZGVyIG9mIHRoZSBSbWQgZmlsZQ0KDQpQUEkgbmV0d29yayBwcm9wYWdhdGlvbiBvdXRwdXQNCmBgYHtyfQ0Kb3V0Y29tZV91YyA8LSByZWFkLmNzdigidWNfb25seV9wcGlfMTBrLnR4dCIsIHNlcD0iXHQiLCByb3cubmFtZXMgPSAxKQ0KaGVhZChvdXRjb21lX3VjKQ0KYGBgDQoNCmBgYHtyfQ0KaGVhZChvdXRjb21lX3VjKQ0KYGBgDQpgYGB7cn0NCmZpbHRlcmVkX291dGNvbWVfdWMgPC0gb3V0Y29tZV91Y1tvdXRjb21lX3VjJFpfQ291bnQ+MCxdDQpoZWFkKGZpbHRlcmVkX291dGNvbWVfdWMpDQpgYGANCg0KDQoNCjQuIENyZWF0aW5nIEZpZ3VyZSA1L2INCmBgYHtyfQ0KaGlzdG9ncmFtcGxvdF91YyA8LSBnZ3Bsb3QoZmlsdGVyZWRfb3V0Y29tZV91YywgYWVzKHg9Wl9Db3VudCkpICsgDQogICAgZ2VvbV9oaXN0b2dyYW0oY29sb3VyPSJ3aGl0ZSIsIGZpbGw9Im9yYW5nZSIsIGJpbndpZHRoID0gNTApKw0KICAgIHlsYWIoIk51bWJlciBvZiBpbmRpdmlkdWFsIHByb3RlaW5zIikgKyB4bGFiKCJOdW1iZXIgb2YgcGF0aWVudHMiKSArDQogICAgZ2VvbV92bGluZShhZXMoeGludGVyY2VwdD0xMDApLA0KICAgICAgICAgICAgY29sb3I9ImJsYWNrIiwgbGluZXR5cGU9ImRhc2hlZCIsIHNpemU9MSkgKyANCiAgICBzY2FsZV94X2NvbnRpbnVvdXMoYnJlYWtzPXNlcSgwLDE0MDAsMjAwKSkgKyB0aGVtZV9saWdodCgpDQogICAgdGhlbWUocGFuZWwuYmFja2dyb3VuZCA9IGVsZW1lbnRfcmVjdChmaWxsID0gImJsYWNrIikpICsNCiAgICB0aGVtZShwbG90LmJhY2tncm91bmQgPSBlbGVtZW50X3JlY3QoZmlsbCA9ICJ3aGl0ZSIsIGNvbG91ciA9ICJ3aGl0ZSIpKSArDQogICAgdGhlbWUoYXhpcy50aXRsZS54ID0gZWxlbWVudF90ZXh0KGNvbG91ciA9ICJibGFjayIsIHNpemUgPSAxMCkpICsNCiAgICB0aGVtZShheGlzLnRpdGxlLnkgPSBlbGVtZW50X3RleHQoY29sb3VyID0gImJsYWNrIiwgc2l6ZSA9IDEwKSkgKw0KICAgIHRoZW1lKGF4aXMudGV4dCA9IGVsZW1lbnRfdGV4dChjb2xvcj0gImJsYWNrIiwgc2l6ZSA9IDEwKSkgKw0KICAgIHRoZW1lKGF4aXMubGluZSA9IGVsZW1lbnRfbGluZShjb2xvciA9ICJibGFjayIpKSArDQogICAgdGhlbWUocGFuZWwuZ3JpZC5taW5vciA9IGVsZW1lbnRfYmxhbmsoKSkNCmhpc3RvZ3JhbXBsb3RfdWMNCmBgYA0KDQoNCmBgYHtyfQ0KcG5nKCJGaWd1cmU1Yi5wbmciLHdpZHRoPTgsIGhlaWdodD00LCB1bml0cz0iaW4iLCByZXM9NjAwKQ0KaGlzdG9ncmFtcGxvdF91Yw0KZGV2Lm9mZigpDQpgYGANCg==
