# Supplementary material for: Decoding non-coding SNPs: systems genomics modelling dissects the heterogeneity of IBD
Source: Mol Syst Biol. 2025 Nov 26;22(2):259–80. doi: 10.1038/s44320-025-00169-3 (PMC12864814; doi:10.1038/s44320-025-00169-3)
Supplement: Supplementary file 14 — Source data Fig. 6 [file 44320_2025_169_MOESM14_ESM.zip › Figure6_c/Figure6_B_C.nb.html]

Figure6\_B\_C.knit


Code 

- Show All Code
- Hide All Code
- Download Rmd

Please set the working directory as the folder.

1. Remove anything left in the datafiles


```
rm(list=ls())
```


If necesearry please install the following packages:


```
if (!requireNamespace("BiocManager", quietly = TRUE))
    install.packages("BiocManager")
BiocManager::install("clusterProfiler")
BiocManager::install("ReactomePA") 
BiocManager::install("rrvgo")
BiocManager::install("enrichplot")
BiocManager::install("msigdbr")
BiocManager::install("org.Hs.eg.db")
BiocManager::install("AnnotationDbi")
install.packages("ggplot2")
install.packages("glue")
```


2. Reading in necesearry packages


```
library(clusterProfiler)
library(ReactomePA)
library(rrvgo)
library(enrichplot)
library(ggplot2)
library(msigdbr)
organism ="org.Hs.eg.db"
library(organism, character.only = TRUE)
library(glue)
library(mulea)
library(tidyverse)
library(AnnotationDbi)
library(scales)
```


3. REading infiles UC TF-TG


```
outcometf <- read.csv("uc_TF_TG_network_summary_10k.txt", sep="\t", row.names = 1)
```


```
head(outcometf)
```


```
doro <- read.csv("Dorothea_27_10_2021_ABC.ncol", sep=" ", header = FALSE, row.names = NULL)
```


Checking both TFs and TGs.


```
bgtf <-unique(c(doro$V1,doro$V2))
```


```
bg_TFTG_symbols <- mapIds(org.Hs.eg.db, keys = bgtf, column = "SYMBOL", keytype = "UNIPROT", multiVals = "first")
```


```
reactome_ontology <- read_gmt("ReactomePathways.gmt")
```


```
head(reactome_ontology)
```


```
reactome_ontology <- reactome_ontology %>% 
rename(ontology_id = "ontology_id",
    ontology_name = "ontology_name",
    list_of_values = "list_of_values")
head(reactome_ontology)
```


Filtering onytology for minimum 5 and maximmum 500 elments -
excluding really large and really small patheways. We can change theese
settings later.


```
reactome_ontology_filtered <- filter_ontology(gmt = reactome_ontology,
                                        min_nr_of_elements = 5,
                                        max_nr_of_elements = 500)
```


```
affected_TF_TG_UC <- outcometf[outcometf$Z_Count_TFTG>0,]
```


```
commonly_affected_tftg <- outcometf[outcometf$Z_Count_TFTG>100,]
head(commonly_affected_tftg)
```


Visualising the distribution of affected TGs per TF in UC


```
commonly_afffected_genes_symbols_TFTG <- mapIds(org.Hs.eg.db, keys = row.names(commonly_affected_tftg), column = "SYMBOL", keytype = "UNIPROT", multiVals = "first")
```


```
ora_model <- ora(gmt = reactome_ontology_filtered, 
                 # Test set variable
                 element_names = commonly_afffected_genes_symbols_TFTG, 
                 # Background set variable
                 background_element_names = bg_TFTG_symbols, 
                 # p-value adjustment method
                 p_value_adjustment_method = "eFDR", 
                 # Number of permutations
                 number_of_permutations = 10000,
                 # Number of processor threads to use
                 nthreads = 2, 
                 # Setting a random seed for reproducibility
                 random_seed = 42) 

# Running the ORA
ora_results_tftg <- run_test(ora_model)
```


```
ora_results_tftg <- ora_results_tftg %>% arrange(eFDR, ascending = TRUE)
head(ora_results_tftg)
```


```
ora_results_tftg$GeneRatio <- ora_results_tftg$nr_common_with_tested_elements / length(commonly_afffected_genes_symbols_TFTG)
sig_results_tftg <- ora_results_tftg %>%
    # Rows where the eFDR < 0.05
    filter(eFDR < 0.1) %>%
    # Arrange the rows by the gene ratio for plotting
    arrange(desc(GeneRatio))
sig_results_tftg$ontology_id <- factor(sig_results_tftg$ontology_id,
levels = sig_results_tftg[order(sig_results_tftg$GeneRatio, decreasing = FALSE), "ontology_id"])
sig_results_tftg
```


Writing out Reactome TF-TG results


```
write.csv(ora_results_tftg, glue("Reactome_UC_TFTG_100_cutoff_ora_results.csv"), row.names = TRUE)
```


Label formatting


```
sig_results_tftg$formed_id <- gsub("_", " ", sig_results_tftg$ontology_id)
sig_results_tftg$formed_id <- factor(sig_results_tftg$formed_id,
                                    levels = sig_results_tftg[order(sig_results_tftg$GeneRatio,
                                    decreasing = FALSE), "formed_id"])
```


Visualisation:


```
dotplot_reactome_TFTG_uc <- ggplot(sig_results_tftg, aes(x=GeneRatio, y=formed_id)) +
    geom_point(aes(size=nr_common_with_tested_elements, colour=eFDR)) +
    scale_y_discrete(labels = label_wrap(50)) + 
    scale_color_continuous(low="red", high="blue", 
                           limits=c(10^-5, 0.1),
                           trans = "log10", name="Emprical FDR")
                

dotplot_reactome_TFTG_uc <- dotplot_reactome_TFTG_uc +
    scale_size(limits = c(2,75), range = c(2,15), name = "Number of genes") + 
    xlim(0, 0.03) +
    xlab("Gene Ratio") +
    ylab("Rectome Pathways") +
    theme_dark() +
    theme(legend.key = element_rect(fill ="white")) +
    theme(rect=element_rect(fill="white")) +
    theme(panel.background = element_rect(fill = "white")) +
    theme(plot.background = element_rect(fill = "white")) +
    theme(plot.background = element_rect(colour = "white"))+
    theme(axis.title.x = element_text(colour = "black", size = 15)) +
    theme(axis.title.y = element_text(colour = "black", size = 15)) +
    theme(axis.text.y = element_text(color= "black", size = 15)) +
    theme(axis.text.x = element_text(color= "black", size = 10)) +
    theme(axis.line = element_line(color = "black")) +
    theme(legend.background = element_rect(fill ="white")) +
    theme(legend.text = element_text(color="black", size = 10)) +
    theme(legend.title = element_text(color= "black", size = 10)) 

dotplot_reactome_TFTG_uc
```


```
png(file="Reactome_mulea_UC_TFTGtop100_v4.png",width=8, height=8, units="in", res=600)
dotplot_reactome_TFTG_uc
dev.off()
```


```
null device 
          1
```


LS0tDQp0aXRlbDogIkZpZ3VyZSA2IEMgIg0Kb3V0cHV0OiBodG1sX25vdGVib29rDQprbml0OiByZXByZXg6OnJlcHJleF9yZW5kZXINCi0tLQ0KUGxlYXNlIHNldCB0aGUgd29ya2luZyBkaXJlY3RvcnkgYXMgdGhlIGZvbGRlci4NCg0KMS4gUmVtb3ZlIGFueXRoaW5nIGxlZnQgaW4gdGhlIGRhdGFmaWxlcw0KYGBge3J9DQpybShsaXN0PWxzKCkpDQpgYGANCklmIG5lY2VzZWFycnkgcGxlYXNlIGluc3RhbGwgdGhlIGZvbGxvd2luZyBwYWNrYWdlczoNCmBgYHtyfQ0KaWYgKCFyZXF1aXJlTmFtZXNwYWNlKCJCaW9jTWFuYWdlciIsIHF1aWV0bHkgPSBUUlVFKSkNCiAgICBpbnN0YWxsLnBhY2thZ2VzKCJCaW9jTWFuYWdlciIpDQpCaW9jTWFuYWdlcjo6aW5zdGFsbCgiY2x1c3RlclByb2ZpbGVyIikNCkJpb2NNYW5hZ2VyOjppbnN0YWxsKCJSZWFjdG9tZVBBIikgDQpCaW9jTWFuYWdlcjo6aW5zdGFsbCgicnJ2Z28iKQ0KQmlvY01hbmFnZXI6Omluc3RhbGwoImVucmljaHBsb3QiKQ0KQmlvY01hbmFnZXI6Omluc3RhbGwoIm1zaWdkYnIiKQ0KQmlvY01hbmFnZXI6Omluc3RhbGwoIm9yZy5Icy5lZy5kYiIpDQpCaW9jTWFuYWdlcjo6aW5zdGFsbCgiQW5ub3RhdGlvbkRiaSIpDQppbnN0YWxsLnBhY2thZ2VzKCJnZ3Bsb3QyIikNCmluc3RhbGwucGFja2FnZXMoImdsdWUiKQ0KYGBgDQoyLiBSZWFkaW5nIGluIG5lY2VzZWFycnkgcGFja2FnZXMNCmBgYHtyfQ0KbGlicmFyeShjbHVzdGVyUHJvZmlsZXIpDQpsaWJyYXJ5KFJlYWN0b21lUEEpDQpsaWJyYXJ5KHJydmdvKQ0KbGlicmFyeShlbnJpY2hwbG90KQ0KbGlicmFyeShnZ3Bsb3QyKQ0KbGlicmFyeShtc2lnZGJyKQ0Kb3JnYW5pc20gPSJvcmcuSHMuZWcuZGIiDQpsaWJyYXJ5KG9yZ2FuaXNtLCBjaGFyYWN0ZXIub25seSA9IFRSVUUpDQpsaWJyYXJ5KGdsdWUpDQpsaWJyYXJ5KG11bGVhKQ0KbGlicmFyeSh0aWR5dmVyc2UpDQpsaWJyYXJ5KEFubm90YXRpb25EYmkpDQpsaWJyYXJ5KHNjYWxlcykNCmBgYA0KMy4gUkVhZGluZyBpbmZpbGVzIFVDIFRGLVRHDQpgYGB7cn0NCm91dGNvbWV0ZiA8LSByZWFkLmNzdigidWNfVEZfVEdfbmV0d29ya19zdW1tYXJ5XzEway50eHQiLCBzZXA9Ilx0Iiwgcm93Lm5hbWVzID0gMSkNCmBgYA0KYGBge3J9DQpoZWFkKG91dGNvbWV0ZikNCmBgYA0KYGBge3J9DQpkb3JvIDwtIHJlYWQuY3N2KCJEb3JvdGhlYV8yN18xMF8yMDIxX0FCQy5uY29sIiwgc2VwPSIgIiwgaGVhZGVyID0gRkFMU0UsIHJvdy5uYW1lcyA9IE5VTEwpDQpgYGANCkNoZWNraW5nIGJvdGggVEZzIGFuZCBUR3MuIA0KYGBge3J9DQpiZ3RmIDwtdW5pcXVlKGMoZG9ybyRWMSxkb3JvJFYyKSkNCmBgYA0KYGBge3J9DQpiZ19URlRHX3N5bWJvbHMgPC0gbWFwSWRzKG9yZy5Icy5lZy5kYiwga2V5cyA9IGJndGYsIGNvbHVtbiA9ICJTWU1CT0wiLCBrZXl0eXBlID0gIlVOSVBST1QiLCBtdWx0aVZhbHMgPSAiZmlyc3QiKQ0KYGBgDQpgYGB7cn0NCnJlYWN0b21lX29udG9sb2d5IDwtIHJlYWRfZ210KCJSZWFjdG9tZVBhdGh3YXlzLmdtdCIpDQpgYGANCmBgYHtyfQ0KaGVhZChyZWFjdG9tZV9vbnRvbG9neSkNCmBgYA0KYGBge3J9DQpyZWFjdG9tZV9vbnRvbG9neSA8LSByZWFjdG9tZV9vbnRvbG9neSAlPiUgDQpyZW5hbWUob250b2xvZ3lfaWQgPSAib250b2xvZ3lfaWQiLA0KICAgIG9udG9sb2d5X25hbWUgPSAib250b2xvZ3lfbmFtZSIsDQogICAgbGlzdF9vZl92YWx1ZXMgPSAibGlzdF9vZl92YWx1ZXMiKQ0KaGVhZChyZWFjdG9tZV9vbnRvbG9neSkgICAgICANCmBgYA0KRmlsdGVyaW5nIG9ueXRvbG9neSBmb3IgbWluaW11bSA1IGFuZCBtYXhpbW11bSA1MDAgZWxtZW50cyAtIGV4Y2x1ZGluZyByZWFsbHkgbGFyZ2UgYW5kIHJlYWxseSBzbWFsbCBwYXRoZXdheXMuIFdlIGNhbiBjaGFuZ2UgdGhlZXNlIHNldHRpbmdzIGxhdGVyLg0KYGBge3J9DQpyZWFjdG9tZV9vbnRvbG9neV9maWx0ZXJlZCA8LSBmaWx0ZXJfb250b2xvZ3koZ210ID0gcmVhY3RvbWVfb250b2xvZ3ksDQogICAgICAgICAgICAgICAgICAgICAgICAgICAgICAgICAgICAgICAgbWluX25yX29mX2VsZW1lbnRzID0gNSwNCiAgICAgICAgICAgICAgICAgICAgICAgICAgICAgICAgICAgICAgICBtYXhfbnJfb2ZfZWxlbWVudHMgPSA1MDApDQpgYGANCmBgYHtyfQ0KYWZmZWN0ZWRfVEZfVEdfVUMgPC0gb3V0Y29tZXRmW291dGNvbWV0ZiRaX0NvdW50X1RGVEc+MCxdDQpgYGANCmBgYHtyfQ0KY29tbW9ubHlfYWZmZWN0ZWRfdGZ0ZyA8LSBvdXRjb21ldGZbb3V0Y29tZXRmJFpfQ291bnRfVEZURz4xMDAsXQ0KaGVhZChjb21tb25seV9hZmZlY3RlZF90ZnRnKQ0KYGBgDQpWaXN1YWxpc2luZyB0aGUgZGlzdHJpYnV0aW9uIG9mIGFmZmVjdGVkIFRHcyBwZXIgVEYgaW4gVUMNCg0KYGBge3J9DQpjb21tb25seV9hZmZmZWN0ZWRfZ2VuZXNfc3ltYm9sc19URlRHIDwtIG1hcElkcyhvcmcuSHMuZWcuZGIsIGtleXMgPSByb3cubmFtZXMoY29tbW9ubHlfYWZmZWN0ZWRfdGZ0ZyksIGNvbHVtbiA9ICJTWU1CT0wiLCBrZXl0eXBlID0gIlVOSVBST1QiLCBtdWx0aVZhbHMgPSAiZmlyc3QiKQ0KYGBgDQoNCmBgYHtyfQ0Kb3JhX21vZGVsIDwtIG9yYShnbXQgPSByZWFjdG9tZV9vbnRvbG9neV9maWx0ZXJlZCwgDQogICAgICAgICAgICAgICAgICMgVGVzdCBzZXQgdmFyaWFibGUNCiAgICAgICAgICAgICAgICAgZWxlbWVudF9uYW1lcyA9IGNvbW1vbmx5X2FmZmZlY3RlZF9nZW5lc19zeW1ib2xzX1RGVEcsIA0KICAgICAgICAgICAgICAgICAjIEJhY2tncm91bmQgc2V0IHZhcmlhYmxlDQogICAgICAgICAgICAgICAgIGJhY2tncm91bmRfZWxlbWVudF9uYW1lcyA9IGJnX1RGVEdfc3ltYm9scywgDQogICAgICAgICAgICAgICAgICMgcC12YWx1ZSBhZGp1c3RtZW50IG1ldGhvZA0KICAgICAgICAgICAgICAgICBwX3ZhbHVlX2FkanVzdG1lbnRfbWV0aG9kID0gImVGRFIiLCANCiAgICAgICAgICAgICAgICAgIyBOdW1iZXIgb2YgcGVybXV0YXRpb25zDQogICAgICAgICAgICAgICAgIG51bWJlcl9vZl9wZXJtdXRhdGlvbnMgPSAxMDAwMCwNCiAgICAgICAgICAgICAgICAgIyBOdW1iZXIgb2YgcHJvY2Vzc29yIHRocmVhZHMgdG8gdXNlDQogICAgICAgICAgICAgICAgIG50aHJlYWRzID0gMiwgDQogICAgICAgICAgICAgICAgICMgU2V0dGluZyBhIHJhbmRvbSBzZWVkIGZvciByZXByb2R1Y2liaWxpdHkNCiAgICAgICAgICAgICAgICAgcmFuZG9tX3NlZWQgPSA0MikgDQoNCiMgUnVubmluZyB0aGUgT1JBDQpvcmFfcmVzdWx0c190ZnRnIDwtIHJ1bl90ZXN0KG9yYV9tb2RlbCkNCmBgYA0KDQpgYGB7cn0NCm9yYV9yZXN1bHRzX3RmdGcgPC0gb3JhX3Jlc3VsdHNfdGZ0ZyAlPiUgYXJyYW5nZShlRkRSLCBhc2NlbmRpbmcgPSBUUlVFKQ0KaGVhZChvcmFfcmVzdWx0c190ZnRnKQ0KYGBgDQoNCmBgYHtyfQ0Kb3JhX3Jlc3VsdHNfdGZ0ZyRHZW5lUmF0aW8gPC0gb3JhX3Jlc3VsdHNfdGZ0ZyRucl9jb21tb25fd2l0aF90ZXN0ZWRfZWxlbWVudHMgLyBsZW5ndGgoY29tbW9ubHlfYWZmZmVjdGVkX2dlbmVzX3N5bWJvbHNfVEZURykNCnNpZ19yZXN1bHRzX3RmdGcgPC0gb3JhX3Jlc3VsdHNfdGZ0ZyAlPiUNCiAgICAjIFJvd3Mgd2hlcmUgdGhlIGVGRFIgPCAwLjA1DQogICAgZmlsdGVyKGVGRFIgPCAwLjEpICU+JQ0KICAgICMgQXJyYW5nZSB0aGUgcm93cyBieSB0aGUgZ2VuZSByYXRpbyBmb3IgcGxvdHRpbmcNCiAgICBhcnJhbmdlKGRlc2MoR2VuZVJhdGlvKSkNCnNpZ19yZXN1bHRzX3RmdGckb250b2xvZ3lfaWQgPC0gZmFjdG9yKHNpZ19yZXN1bHRzX3RmdGckb250b2xvZ3lfaWQsDQpsZXZlbHMgPSBzaWdfcmVzdWx0c190ZnRnW29yZGVyKHNpZ19yZXN1bHRzX3RmdGckR2VuZVJhdGlvLCBkZWNyZWFzaW5nID0gRkFMU0UpLCAib250b2xvZ3lfaWQiXSkNCnNpZ19yZXN1bHRzX3RmdGcNCmBgYA0KDQpXcml0aW5nIG91dCBSZWFjdG9tZSBURi1URyByZXN1bHRzDQpgYGB7cn0NCndyaXRlLmNzdihvcmFfcmVzdWx0c190ZnRnLCBnbHVlKCJSZWFjdG9tZV9VQ19URlRHXzEwMF9jdXRvZmZfb3JhX3Jlc3VsdHMuY3N2IiksIHJvdy5uYW1lcyA9IFRSVUUpDQpgYGANCg0KTGFiZWwgZm9ybWF0dGluZw0KYGBge3J9DQpzaWdfcmVzdWx0c190ZnRnJGZvcm1lZF9pZCA8LSBnc3ViKCJfIiwgIiAiLCBzaWdfcmVzdWx0c190ZnRnJG9udG9sb2d5X2lkKQ0Kc2lnX3Jlc3VsdHNfdGZ0ZyRmb3JtZWRfaWQgPC0gZmFjdG9yKHNpZ19yZXN1bHRzX3RmdGckZm9ybWVkX2lkLA0KICAgICAgICAgICAgICAgICAgICAgICAgICAgICAgICAgICAgbGV2ZWxzID0gc2lnX3Jlc3VsdHNfdGZ0Z1tvcmRlcihzaWdfcmVzdWx0c190ZnRnJEdlbmVSYXRpbywNCiAgICAgICAgICAgICAgICAgICAgICAgICAgICAgICAgICAgIGRlY3JlYXNpbmcgPSBGQUxTRSksICJmb3JtZWRfaWQiXSkNCg0KYGBgDQoNClZpc3VhbGlzYXRpb246DQpgYGB7cn0NCmRvdHBsb3RfcmVhY3RvbWVfVEZUR191YyA8LSBnZ3Bsb3Qoc2lnX3Jlc3VsdHNfdGZ0ZywgYWVzKHg9R2VuZVJhdGlvLCB5PWZvcm1lZF9pZCkpICsNCiAgICBnZW9tX3BvaW50KGFlcyhzaXplPW5yX2NvbW1vbl93aXRoX3Rlc3RlZF9lbGVtZW50cywgY29sb3VyPWVGRFIpKSArDQogICAgc2NhbGVfeV9kaXNjcmV0ZShsYWJlbHMgPSBsYWJlbF93cmFwKDUwKSkgKyANCiAgICBzY2FsZV9jb2xvcl9jb250aW51b3VzKGxvdz0icmVkIiwgaGlnaD0iYmx1ZSIsIA0KICAgICAgICAgICAgICAgICAgICAgICAgICAgbGltaXRzPWMoMTBeLTUsIDAuMSksDQogICAgICAgICAgICAgICAgICAgICAgICAgICB0cmFucyA9ICJsb2cxMCIsIG5hbWU9IkVtcHJpY2FsIEZEUiIpDQogICAgICAgICAgICAgICAgDQoNCmRvdHBsb3RfcmVhY3RvbWVfVEZUR191YyA8LSBkb3RwbG90X3JlYWN0b21lX1RGVEdfdWMgKw0KICAgIHNjYWxlX3NpemUobGltaXRzID0gYygyLDc1KSwgcmFuZ2UgPSBjKDIsMTUpLCBuYW1lID0gIk51bWJlciBvZiBnZW5lcyIpICsgDQogICAgeGxpbSgwLCAwLjAzKSArDQogICAgeGxhYigiR2VuZSBSYXRpbyIpICsNCiAgICB5bGFiKCJSZWN0b21lIFBhdGh3YXlzIikgKw0KICAgIHRoZW1lX2RhcmsoKSArDQogICAgdGhlbWUobGVnZW5kLmtleSA9IGVsZW1lbnRfcmVjdChmaWxsID0id2hpdGUiKSkgKw0KICAgIHRoZW1lKHJlY3Q9ZWxlbWVudF9yZWN0KGZpbGw9IndoaXRlIikpICsNCiAgICB0aGVtZShwYW5lbC5iYWNrZ3JvdW5kID0gZWxlbWVudF9yZWN0KGZpbGwgPSAid2hpdGUiKSkgKw0KICAgIHRoZW1lKHBsb3QuYmFja2dyb3VuZCA9IGVsZW1lbnRfcmVjdChmaWxsID0gIndoaXRlIikpICsNCiAgICB0aGVtZShwbG90LmJhY2tncm91bmQgPSBlbGVtZW50X3JlY3QoY29sb3VyID0gIndoaXRlIikpKw0KICAgIHRoZW1lKGF4aXMudGl0bGUueCA9IGVsZW1lbnRfdGV4dChjb2xvdXIgPSAiYmxhY2siLCBzaXplID0gMTUpKSArDQogICAgdGhlbWUoYXhpcy50aXRsZS55ID0gZWxlbWVudF90ZXh0KGNvbG91ciA9ICJibGFjayIsIHNpemUgPSAxNSkpICsNCiAgICB0aGVtZShheGlzLnRleHQueSA9IGVsZW1lbnRfdGV4dChjb2xvcj0gImJsYWNrIiwgc2l6ZSA9IDE1KSkgKw0KICAgIHRoZW1lKGF4aXMudGV4dC54ID0gZWxlbWVudF90ZXh0KGNvbG9yPSAiYmxhY2siLCBzaXplID0gMTApKSArDQogICAgdGhlbWUoYXhpcy5saW5lID0gZWxlbWVudF9saW5lKGNvbG9yID0gImJsYWNrIikpICsNCiAgICB0aGVtZShsZWdlbmQuYmFja2dyb3VuZCA9IGVsZW1lbnRfcmVjdChmaWxsID0id2hpdGUiKSkgKw0KICAgIHRoZW1lKGxlZ2VuZC50ZXh0ID0gZWxlbWVudF90ZXh0KGNvbG9yPSJibGFjayIsIHNpemUgPSAxMCkpICsNCiAgICB0aGVtZShsZWdlbmQudGl0bGUgPSBlbGVtZW50X3RleHQoY29sb3I9ICJibGFjayIsIHNpemUgPSAxMCkpIA0KDQpkb3RwbG90X3JlYWN0b21lX1RGVEdfdWMNCmBgYA0KDQpgYGB7cn0NCnBuZyhmaWxlPSJSZWFjdG9tZV9tdWxlYV9VQ19URlRHdG9wMTAwX3Y0LnBuZyIsd2lkdGg9OCwgaGVpZ2h0PTgsIHVuaXRzPSJpbiIsIHJlcz02MDApDQpkb3RwbG90X3JlYWN0b21lX1RGVEdfdWMNCmRldi5vZmYoKQ0KYGBgDQo=
