# Supplementary material for: Decoding non-coding SNPs: systems genomics modelling dissects the heterogeneity of IBD
Source: Mol Syst Biol. 2025 Nov 26;22(2):259–80. doi: 10.1038/s44320-025-00169-3 (PMC12864814; doi:10.1038/s44320-025-00169-3)
Supplement: Supplementary file 14 — Source data Fig. 6 [file 44320_2025_169_MOESM14_ESM.zip › Figure6_b/Figure6_B.nb.html]

Figure6\_B.knit


Code 

- Show All Code
- Hide All Code
- Download Rmd

1. Remove anything left in the datafiles


```
rm(list=ls())
```


If necesearry please install the following packages:

if (!requireNamespace(“BiocManager”, quietly = TRUE))
install.packages(“BiocManager”) BiocManager::install(“clusterProfiler”)
BiocManager::install(“ReactomePA”) BiocManager::install(“rrvgo”)
BiocManager::install(“enrichplot”) BiocManager::install(“msigdbr”)
BiocManager::install(“org.Hs.eg.db”)
BiocManager::install(“AnnotationDbi”) install.packages(“ggplot2”)
install.packages(“glue”)

2. Reading in necesearry packages


```
library(clusterProfiler)
library(ReactomePA)
library(rrvgo)
library(enrichplot)
library(ggplot2)
library(msigdbr)
organism ="org.Hs.eg.db"
library(organism, character.only = TRUE)
library(glue)
library(mulea)
library(tidyverse)
library(AnnotationDbi)
library(scales)
```


3. Doing the same for UC TF-TG


```
outcometf <- read.csv("uc_TF_TG_network_summary_10k.txt", sep="\t", row.names = 1)
```


```
head(outcometf)
```


```
affected_TF_TG_UC <- outcometf[outcometf$Z_Count_TFTG>0,]
```


Visualising the distribution of affected TGs per TF in UC


```
histogramplot_uc_tf <- ggplot(affected_TF_TG_UC, aes(x=Z_Count_TFTG)) + 
    geom_histogram(colour="white", fill="green", binwidth = 50)+
    ylab("Number of individual taget gens") + xlab("Number of patients in which an individual gene is perturbed") +
    geom_vline(aes(xintercept=100),
            color="black", linetype="dashed", size=1) +
    scale_x_continuous(breaks=seq(0,1400,100)) +
    theme_dark() + theme(panel.background = element_rect(fill = "white")) +
    theme(plot.background = element_rect(fill = "white", colour = "white")) +
    theme(axis.title.x = element_text(colour = "black", size = 10)) +
    theme(axis.title.y = element_text(colour = "black", size = 10)) +
    theme(axis.text = element_text(color= "black", size = 10)) +
    theme(axis.line = element_line(color = "black"))
histogramplot_uc_tf
```


```
png("Figure6_b.png",width=8, height=4, units="in", res=600)
histogramplot_uc_tf
dev.off()
```


```
null device 
          1
```


LS0tDQp0aXRlbDogIkZpZ3VyZSA2YiINCm91dHB1dDogaHRtbF9ub3RlYm9vaw0Ka25pdDogcmVwcmV4OjpyZXByZXhfcmVuZGVyDQotLS0NCg0KMS4gUmVtb3ZlIGFueXRoaW5nIGxlZnQgaW4gdGhlIGRhdGFmaWxlcw0KYGBge3J9DQpybShsaXN0PWxzKCkpDQpgYGANCklmIG5lY2VzZWFycnkgcGxlYXNlIGluc3RhbGwgdGhlIGZvbGxvd2luZyBwYWNrYWdlczoNCg0KaWYgKCFyZXF1aXJlTmFtZXNwYWNlKCJCaW9jTWFuYWdlciIsIHF1aWV0bHkgPSBUUlVFKSkNCiAgICBpbnN0YWxsLnBhY2thZ2VzKCJCaW9jTWFuYWdlciIpDQpCaW9jTWFuYWdlcjo6aW5zdGFsbCgiY2x1c3RlclByb2ZpbGVyIikNCkJpb2NNYW5hZ2VyOjppbnN0YWxsKCJSZWFjdG9tZVBBIikgDQpCaW9jTWFuYWdlcjo6aW5zdGFsbCgicnJ2Z28iKQ0KQmlvY01hbmFnZXI6Omluc3RhbGwoImVucmljaHBsb3QiKQ0KQmlvY01hbmFnZXI6Omluc3RhbGwoIm1zaWdkYnIiKQ0KQmlvY01hbmFnZXI6Omluc3RhbGwoIm9yZy5Icy5lZy5kYiIpDQpCaW9jTWFuYWdlcjo6aW5zdGFsbCgiQW5ub3RhdGlvbkRiaSIpDQppbnN0YWxsLnBhY2thZ2VzKCJnZ3Bsb3QyIikNCmluc3RhbGwucGFja2FnZXMoImdsdWUiKQ0KDQoyLiBSZWFkaW5nIGluIG5lY2VzZWFycnkgcGFja2FnZXMNCmBgYHtyfQ0KbGlicmFyeShjbHVzdGVyUHJvZmlsZXIpDQpsaWJyYXJ5KFJlYWN0b21lUEEpDQpsaWJyYXJ5KHJydmdvKQ0KbGlicmFyeShlbnJpY2hwbG90KQ0KbGlicmFyeShnZ3Bsb3QyKQ0KbGlicmFyeShtc2lnZGJyKQ0Kb3JnYW5pc20gPSJvcmcuSHMuZWcuZGIiDQpsaWJyYXJ5KG9yZ2FuaXNtLCBjaGFyYWN0ZXIub25seSA9IFRSVUUpDQpsaWJyYXJ5KGdsdWUpDQpsaWJyYXJ5KG11bGVhKQ0KbGlicmFyeSh0aWR5dmVyc2UpDQpsaWJyYXJ5KEFubm90YXRpb25EYmkpDQpsaWJyYXJ5KHNjYWxlcykNCmBgYA0KMy4gRG9pbmcgdGhlIHNhbWUgZm9yIFVDIFRGLVRHDQpgYGB7cn0NCm91dGNvbWV0ZiA8LSByZWFkLmNzdigidWNfVEZfVEdfbmV0d29ya19zdW1tYXJ5XzEway50eHQiLCBzZXA9Ilx0Iiwgcm93Lm5hbWVzID0gMSkNCmBgYA0KYGBge3J9DQpoZWFkKG91dGNvbWV0ZikNCmBgYA0KICAgICAgICAgICAgICAgICAgICAgICAgICANCg0KYGBge3J9DQphZmZlY3RlZF9URl9UR19VQyA8LSBvdXRjb21ldGZbb3V0Y29tZXRmJFpfQ291bnRfVEZURz4wLF0NCmBgYA0KDQpWaXN1YWxpc2luZyB0aGUgZGlzdHJpYnV0aW9uIG9mIGFmZmVjdGVkIFRHcyBwZXIgVEYgaW4gVUMNCg0KYGBge3J9DQpoaXN0b2dyYW1wbG90X3VjX3RmIDwtIGdncGxvdChhZmZlY3RlZF9URl9UR19VQywgYWVzKHg9Wl9Db3VudF9URlRHKSkgKyANCiAgICBnZW9tX2hpc3RvZ3JhbShjb2xvdXI9IndoaXRlIiwgZmlsbD0iZ3JlZW4iLCBiaW53aWR0aCA9IDUwKSsNCiAgICB5bGFiKCJOdW1iZXIgb2YgaW5kaXZpZHVhbCB0YWdldCBnZW5zIikgKyB4bGFiKCJOdW1iZXIgb2YgcGF0aWVudHMgaW4gd2hpY2ggYW4gaW5kaXZpZHVhbCBnZW5lIGlzIHBlcnR1cmJlZCIpICsNCiAgICBnZW9tX3ZsaW5lKGFlcyh4aW50ZXJjZXB0PTEwMCksDQogICAgICAgICAgICBjb2xvcj0iYmxhY2siLCBsaW5ldHlwZT0iZGFzaGVkIiwgc2l6ZT0xKSArDQogICAgc2NhbGVfeF9jb250aW51b3VzKGJyZWFrcz1zZXEoMCwxNDAwLDEwMCkpICsNCiAgICB0aGVtZV9kYXJrKCkgKyB0aGVtZShwYW5lbC5iYWNrZ3JvdW5kID0gZWxlbWVudF9yZWN0KGZpbGwgPSAid2hpdGUiKSkgKw0KICAgIHRoZW1lKHBsb3QuYmFja2dyb3VuZCA9IGVsZW1lbnRfcmVjdChmaWxsID0gIndoaXRlIiwgY29sb3VyID0gIndoaXRlIikpICsNCiAgICB0aGVtZShheGlzLnRpdGxlLnggPSBlbGVtZW50X3RleHQoY29sb3VyID0gImJsYWNrIiwgc2l6ZSA9IDEwKSkgKw0KICAgIHRoZW1lKGF4aXMudGl0bGUueSA9IGVsZW1lbnRfdGV4dChjb2xvdXIgPSAiYmxhY2siLCBzaXplID0gMTApKSArDQogICAgdGhlbWUoYXhpcy50ZXh0ID0gZWxlbWVudF90ZXh0KGNvbG9yPSAiYmxhY2siLCBzaXplID0gMTApKSArDQogICAgdGhlbWUoYXhpcy5saW5lID0gZWxlbWVudF9saW5lKGNvbG9yID0gImJsYWNrIikpDQpoaXN0b2dyYW1wbG90X3VjX3RmDQpgYGANCg0KYGBge3J9DQpwbmcoIkZpZ3VyZTZfYi5wbmciLHdpZHRoPTgsIGhlaWdodD00LCB1bml0cz0iaW4iLCByZXM9NjAwKQ0KaGlzdG9ncmFtcGxvdF91Y190Zg0KZGV2Lm9mZigpDQpgYGANCg==
